# Thermoneutral N−H Bond Activation of Ammonia by a Geometrically Constrained Phosphine

**DOI:** 10.1002/anie.202111017

**Published:** 2021-09-29

**Authors:** Josh Abbenseth, Oliver P. E. Townrow, Jose M. Goicoechea

**Affiliations:** ^1^ Department of Chemistry University of Oxford 12 Mansfield Road OX1 3TA Oxford UK

**Keywords:** ammonia activation, phosphorus heterocycles, pincer ligands, small molecule activation, sulfur heterocycles

## Abstract

A geometrically constrained phosphine bearing a tridentate NNS pincer ligand is reported. The effect of the geometric constraint on the electronic structure was probed by theoretical calculations and derivatization reactions. Reactions with N−H bonds result in formation of cooperative addition products. The thermochemistry of these transformations is strongly dependent on the substrate, with ammonia activation being thermoneutral. This represents the first example of a molecular compound that reversibly activates ammonia via N−H bond scission in solution upon mild heating.

## Graphical abstract

A geometrically constrained phosphine bearing an NNS pincer ligand is reported. The steric and electronic parameters were tuned to allow for unprecedented reversible ammonia activation via N−H bond scission. The phosphine's electronic structure and the associated reaction mechanism of N−H bond scission are elucidated by derivatization reactions and computational methods.

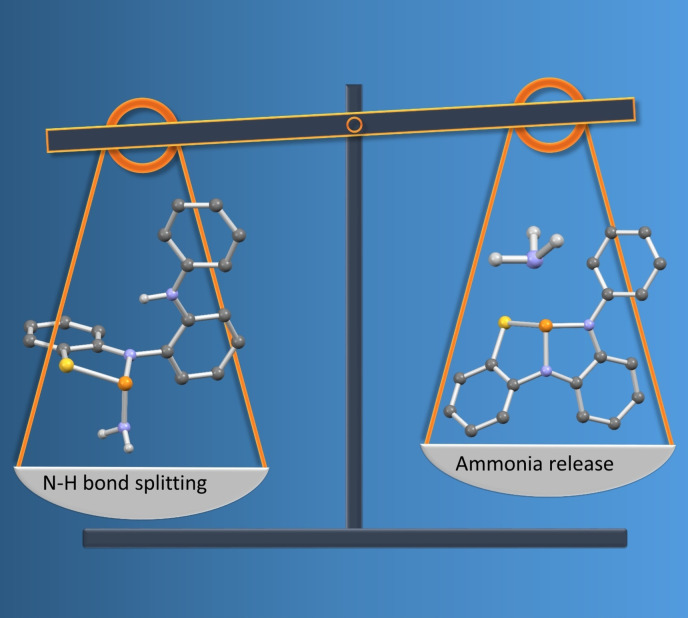

The activation of polar small molecules for the synthesis of value added chemicals has been a point of interest in transition metal chemistry for decades. While transition metal complexes are capable of catalytically converting numerous small molecules through E−H bond cleavage, ammonia still represents a challenging substrate due to its propensity to form Werner‐type complexes, resulting in unfavorable coordination/activation equilibria.[^1^, ^2^, ^3^] In fact, to date there are only two transition metal complexes known, both Ir pincer compounds, that allow for the oxidative addition of NH_3_ to give isolable, terminal metal amido‐hydride species.[^3j^, ^3n^] Main group species offer an attractive alternative to precious metals due to their higher crustal abundance, lower toxicity (in most cases) and reduced tendency to form unreactive ammonia adducts. While numerous examples of oxidative addition of N−H bonds to main group species have been reported over the last decade,[^4^, ^5^, ^6^, ^7^] N−H bond splitting of ammonia still poses a significant challenge and reversible activation of this indispensable nitrogen feedstock close to thermoneutrality remains unprecedented in all of molecular chemistry.

Geometrically constrained phosphines have been shown to exhibit pronounced ambiphilic reactivity due to an accessible lowest unoccupied molecular orbital (LUMO) featuring empty p orbital type character at the phosphorus center. This allows access to hydrido amino phosphoranes upon reaction with N−H bonds, as initially demonstrated by Radosevich and co‐workers (Scheme 1 A).^[7d]^ However, the two‐electron oxidation towards phosphorus(V) renders these products thermodynamically overstabilized. A recent report by Kinjo and co‐workers showed that cooperative addition along phosphorus ligand bonds is also viable (Scheme 1 B).^[7e]^ The presence of an enamine group in **IV** resulted in an isomerization to the thermodynamically favored imine **V**, which precludes the possibility of using this species in catalytic transformations.^[8]^

**Scheme 1:**
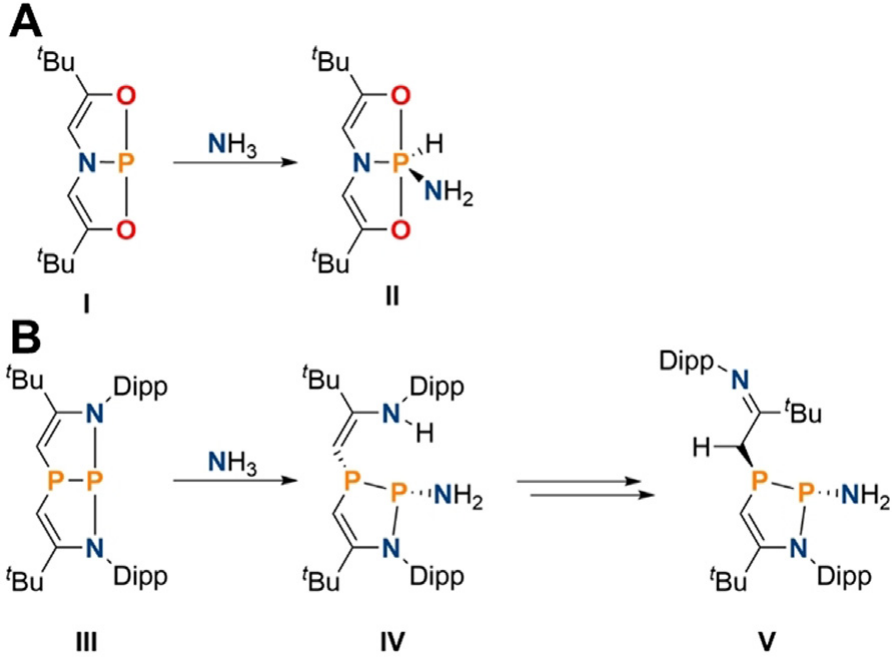
A) First example of ambiphilic ammonia activation by the geometrically constrained phosphine **I** to give **II**. B) Activation of ammonia by **III** via cooperative addition along a P−N bond forming **IV**, and its isomerization to afford **V** (Dipp=2,6‐^*i*^Pr‐C_6_H_3_).

We set out to design a ligand to host a phosphorus center which is selective for the cooperative activation of ammonia over formal oxidative addition and does not undergo follow‐up isomerization. For this purpose, the asymmetric NNS ligand **1** was synthesized in a three‐step procedure starting from commercially available *N*‐phenyl‐*o*‐phenylenediamine in 62 % yield (Figure 1 A).^[9]^ The reduced basicity of arylamines in contrast to alkyl derivatives and the presence of a sulfur donor is expected to benefit reversible N−H bond formation and disfavor the formation of phosphorus(V) species. Upon heating a mixture of **1**, PCl_3_ and NEt_3_ in tetrahydrofuran, the geometrically constrained phosphine **2** can be obtained in almost quantitative yield. **2** features a chemical shift in its ^31^P{^1^H} NMR spectrum at +165.9 ppm. The molecular structure of **2** in the solid state shows a strongly distorted phosphine, with bond angles of the phosphorus atoms to the proximal ligands close to 90°, while the N1‐P1‐S1 angle is significantly larger (109.74(6)°, Figure 1 B). The P−N bond lengths (*d*_P−N_=1.7012(15), 1.7215(16) Å) resemble those of the related phosphorus triamine P(κ^3^‐*N*,*N*,*N*‐N{2‐NCH_3_‐C_6_H_4_}_2_) (*d*_P−N_=1.7014(14), 1.7190(13) Å).^[7c]^ The computed highest occupied molecular orbital (HOMO) is delocalized over the ligand framework and the phosphorus center with the main contribution stemming from a p type orbital of the sulfur atom. In accordance with the induction of biphilicity upon geometric constraint, the LUMO mainly consists of an empty p type orbital of the phosphorus which allows for electrophilic reactivity towards substrates (Figure 1 C).

**Figure 1:**
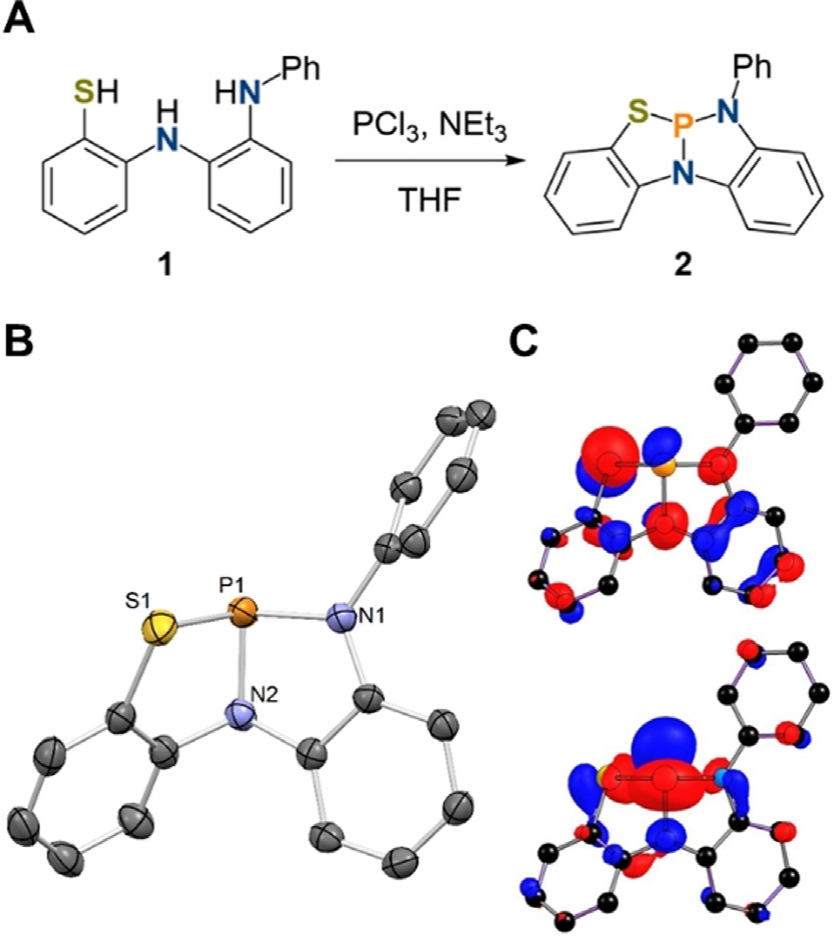
A) Synthesis of **2**; B) Molecular structure of **2** in the solid state (thermal ellipsoids at the 50 % probability level, one molecule of the asymmetric unit shown); hydrogen atoms omitted for clarity. Selected bond lengths [Å] and angles [°]: **2** P1–N1 1.7012(16), P1–N2 1.7215(16), P1–S1 2.2422(7); N1‐P1‐N2 91.22(8), N1‐P1‐S1 109.74(6), N2‐P1‐S1 92.26(6). C) Computed HOMO (top) and LUMO (bottom) of **2**, BP86‐D3/def2TZVP//SDDALL/6–31g**.^[9]^

The effect of geometric constraint on the s‐orbital character of the phosphorus lone pair in **2** can be probed via formation of the selenophosphorane **3** upon stirring with an excess of elemental selenium in CDCl_3_ (Figure 2). **3** features a large phosphorus‐selenium coupling constant of ^1^ *J*_P−Se_=924 Hz indicative of significantly increased s‐character of the phosphorus lone‐pair in comparison to classical phosphines.^[10]^ The molecular structure in the solid state shows a distorted phosphorus center featuring a P=Se bond length of 2.0643(7) Å (Figure 2 B) which relates well with the previously structurally related selenophosphorane P(Se)(κ^3^‐*N*,*N*,*N*‐N{2‐NCH_3_‐C_6_H_4_}_2_) (*d*_P−Se_=2.0718(11) Å).^[7c]^ The low Lewis‐basicity of **2** is further demonstrated upon addition of B(C_6_F_5_)_3_ to a solution of **2** in tetrahydrofuran which does not lead to any observable changes in the ^31^P{^1^H} or ^11^B{^1^H} NMR spectra. However, when the solvent is exchanged for benzene a significant broadening and minor NMR signal shifts are observed in the ^31^P{^1^H}, ^19^F{^1^H} and ^11^B{^1^H} NMR spectra upon subsequent addition of 0.5 and 1.0 equiv. of borane suggesting an equilibrium reaction. Slow evaporation of the reaction mixture at room temperature yielded crystals suitable for single crystal X‐ray diffraction confirming Lewis adduct, **4**, which exhibits a P−B bond distance of 2.0823(19) Å (Figure 2 C). When compared to **3**, a minor elongation of the phosphorus ligand bonds (ca. 0.01 Å) is observed due to the different oxidation states of the phosphorus center. Coordination of the borane to the sulfur atom was computed to be thermodynamically unfavorable.^[9]^

**Figure 2:**
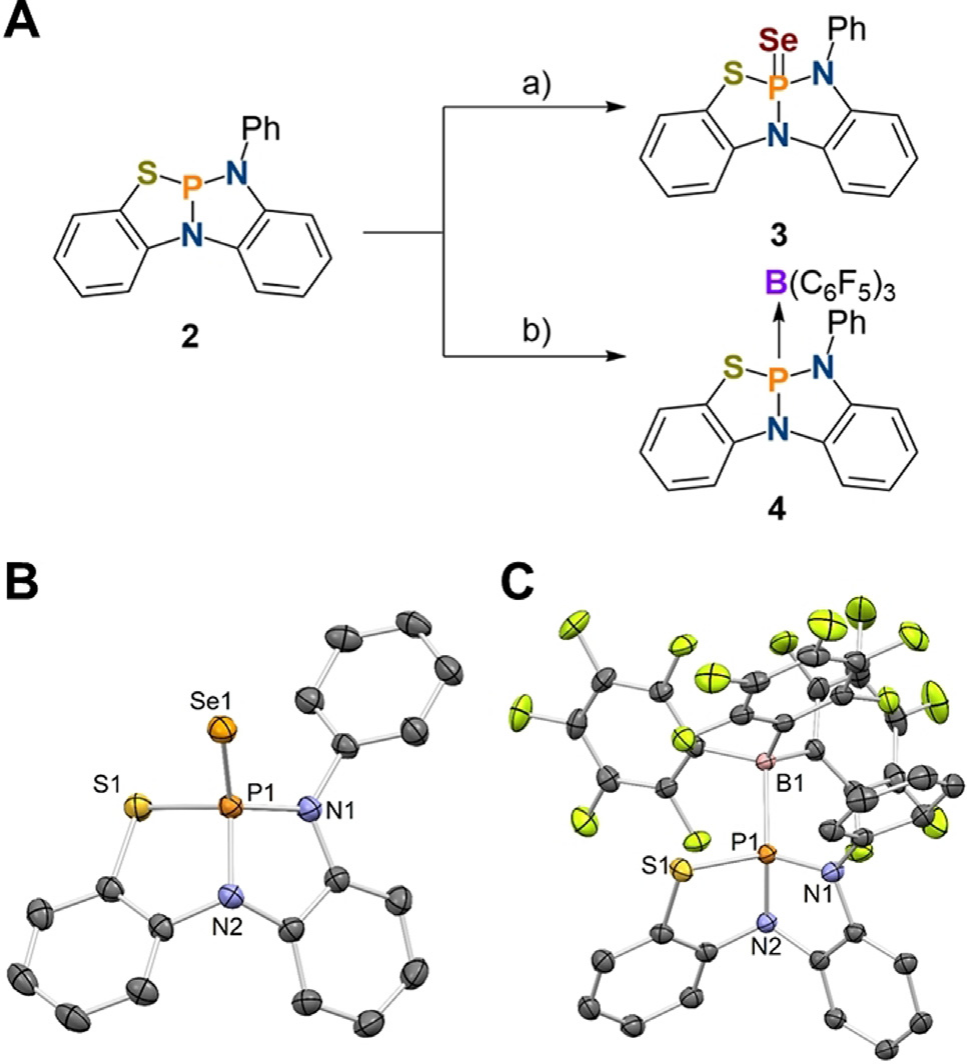
A) Synthesis of **3** and **4** from **2**: a) 5 equiv. Se, CDCl_3_, 5 d, 25 °C; b) 1 equiv. B(C_6_F_5_)_3_, C_6_D_6_, 16 h, 25 °C; Molecular structure of **3** (B) and **4** (C) in the solid state (thermal ellipsoids set at the 50 % probability level); hydrogen atoms omitted for clarity. Selected bond lengths [Å] and angles [°]: **3** P1–N1 1.667(2), P1–N2 1.700(2), P1–S1 2.1263(10), P1–Se1 2.0643(7); N1‐P1‐N2 93.05(12), N1‐P1‐S1 115.74(9), N2‐P1‐S1 95.59(9); **4** P1–N1 1.6793(16), P1–N2 1.7126(15), P1–S1 2.1181(6), P1–B1 2.0823(19); N1‐P1‐N2 93.80(8), N1‐P1‐S1 116.62(6), N2‐P1‐S1 93.35(5).^[9]^

Addition of one equivalent of *para*‐anisidine to a solution of **2** showed almost no conversion after 24 h, however when repeating the reaction with an excess of reagent (10 equiv.) cooperative addition along the P−N bond is observed as suggested by ^31^P and ^1^H NMR spectroscopy (**5 a**: 88.4 ppm, ^2^ *J*_P−H_=16 Hz, **5 b**: 83.5 ppm, ^2^ *J*_P−H_=11 Hz, Scheme 2), while two electron oxidation towards phosphorus(V) was not detected. The incomplete conversion (66 %) of **2** towards **5 a**/**b** when being exposed to an excess of substrate indicates an overall endergonic process. By analogy to the reported reactivity of P(κ^3^‐*N*,*N*,*N*‐N{2‐NCH_3_‐C_6_H_4_}_2_) towards E−H bonds, the observation of two isomers is rationalized by a hindered rotation about the N−C bond which results in slow conversion between **5 a** and **5 b** on the NMR timescale. The 1:2 ratio of the isomers observed by NMR spectroscopy stems from unfavourable steric interactions in the case of **5 a**.^[7c]^ Consequently, both isomers and the excess aniline exchange in solution as shown by ^1^H EXSY NMR spectroscopy. The endergonic nature of the reaction is confirmed by theoretical calculations which disfavour two electron oxidation of the phosphorus while cooperative addition is computed to be close to thermoneutrality.[^9^, ^11^]

**Scheme 2:**
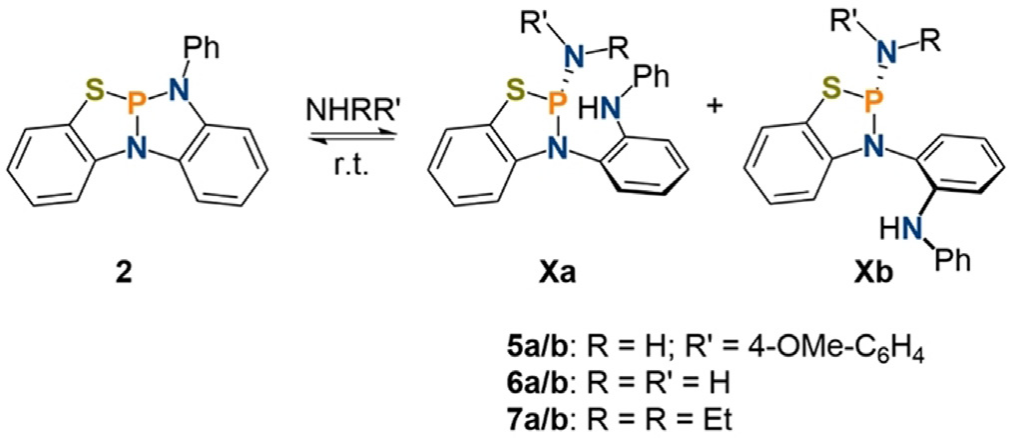
Reaction of **2** with amines to give **5 a**/**b**, **6 a**/**b** and **7 a**/**b**.

The reaction of **2** with ammonia in tetrahydrofuran resulted in the immediate and quantitative formation of the aminophosphines **6 a**/**b** in a 1:1.1 ratio (**6 a**: 90.5 ppm, ^2^ *J*_P−H_=15.2 Hz, **6 b**: 88.0 ppm, ^2^ *J*_P−H_=14.8 Hz, *ν*_N−H_=3398, 3320 cm^−1^, Scheme 2) in stark contrast to the reported reactivity of **III**. In the system reported by Kinjo and co‐workers ammonia activation is non‐quantitative over the course of 16 h with a computed activation barrier of (Δ*G* ^≠^_DFT_ *=*28.2 kcal mol^−1^).^[7e]^ After removal of the excess ammonia, the phosphorus NMR spectrum shows reformation of **2** in small amounts (up to 21 %) accompanied by free NH_3_ which can be detected in solution. The reversibility of the ammonia activation at room temperature could be shown by reaction of **6 a**/**b** with one equivalent of B(C_6_F_5_)_3_ which regenerates **2** and forms the ammonia borane adduct.[^12^, ^13^] Exposing **2** to an ammonia atmosphere (i.e. an excess of ammonia) in the presence of B(C_6_F_5_)_3_ does not influence the formation of **6 a**/**b**. Heating of **6 a**/**b** to 60 °C in tetrahydrofuran under an argon atmosphere results in the regeneration of **2** (59 %) accompanied by the release of NH_3_. Upon cooling to room temperature, reformation of **6 a**/**b** occurs. When heated in solution under a static vacuum full conversion towards **2** can be detected.^[9]^ This represents the first report of reversible N−H bond scission and release of ammonia by a molecular compound triggered by heating without the need of further stimuli, for example, vacuum or addition of external reagents.

Recent computational studies have shown that multiple pathways for the cooperative activation of ammonia along phosphorus‐ligand bonds can be envisioned, depending on ammonia concentration, solvent and temperature.^[14]^ By analogy with the results obtained for **III**, cooperative addition of ammonia along the P−N bond in **2** was found to be associated with an activation energy of Δ*G* ^≠^_DFT_ *=*22.0 kcal mol^−1^ in tetrahydrofuran at room temperature, rationalizing the significantly enhanced reaction rate when compared to **III**.^[7e]^ In accordance with the observed reversibility in solution, the free reaction energy was computed to be essentially thermoneutral at room temperature and slightly endergonic at 60 °C: Δ*G*_DFT_ (kcal mol^−1^)=−0.4 (25 °C)/0.6 (60 °C) (**6 a**); 0.0 (25 °C)/1.1 (60 °C) (**6 b**). Oxidative addition, producing the phosphorane **6 c**, was computed to be endergonic (Δ*G*_DFT_=4.91 kcal mol^−1^) with an activation energy of Δ*G* ^≠^_DFT_ *=*56.0 kcal mol^−1^. The isomerization of **6 c** towards **6 a** in the presence of ammonia was calculated to be associated with an activation energy of Δ*G* ^≠^_DFT_=28.7 kcal mol^−1^ and consequently not operative at room temperature.^[9]^ Therefore, further possible mechanisms[^7c^, ^14^] for the production of **6 a** from **6 c**, were not considered. The relaxed surface scan of the N−H bond activation and analysis via intrinsic bonding orbital (IBO) analysis,^[15]^ reveals that a planarization of the phosphorus center precedes the coordination of ammonia (Figure 3 B). The activation barrier for this bending mode, in the absence of ammonia, was calculated to be Δ*G* ^≠^_DFT_ *=*10.8 kcal mol^−1^ in tetrahydrofuran, which relates well with the experimentally derived value for the planarization of P(κ^3^‐*N*,*N*,*N*‐N{2‐N^*i*^Pr‐C_6_H_4_}_2_) (Δ*G* ^≠^=10.7(5) kcal mol^−1^).^[7c]^ The adjacent donor atoms around the phosphorus center stabilize this T‐shape due to π‐donation into the empty p‐type orbital located at the phosphorus center. In a concerted σ‐metathesis mechanism the N−H bond of ammonia is being elongated and acidified by association of the nitrogen lone pair with the empty phosphorus orbital accompanied by P–N_ligand_ bond cleavage (Figure 3 C). This allows for deprotonation of the coordinated NH_3_ by the flanking bisarylamide resulting in the formation of aminophosphine **6 a**.^[9]^ Although the P−S bond in **2** is comparably weaker than the P−N bonds, no suitable pathway for ammonia activation could be identified via DFT due to an overall highly endergonic process of cooperative ammonia splitting along the P−S bond due to the reduced basicity of the sulfur donor and the low bond strength of the resulting S−H bond (Δ*G*_DFT_=6.7 kcal mol^−1^).

**Figure 3:**
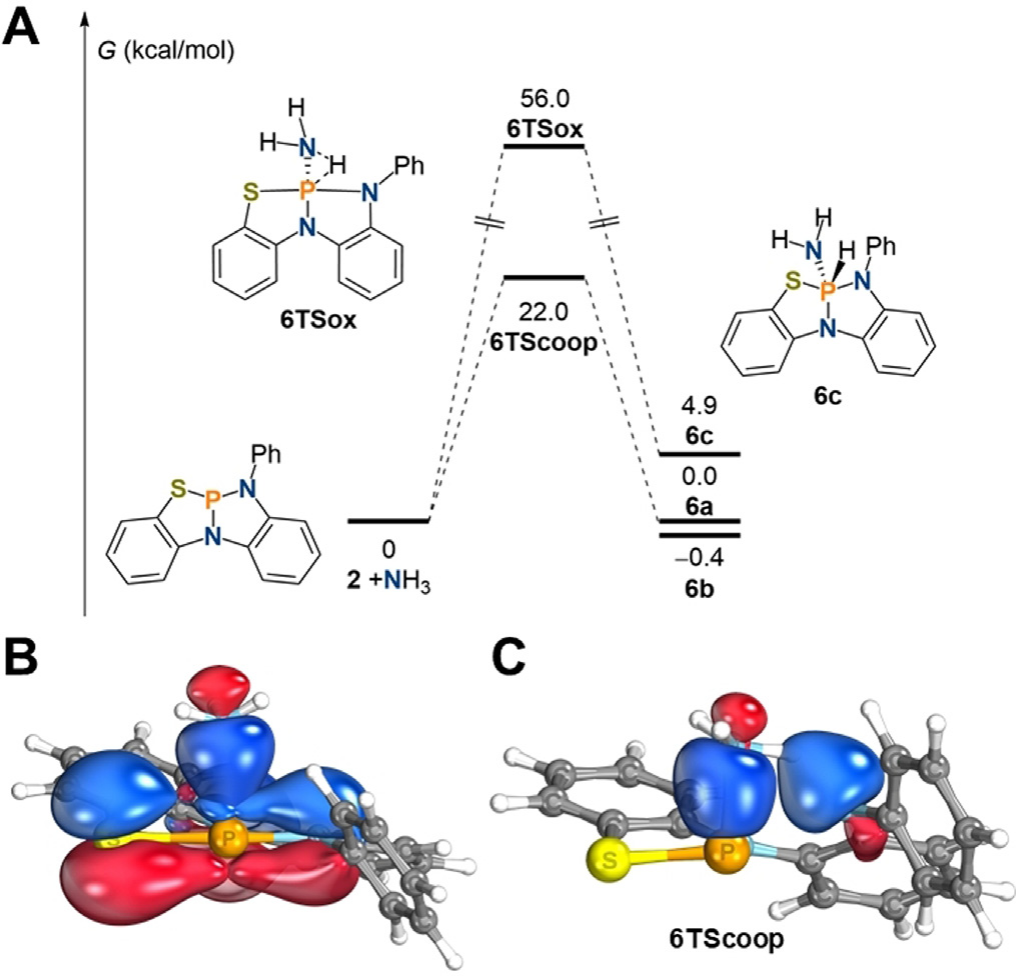
A) Computed mechanisms of N−H bond activation by **2** to produce **6 a**–**c**; IBO analysis of the planarization of **2** upon approach of NH_3_ (B) and transition state of ammonia activation displaying participating nitrogen lone pairs (C).

With an endergonic and a thermoneutral N−H bond activation in hand, we set out to study the reaction of **2** with an electron rich alkylamine to obtain an exergonic and irreversible N−H bond activation. The reaction of **2** with HNEt_2_ resulted in the clean formation of the amine **7 a**/**b** in a 1:3.7 ratio (**7 a**: 105.6 ppm, ^3^ *J*_P−H_=9.4 Hz, **7 b**: 98.2 ppm, ^3^ *J*_P−H_=9.1 Hz, *ν*_N−H_=3401 cm^−1^, Scheme 2). The molecular structure of **7 b** in the solid state (Figure 4) confirms the proposed structures of the N−H bond activation products. The diethylamine substituent features a planarized nitrogen atom (angle sum: 356°) indicating donation of the nitrogen lone‐pair into a σ* orbital.^[7c]^ The formed 1,3,2‐thiazaphosphole exhibits a flattened structure with the N‐C‐C‐S moiety being strictly planar due to π delocalization in the aromatic system of the ligand backbone. When **7** is exposed to vacuum minor reformation of **2** is observed (1–9 %), consistent with an overall computed free reaction energy of Δ*G*_DFT_=−4.0 (**7 a**) and −3.3 (**7 b**) kcal mol^−1^ in tetrahydrofuran at 25 °C.

**Figure 4:**
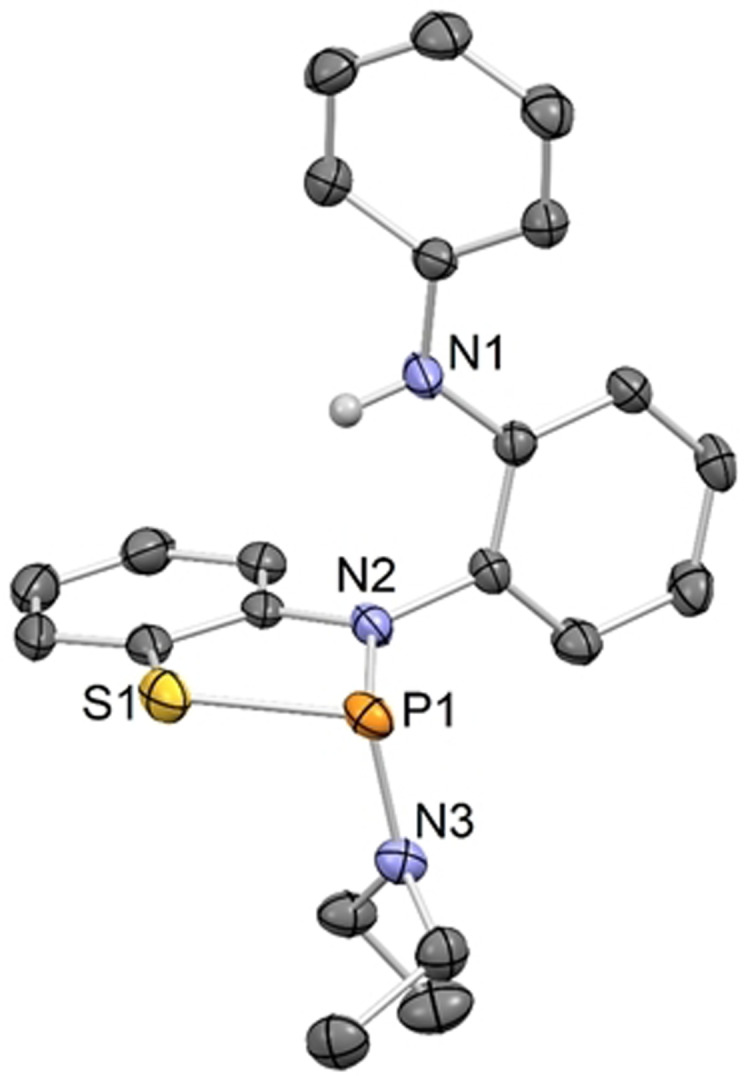
Molecular structure of **7 b** in the solid state (thermal ellipsoids set at the 50 % probability level); hydrogen atoms, except the one bound to N1, omitted for clarity. Selected bond lengths [Å] and angles [°]: P1–N2 1.7211(14), P1–N3 1.6750(14), P1–S1 2.1686(6); N2‐P1‐S1 89.57(5), N2‐P1‐N3 102.81(7), N3‐P1‐S1 107.62(5).^[9]^

In conclusion, we report the synthesis of the new geometrically constrained phosphine **2** bearing an asymmetric NNS pincer ligand. The structural rigidity of the pincer scaffold results in a HOMO with pronounced s‐orbital character and a low lying phosphorus centered LUMO. The activation of N−H bonds was shown to result in cooperative activation along the flanking P−N bond of **2** with the overall thermochemistry being highly dependent on the amine used, with ammonia activation being instant, reversible and close to thermoneutrality in solution at room temperature. These results shows how geometrically constrained phosphines can be tuned to facilitate the thermodynamic parameters of E−H bond activation reactions that remain challenging for transition metal complexes and thereby open up new strategy for the catalytic production of organic molecules by small molecule activation reactions.

## Conflict of interest

The authors declare no conflict of interest.

## Supporting information

As a service to our authors and readers, this journal provides supporting information supplied by the authors. Such materials are peer reviewed and may be re‐organized for online delivery, but are not copy‐edited or typeset. Technical support issues arising from supporting information (other than missing files) should be addressed to the authors.

Supporting Information

Supporting Information
