# Supplementary material for: Thermoneutral N−H Bond Activation of Ammonia by a Geometrically Constrained Phosphine
Source: Angew Chem Int Ed Engl. 2021 Sep 29;60(44):23625–9. doi: 10.1002/anie.202111017 (PMC8596738; doi:10.1002/anie.202111017)

checkCIF/PLATON report

You have not supplied any structure factors. As a result the full set of tests cannot be run.

THIS REPORT IS FOR GUIDANCE ONLY. IF USED AS PART OF A REVIEW PROCEDURE FOR PUBLICATION, IT SHOULD NOT REPLACE THE EXPERTISE OF AN EXPERIENCED CRYSTALLOGRAPHIC REFEREE.

No syntax errors found.   CIF dictionary   Interpreting this report

Datablock: JA466

No errors found in this datablock

```
Bond precision:  C-C = 0.0030 A          Wavelength=1.54184

Cell:            a=9.6578(2)    b=12.5738(2)      c=24.4697(6)
                 alpha=90       beta=91.155(2)    gamma=90
Temperature:     150 K

                Calculated                  Reported
Volume          2970.88(11)                 2970.88(11)
Space group     P 21/n                      P 21/n
Hall group      -P 2yn                      -P 2yn
Moiety formula  C18 H13 N2 P S              C18 H13 N2 P S
Sum formula     C18 H13 N2 P S              C18 H13 N2 P S
Mr              320.33                      320.33
Dx,g cm-3       1.432                       1.432
Z               8                           8
Mu (mm-1)       2.914                       2.914
F000            1328.0                      1328.0
F000'           1335.95
h,k,lmax        12,15,30                    12,15,30
Nref            6206                        6178
Tmin,Tmax       0.811,0.943                 0.880,1.000
Tmin'           0.792

Correction method= # Reported T Limits: Tmin=0.880 Tmax=1.000
AbsCorr = MULTI-SCAN

Data completeness= 0.995          Theta(max)= 76.278

R(reflections)= 0.0350( 4727)     wR2(reflections)= 0.0907( 6178)

S = 1.032                 Npar= 397
```

Datablock: JA315

Bond precision: C-C = 0.0046 A   Wavelength=1.54184

Cell:	a=13.9642(7)	b=8.8709(3)	c=14.6930(7)
	alpha=90	beta=115.169(6)	gamma=90
Temperature:	150 K		

	Calculated	Reported
Volume	1647.29(15)	1647.29(15)
Space group	P 21/n	P 21/n
Hall group	-P 2yn	-P 2yn
Moiety formula	C18 H13 N2 P S Se	C18 H13 N2 P S Se
Sum formula	C18 H13 N2 P S Se	C18 H13 N2 P S Se
Mr	399.29	399.29
Dx,g cm-3	1.610	1.610
Z	4	4
Mu (mm-1)	5.178	5.178
F000	800.0	800.0
F000’	800.79	
h,k,lmax	17,11,18	17,11,18
Nref	3444	3409
Tmin,Tmax	0.426,0.733	0.644,1.000
Tmin’	0.278	

Correction method= # Reported T Limits: Tmin=0.644 Tmax=1.000
AbsCorr = MULTI-SCAN

Data completeness= 0.990   Theta(max)= 75.981

R(reflections)= 0.0416(3052)   wR2(reflections)= 0.1145(3409)

S = 1.036   Npar= 208

The following ALERTS were generated. Each ALERT has the format
test-name_ALERT_alert-type_alert-level.
Click on the hyperlinks for more details of the test.

Alert level C

PLAT230_ALERT_2_C Hirshfeld Test Diff for   Se1   --P1   .   7.0 s.u.

Alert level G

PLAT793_ALERT_4_G Model has Chirality at P1   (Centro SPGR)   S Verify
PLAT941_ALERT_3_G Average HKL Measurement Multiplicity ....   2.5 Low

---

0 **ALERT level A** = Most likely a serious problem - resolve or explain
0 **ALERT level B** = A potentially serious problem, consider carefully
1 **ALERT level C** = Check. Ensure it is not caused by an omission or oversight
2 **ALERT level G** = General information/check it is not something unexpected

0 ALERT type 1 CIF construction/syntax error, inconsistent or missing data

```
   1 ALERT type 2 Indicator that the structure model may be wrong or deficient
   1 ALERT type 3 Indicator that the structure quality may be low
   1 ALERT type 4 Improvement, methodology, query or suggestion
   0 ALERT type 5 Informative message, check
```

Datablock: JA455

```
Bond precision:  C-C = 0.0030 A         Wavelength=1.54184

Cell:            a=11.6192(2)     b=13.9694(2)      c=19.1350(3)
                 alpha=90         beta=97.109(1)    gamma=90
Temperature:     150 K

                Calculated                 Reported
Volume          3081.99(8)                 3081.99(8)
Space group     P 21/n                     P 21/n
Hall group      -P 2yn                     -P 2yn
Moiety formula  C36 H13 B F15 N2 P S       C36 H13 B F15 N2 P S
Sum formula     C36 H13 B F15 N2 P S       C36 H13 B F15 N2 P S
Mr              832.32                     832.32
Dx,g cm-3       1.794                      1.794
Z               4                          4
Mu (mm-1)       2.590                      2.590
F000            1656.0                     1656.0
F000'           1665.58
h,k,lmax        14,17,24                   14,17,24
Nref            6465                       6436
Tmin,Tmax       0.780,0.950                0.917,1.000
Tmin'           0.772

Correction method= # Reported T Limits: Tmin=0.917 Tmax=1.000
AbsCorr = MULTI-SCAN

Data completeness= 0.996          Theta(max)= 76.389

R(reflections)= 0.0400( 5473)     wR2(reflections)= 0.1109( 6436)

S = 1.036                 Npar= 505
```

The following ALERTS were generated. Each ALERT has the format
test-name_ALERT_alert-type_alert-level.
Click on the hyperlinks for more details of the test.

Alert level G

```
PLAT434_ALERT_2_G Short Inter HL..HL Contact F3       ..F9              2.67 Ang.
                                 3/2-x,1/2+y,1/2-z  =      2_655 Check
PLAT434_ALERT_2_G Short Inter HL..HL Contact F6       ..F14             2.73 Ang.
                                 1/2-x,1/2+y,1/2-z  =      2_555 Check
```

```
PLAT793_ALERT_4_G Model has Chirality at P1         (Centro SPGR)         S Verify
PLAT933_ALERT_2_G Number of OMIT Records in Embedded .res File ...        1 Note
```

---

0 **ALERT level A** = Most likely a serious problem - resolve or explain
0 **ALERT level B** = A potentially serious problem, consider carefully
0 **ALERT level C** = Check. Ensure it is not caused by an omission or oversight
4 **ALERT level G** = General information/check it is not something unexpected

0 ALERT type 1 CIF construction/syntax error, inconsistent or missing data
3 ALERT type 2 Indicator that the structure model may be wrong or deficient
0 ALERT type 3 Indicator that the structure quality may be low
1 ALERT type 4 Improvement, methodology, query or suggestion
0 ALERT type 5 Informative message, check

Datablock: JA469

Bond precision: C-C = 0.0024 A   Wavelength=1.54184

Cell:	a=11.0697(2)	b=13.5582(3)	c=13.8325(3)
	alpha=90	beta=104.197(2)	gamma=90
Temperature:	150 K		

	Calculated	Reported
Volume	2012.65(7)	2012.65(7)
Space group	P 21/n	P 21/n
Hall group	-P 2yn	-P 2yn
Moiety formula	C22 H24 N3 P S	C22 H24 N3 P S
Sum formula	C22 H24 N3 P S	C22 H24 N3 P S
Mr	393.47	393.47
Dx,g cm-3	1.299	1.299
Z	4	4
Mu (mm-1)	2.258	2.258
F000	832.0	832.0
F000’	836.35	
h,k,lmax	13,17,17	13,16,17
Nref	4204	4156
Tmin,Tmax	0.611,0.666	0.951,1.000
Tmin’	0.554	

Correction method= # Reported T Limits: Tmin=0.951 Tmax=1.000
AbsCorr = MULTI-SCAN

Data completeness= 0.989   Theta(max)= 76.118

R(reflections)= 0.0343(3448)   wR2(reflections)= 0.0897(4156)

S = 1.043   Npar= 250

```
The following ALERTS were generated. Each ALERT has the format
       test-name_ALERT_alert-type_alert-level.
Click on the hyperlinks for more details of the test.
```

Alert level G

```
PLAT941_ALERT_3_G Average HKL Measurement Multiplicity ..........        2.5 Low
```

```
   0 ALERT level A = Most likely a serious problem - resolve or explain
   0 ALERT level B = A potentially serious problem, consider carefully
   0 ALERT level C = Check. Ensure it is not caused by an omission or oversight
   1 ALERT level G = General information/check it is not something unexpected

   0 ALERT type 1 CIF construction/syntax error, inconsistent or missing data
   0 ALERT type 2 Indicator that the structure model may be wrong or deficient
   1 ALERT type 3 Indicator that the structure quality may be low
   0 ALERT type 4 Improvement, methodology, query or suggestion
   0 ALERT type 5 Informative message, check
```

It is advisable to attempt to resolve as many as possible of the alerts in all categories. Often the minor alerts point to easily fixed oversights, errors and omissions in your CIF or refinement strategy, so attention to these fine details can be worthwhile. In order to resolve some of the more serious problems it may be necessary to carry out additional measurements or structure refinements. However, the purpose of your study may justify the reported deviations and the more serious of these should normally be commented upon in the discussion or experimental section of a paper or in the "special_details" fields of the CIF. checkCIF was carefully designed to identify outliers and unusual parameters, but every test has its limitations and alerts that are not important in a particular case may appear. Conversely, the absence of alerts does not guarantee there are no aspects of the results needing attention. It is up to the individual to critically assess their own results and, if necessary, seek expert advice.

Publication of your CIF in IUCr journals

A basic structural check has been run on your CIF. These basic checks will be run on all CIFs submitted for publication in IUCr journals (*Acta Crystallographica*, *Journal of Applied Crystallography*, *Journal of Synchrotron Radiation*); however, if you intend to submit to *Acta Crystallographica Section C* or *E* or *IUCrData*, you should make sure that full publication checks are run on the final version of your CIF prior to submission.

Publication of your CIF in other journals

Please refer to the *Notes for Authors* of the relevant journal for any special instructions relating to CIF submission.

PLATON version of 13/07/2021; check.def file version of 13/07/2021

Datablock JA466 - ellipsoid plot

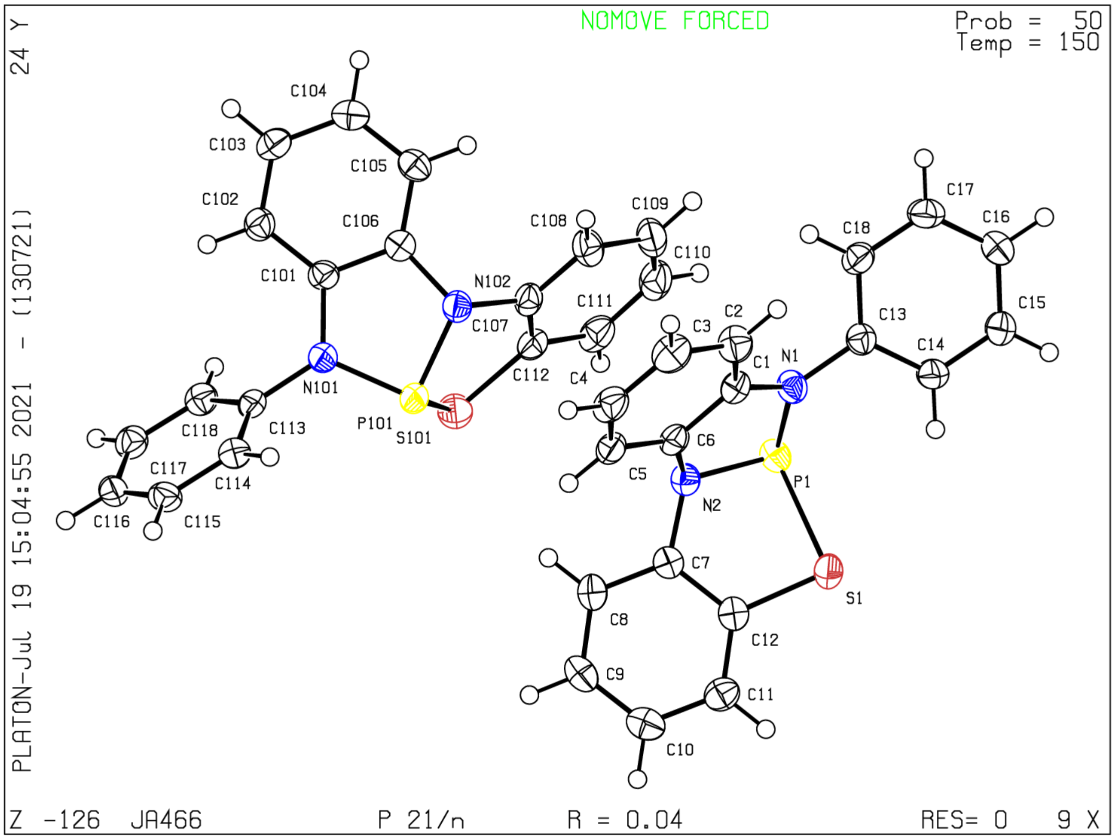

Datablock JA315 - ellipsoid plot

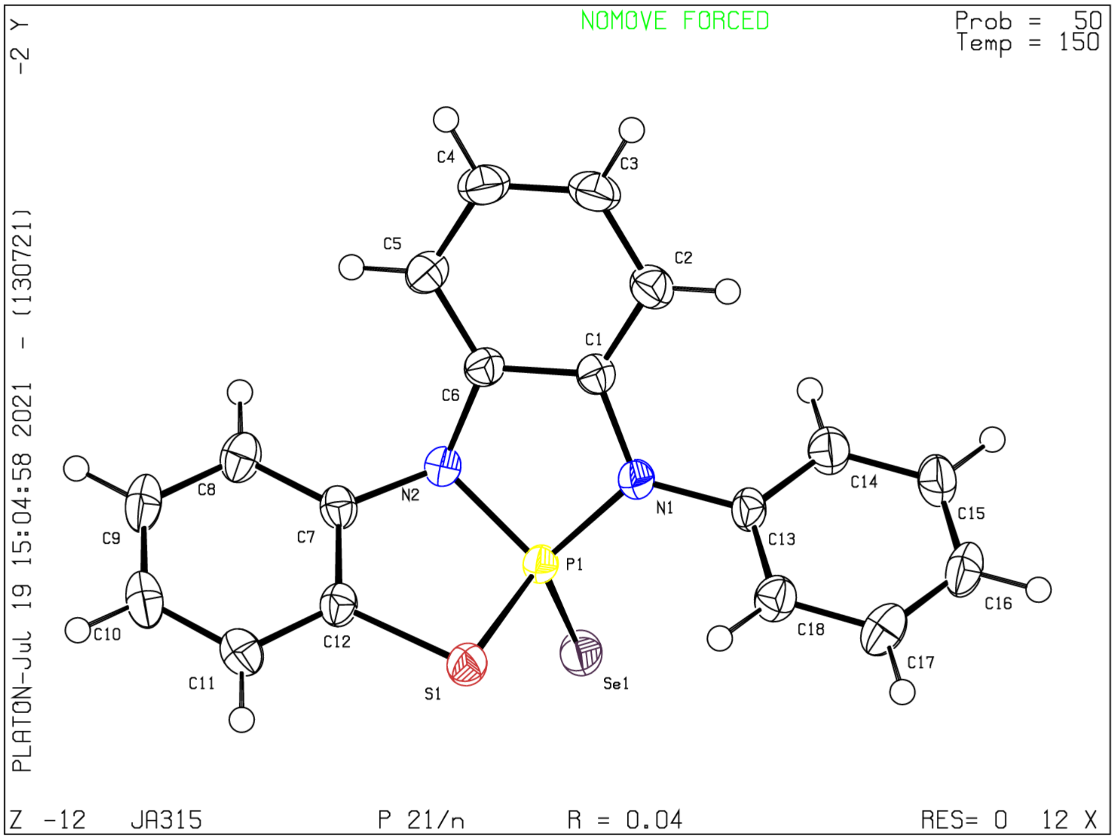

Datablock JA455 - ellipsoid plot

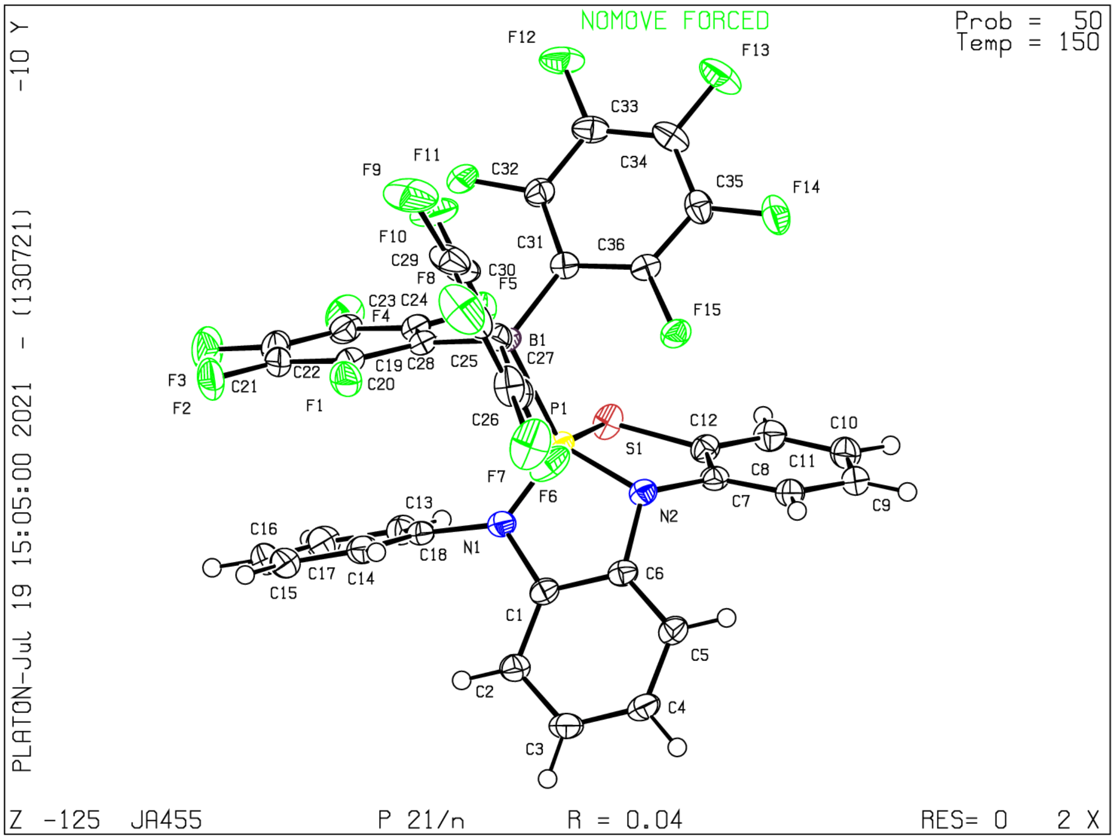

Datablock JA469 - ellipsoid plot

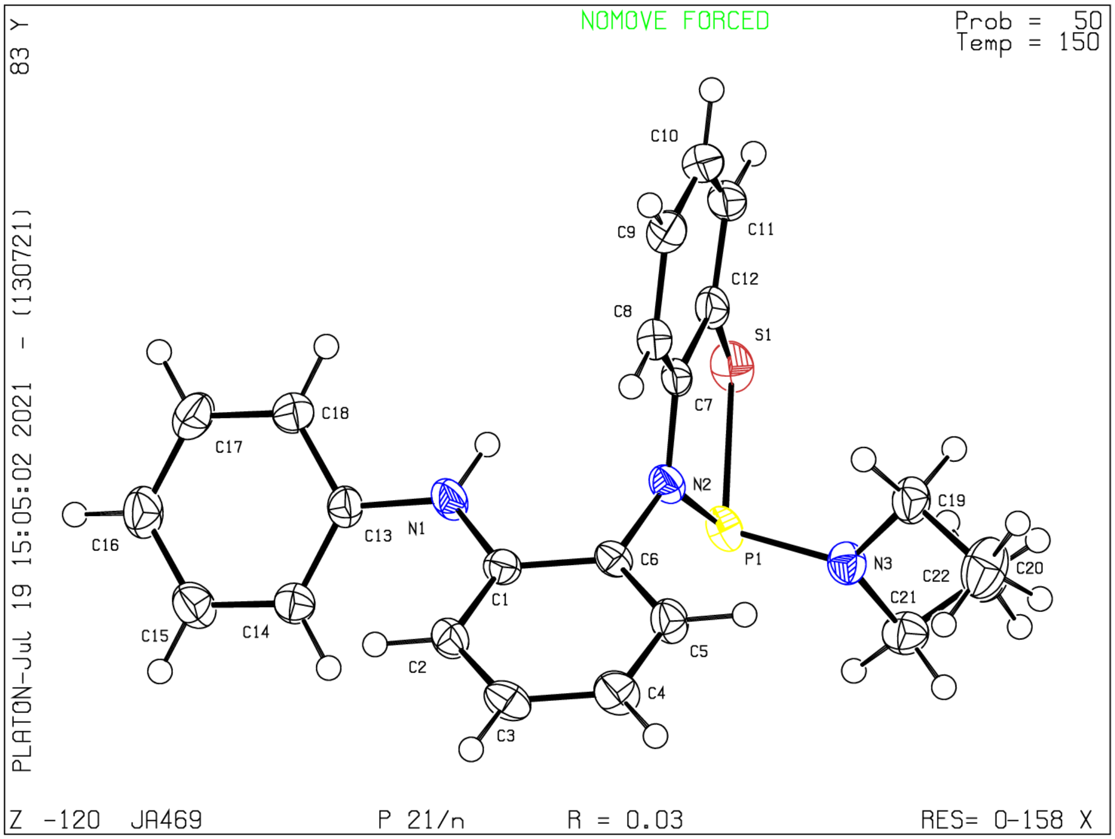

Supplement: Supplementary file 1 — Supporting Information [file ANIE-60-23625-s002.zip › checkcif.pdf]
