# Supplementary material for: Thermoneutral N−H Bond Activation of Ammonia by a Geometrically Constrained Phosphine
Source: Angew Chem Int Ed Engl. 2021 Sep 29;60(44):23625–9. doi: 10.1002/anie.202111017 (PMC8596738; doi:10.1002/anie.202111017)
Supplement: Supplementary file 2 — Supporting Information [file ANIE-60-23625-s001.pdf]

Angewandte Chemie
Eine Zeitschrift der Gesellschaft Deutscher Chemiker

Supporting Information

Thermoneutral N−H Bond Activation of Ammonia by a Geometrically Constrained Phosphine

Josh Abbenseth, Oliver P. E. Townrow, and Jose M. Goicoechea**

anie_202111017_sm_miscellaneous_information.pdf

Supporting Information

Thermoneutral N–H bond activation of ammonia by a geometrically constrained phosphine

Josh Abbenseth*, Oliver P. E. Townrow, Jose M. Goicoechea*

Department of Chemistry
University of Oxford
Chemistry Research Laboratory, 12 Mansfield Rd.
Oxford OX1 3TA, United Kingdom
E-mail: josh.abbenseth@chem.ox.ac.uk, jose.goicoechea@chem.ox.ac.uk

Contents

Experimental Details 2
Materials and General Procedures 2
Synthesis 3
N^1-benzyl-N^1-phenylbenzene-1,2-diamine (SM1) 3
N^1-benzyl-N^2-(2-(methylthio)phenyl)-N^1-phenylbenzene-1,2-diamine (SM2) 3
Synthesis of **1** 4
Synthesis of **2** 4
Synthesis of **3** 5
Reaction of **2** with $B(C_6F_5)_3$ to generate **4** 5
Reaction of **2** with para-anisidine to generate **5a/b** 6
Reaction of **2** with ammonia to generate **6a/b** 6
Synthesis of **7a/b** 7
Analytical data 8
Single crystal X-ray diffraction data 34
Computational Details 35
DFT optimised geometries 39
Coordinates of the computed species 60
References 93

Experimental Details

Materials and General Procedures

All reactions and product manipulations were carried out under an inert atmosphere of argon or dinitrogen using standard Schlenk-line or glovebox techniques (MBraun UNIlab glovebox maintained at < 0.1 ppm H_2O and < 0.1 ppm O_2). Pentane, benzene, toluene and THF (Sigma-Aldrich, HPLC grade, 99.8%), were purified using an MBraun SPS-800 solvent system. C_6D_6 (Sigma-Aldrich, 99.5%) and THF-d_8 (Sigma-Aldrich, 99.5%) were distilled over sodium metal/benzophenone. $CDCl_3$ (Sigma-Aldrich, 99.5%) was dried over CaH_2 and distilled. All dry solvents were stored under argon in gas-tight ampoules over activated 3 Å molecular sieves. All solvents used for synthesis that did not require exclusion of moisture and/or air were used as received (Sigma-Aldrich, HPLC grade, 99.8%). *N*^{1}-Phenylbenzene-1,2-diamine (Sigma-Aldrich), benzylbromide (TCI), NaO^{t}Bu (Sigma-Aldrich), KO^{t}Bu (Sigma-Aldrich), $Pd_2(dba)_3$ (Sigma-Aldrich), PCy_3 (Fluorochem), 2-bromothioanisole (Fluorochem), sodium (Sigma-Aldrich), selenium (Sigma-Aldrich) and $B(C_6F_5)_3$ (TCI) were used as received. NH_3 (anhydrous, Linde) was used as received for the ligand synthesis. For reactions with the geometrically constrained phosphine **2**, ammonia was condensed in a flask containing sodium and stirred at –80°C until a brown color was obtained. NMR tubes containing **2** were exposed to an atmosphere of the dried ammonia at room temperature and shaken for 1 minute before NMR measurements. PCl_3 (Sigma-Aldrich) was distilled and degassed prior to use. NEt_3 (TCI) was degassed, dried over CaH_2 and distilled prior to use. *Para*-anisidine (Sigma-Aldrich) was sublimed and stored in a glove box prior to use. Diethylamine (Sigma-Aldrich) was degassed, distilled and stored over molecular sieves (3 Å). **NMR** samples were measured on a Bruker Avance III HD nanobay NMR equipped with a 9.4 T magnet and a Bruker Avance III NMR equipped with a 11.75 T magnet. The spectra were calibrated to the residual solvent proton resonance (C_6D_6: δ_H = 7.16 ppm, THF-d_8: δ_H = 3.58 ppm and $CDCl_3$: δ_H = 7.26 ppm). Air and moisture sensitive samples were prepared in the glovebox and measured in *J*-Young NMR tubes. **Mass spectrometry** was performed on an Agilent 6120 single quadrupole instrument connected to an Agilent 1260 IsoPumo and a CTC Analytics 2777 Sample Manager. **IR** spectra were recorded on a Nicolet iS5 infrared spectrometer equipped with a Nicolet 869-142300 iD3 ZnSe ATR module. **Single-crystal X-ray diffraction** data were collected using an Oxford Diffraction Supernova dual-source diffractometer equipped with a 135 mm Atlas CCD area detector. Crystals were selected under Paratone-N oil, mounted on micromount loops and quench-cooled using an Oxford Cryosystems open flow N_2 cooling device. Data were collected at 150 K using mirror monochromated Cu K_α (λ = 1.54184 Å) radiation and processed using the CrysAlisPro package, including unit cell parameter refinement and inter-frame scaling (which was carried out using SCALE3 ABSPACK within CrysAlisPro).[1] Structures were subsequently solved using direct methods.[2] In each case, the locations of the largest peaks in the final difference Fourier map calculations, as well as the magnitude of the residual electron densities, were of no chemical significance. Crystallographic data (excluding structure factors) for the structures reported in this paper have been deposited with the Cambridge Crystallographic Data Centre as a supplementary publication no. 2097495−2097498. Copies of the data can be obtained free of charge on application to CCDC, 12 Union Road, Cambridge CB21EZ, UK (fax: +(44)1223-336-033; email: deposit@ccdc.cam.ac.uk).

Synthesis

N^1-benzyl-N^1-phenylbenzene-1,2-diamine (SM1)

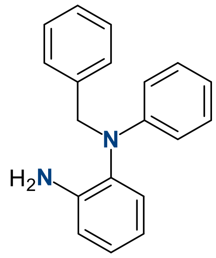

SM1

N^1-Phenylbenzene-1,2-diamine (1.50 g, 3.14 mmol, 1.00 eq.) and NaO^{t}Bu (939 mg, 3.77 mmol, 1.20 eq.) were mixed and cooled to –40°C in a 250 mL round bottom Schlenk flask. MeCN (100 mL) was added slowly under intense stirring and the resulting dark brown solution warmed to room temperature and stirred for 30 min. Benzyl bromide (1.16 mL, 3.77 mmol, 1.20 eq.) was added dropwise, resulting in precipitation of NaBr. The mixture was stirred at room temperature for 20 h. The solvent was removed and the obtained solid extracted with DCM (3 × 50 mL). The combined extracts were washed with water (2 × 50 mL) and brine (2 × 50 mL). The organic phase was dried with Na_2SO_4, filtered and reduced to dryness. The obtained crude product was purified by column chromatography. N^1-benzyl-N^1-phenylbenzene-1,2-diamine (1.72 g, 6.27 mmol, 77 %) was obtained as an off white powder. R_f (Toluene / Petrolether (40 – 60): 10/1): 0.3. NMR: ^{1}H (500.0 MHz, C_6D_6, 25°C) δ (ppm) = 7.23 – 7.21 (m, 2H), 7.11 – 7.01 (m, 6H), 6.98 – 6.95 (m, 1H), 6.75 – 6.70 (m, 3H), 6.66 – 6.62 (m, 1H), 6.43 – 6.41 (m, 1H), 4.57 (s, 2H, NCH_2), 3.15 (br, 2H, NCH_2); ^{13}C{^{1}H} (125.8 MHz, C_6D_6, 25°C) δ (ppm) = 148.4 (s, 1C), 144.5 (s, 1C), 139.6 (s, 1C), 133.1 (s, 1C), 129.7 (s, 1C), 129.6 (s, 2C), 128.8 (s, 2C), 127.8 (s, 1C), 127.3 (s, 2C), 127.0 (s, 1C), 119.3 (s, 1C), 118.3 (s, 1C), 116.4 (s, 1C), 114.2 (s, 2C), 55.8 (s, 2C, NCH_2); IR: (ATR, neat, cm^{-1}) ν = 3466 (br, m), 3371 (m), 1613 (m), 1595 (m), 1497 (s), 1355 (m), 1221 (m), 755 (m), 745 (m), 666 (m); ESI-MS (MeOH/DCM): 275.1 ([M+H]$^+$).

N^1-benzyl-N^2-(2-(methylthio)phenyl)-N^1-phenylbenzene-1,2-diamine (SM2)

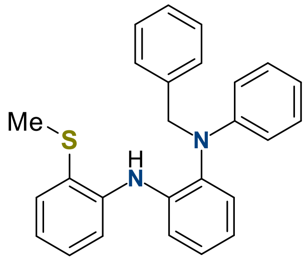

SM2

N^1-Phenylbenzene-1,2-diamine (687 mg, 2.50 mmol, 1.00 eq.), NaO^{t}Bu (361 mg, 3.76 mmol, 1.50 eq.), $Pd_2(dba)_3$ (22.9 mg, 25.0 µmol, 0.01 eq.), PCy_3 (35.1 mg, 125 µmol, 0.05 eq.) were dissolved in toluene (10 mL). 2-Bromothioanisole (368 µL, 2.75 mmol, 1.10 eq.) was added and the reaction heated to 100°C for 20 h. After cooling to room temperature, the reaction was diluted with EtOAc (30 mL) and washed with water (2 × 30 mL) and brine (2 × 30 mL). After drying of the organic phase over Na_2SO_4, the solvent was evaporated and the crude product was purified *via* column chromatography (Toluene / Petrolether (40 – 60): 1 / 3). N^1-benzyl-N^2-(2-(methylthio)phenyl)-N^1-phenylbenzene-1,2-diamine (877 mg, 2.21 mmol, 88 %) was obtained as a colourless, highly viscous oil. R_f (Toluene / Petrolether (40 – 60): 1/3): 0.25; NMR: ^{1}H (500.0 MHz, C_6D_6, 25°C) δ (ppm) = 7.37 – 7.35 (m, 1H), 7.30 – 7.29 (m, 1H), 7.27 – 7.24 (m, 3H), 7.18 – 7.16 (m, 1H), 7.07 – 6.94 (m, 8H), 6.79 – 6.76 (m, 3H), 6.72 – 6.67 (m,

2H), 4.66 (s, 2H, NCH_2), 1.74 (s, 3H, SCH_3); $^{13}C\{^1H\}$ (125.8 MHz, C_6D_6, 25°C) δ (ppm) = 148.7, 143.2, 140.6, 139.1, 137.0, 133.1, 129.9, 129.6, 128.7, 128.4, 127.5, 127.4, 127.1, 126.4, 122.1, 121.4, 118.9, 118.1, 116.3, 114.7, 56.4 (s, 1C, NCH_2), 17.3 (s, 1C, SCH_3); IR: (ATR, neat, cm^{-1}) ν = 3325 (br, m), 3064 (m), 3026 (m), 2921 (m), 1581 (s), 1513 (s), 1495 (s), 1313 (m), 744 (s), 692 (m); ESI-MS (MeOH/DCM): 397.2 ($[M+H]^+$).

*Synthesis of **1***

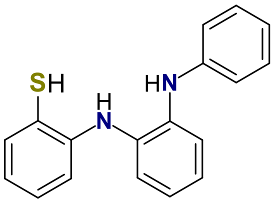

1

N[1]-benzyl-*N*[2]-(2-(methylthio)phenyl)-*N*[1]-phenylbenzene-1,2-diamine (1.70 g, 4.29 mmol, 1.00 eq.) was suspended in NH_3 (40 mL) at –80° and sodium was added in small portions (ca. 10 – 20 mg). The consumption of sodium was indicated by a clear color change from dark blue to green and finally bright yellow. Sodium was added until a dark green or deep blue color persisted for 30 minutes. The cooling bath was removed and the reaction was warmed to –33°C to remove ammonia. At this stage, NH_4Cl was added until the solution decolorized, after which the solvent was removed. Water (25 mL) was added to the obtained off-white powder. HCl (12 M) was added dropwise at 0°C until pH ~4-6. The aqueous phase is extracted with Et_2O (3 × 20 mL) and the combined organic phases were washed with water (2 × 50 mL) and brine (2 × 50 mL) and dried over Na_2SO_4. The solvent was evaporated and the resulting yellow oil dissolved in a minimal amount of toluene and filtered over cotton wool. After evaporation of the solvent, **1** (537 mg, 1.84 mmol, 91 %) was obtained as a highly viscous yellow oil. NMR: ^1H (500.0 MHz, C_6D_6, 25°C) δ (ppm) = 7.28 –7.26 (m, 1H), 7.19 – 7.15 (m, 1H), 7.06 – 7.02 (m, 3H), 6.91 – 6.83 (m, 4H), 6.79 – 6.76 (m, 1H), 6.72 – 6.70 (m, 2H), 6.60 – 6.57 (m, 1H), 6.06 (br, 1H, N*H*), 5.22 (br, 1H, N*H*), 2.65 (m, 1H, S*H*); $^{13}C\{^1H\}$ (125.8 MHz, C_6D_6, 25°C) δ (ppm) = 144.7, 144.2, 136.2, 134.6, 134.5, 129.6, 128.9, 124.1, 123.3, 122.1, 121.1, 121.0, 120.8, 117.8, 117.3, 116.4; IR: (ATR, neat, cm^{-1}) ν = 3355 (br, m), 3045 (w), 1593 (s), 1583 (s), 1515 (s), 1494 (m), 1449 (s), 1476 (m), 1304 (br, m), 742 (s); ESI-MS (EtOAc): 293.0 ($[M+H]^+$).

*Synthesis of **2***

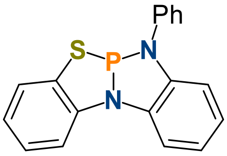

2

1 (135 mg, 462 µmol, 1.00 eq.) was dissolved in THF (10 mL), PCl_3 in THF (806 µL, 462 µmol, 0.573 M, 1.00 eq.) and NEt_3 (212 µL, 1.52 mmol, 3.30 eq.) were added and the solution heated to 50°C for 5 hours. After cooling to room temperature, the solvent was removed and the residue is extracted with benzene (2 × 5 mL). Lyophilization out of benzene gives P(NNS) (142 mg, 443 µmol, 96 %) as an off-white powder. The material obtained *via* this route is pure enough for further synthesis but caries a small impurity of triethylamine hydrochloride. To obtain spectroscopically pure material the compound can be washed with pentane (2 × 10 mL) and extracted with a minimal amount of benzene (ca. 2 mL). Lyophilization yields **2** as an off-white powder, accompanied by a reduced overall yield (70 %). Single crystals suitable for X-ray crystallography were obtained by storing a concentrated

toluene solution of **2** at –35°C for 3 weeks. The $^{31}P\{^1H\}$ spectra usually feature an additional peak corresponding to a dimeric species, which reacts cleanly with all studied substrates (see Figure S11). NMR: ^1H (400.0 MHz, C_6D_6, 25°C) δ (ppm) = 7.34 – 7.30 (m, 2H), 7.12 – 7.10 (m, 2H), 7.02 – 6.97 (m, 3H), 6.95 – 6.91 (m, 1H), 6.88 – 6.74 (m, 5H); $^{13}C\{^1H\}$ (100.6 MHz, C_6D_6, 25°C) δ (ppm) = 142.7 (s, 1C); 141.5 (d, $J_{C–P}$ = 10.0 Hz, 1C); 138.5 (d, $J_{C–P}$ = 14.8 Hz, 1C); 136.9 (d, $J_{C–P}$ = 6.5 Hz, 1C); 136.6 (d, $J_{C–P}$ = 11.0 Hz, 1C); 130.1 (s, 1C); 128.6 (s, 1C); 127.6 (s, 1C); 126.9 (s, 1C); 125.4 (d, $J_{C–P}$ = 8.2 Hz, 1C); 125.0 (s, 1C); 124.6 (d, $J_{C–P}$ = 9.8 Hz, 1C); 122.9 (s, 1C); 122.4 (s, 1C); 114.2 (d, $J_{C–P}$ = 1.7 Hz, 1C); 112.1 (s, 1C); $^{31}P\{^1H\}$ (162.0 MHz, $CDCl_3$, 25°C) δ (ppm) = 165.9 (s, 1P); ESI-MS ($CDCl_3$): 321.0 ($[M+H]^+$).

*Synthesis of **3***

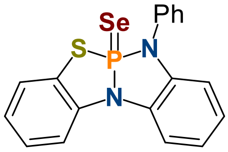

3

2 (43.0 mg, 139 µmol, 1.00 eq.) and Se powder (53.0 mg, 671 µmol, 5.00 eq.) were suspended in $CDCl_3$ (0.5 mL) in a J-Young NMR tube and stirred until the $^{31}P\{^1H\}$ NMR spectrum indicated full conversion (5 d). The reaction mixture was filtered and the solvent was removed. The residue was redissolved in THF (0.5 mL), layered with pentane (3 mL) and stored at –35°C for 3 d. The solution was filtered cold, washed with pentane (2 × 1 mL), extracted with THF (2 × 0.5 mL) and dried. **3** is obtained as a colorless solid (40.0 mg, 100 µmol, 75%). Single crystals suitable for X-ray crystallography were obtained by slow diffusion of pentane into a saturated THF solution at –35°C. NMR: ^1H (500.0 MHz, $CDCl_3$, 25°C) δ (ppm) = 7.53 – 7.21 (m, 1H), 7.34 – 7.29 (m, 2H), 7.27 – 7.22 (m, 2H), 7.17 – 7.14 (m, 1H), 7.12 – 7.08 (m, 1H), 7.05 – 6.99 (m, 2H), 6.72 – 6.64(m, 2H), 6.34 – 6.31 (m, 2H); $^{13}C\{^1H\}$ (125.7 MHz, $CDCl_3$, 25°C) δ (ppm) = 137.6 (d, $J_{C–P}$ = 10.8 Hz, 1C), 137.3 (d, $J_{C–P}$ = 13.5 Hz, 1C), 136.1 (d, $J_{C–P}$ = 5.5 Hz, 1C), 135.0 (d, $J_{C–P}$ = 5.3 Hz, 1C), 130.5 (d, $J_{C–P}$ = 4.9 Hz, 1C), 130.3 (d, $J_{C–P}$ = 0.9 Hz, 1C), 128.9 (s, 1C), 128.8 (s, 1C), 127.5 (s, 1C), 126.7 (s, 1C), 125.3 (d, $J_{C–P}$ = 8.8 Hz, 1C), 123.8 (s, 1C), 123.1 (d, $J_{C–P}$ = 14.1 Hz, 1C), 121.8 (s, 1C), 113.0 (d, $J_{C–P}$ = 8.3 Hz, 1C), 111.0 (d, $J_{C–P}$ = 6.3 Hz, 1C); $^{31}P\{^1H\}$ (162.0 MHz, $CDCl_3$, 25°C) δ (ppm) = 79.8 ($^1J_{P–Se}$ = 923.8 Hz, 1P); $^{77}Se\{^1H\}$ (76.4 MHz, $CDCl_3$, 25°C) δ (ppm) = 173.9 (d, $^1J_{Se–P}$ = 923.8 MHz, 1 Se). No product peak detected in ESI-MS (toluene, THF, $CDCl_3$).

*Reaction of **2** with $B(C_6F_5)_3$ to generate **4***

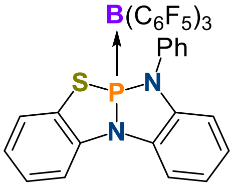

4

Reaction in THF: **2** (15.0 mg, 46.8 µmol, 1.00 eq.) and $B(C_6F_5)_3$ (24.0 mg, 46.8 µmol, 1.00 eq.) were dissolved in THF (0.5 mL) and stirred for 16 h. The $^{31}P\{^1H\}$, $^{11}B\{^1H\}$ and $^{19}F\{^1H\}$ NMR spectra did not indicate any conversion. Reaction in C_6D_6: **2** (15.0 mg, 46.8 µmol, 1.00 eq.) and $B(C_6F_5)_3$ (24.0 mg, 46.8 µmol, 1.00 eq. or 12.0 mg, 23.4 µmol, 0.50 eq.) were dissolved in C_6D_6 (0.5 mL), stirred for 16 h and analyzed by NMR spectroscopy. Single crystals suitable for X-ray crystallography were obtained by slow diffusion of hexane into the reaction mixture at room temperature. 1.0 equivalent: ^1H (400 MHz, C_6D_6, 25°C) δ (ppm) = 7.31 – 7.29 (m, 2H), 7.18 – 7.13 (m, 4H), 7.03 – 6.99 (m, 3H), 6.94 – 6.91 (m, 1H), 6.88 – 6.82 (m, 2H), 6.69 - 6.65 (m, 1H), 6.61 – 6.58 (m, 1H); $^{31}P\{^1H\}$ (162 MHz, C_6D_6, 25°C)

δ (ppm) = 175.0 (s, 1P); $^{11}B\{^1H\}$ (128.4 MHz, C_6D_6, 25°C) δ (ppm) = 10.3 (br, 1B); $^{19}F\{^1H\}$ (376.5 MHz, C_6D_6, 25°C) δ (ppm) = –129.3 (d, $^3J_{FF}$ = 23.8 Hz, 2F), –153.2 (t, $^3J_{FF}$ = 21.1 Hz, 1F), –162.7 to –162.9 (m, 2F); 0.5 equivalents: ^1H (400 MHz, C_6D_6, 25°C) δ (ppm) = 7.37 – 7.32 (m, 3H), 7.17 – 7.09 (m, 5H), 6.99 – 6.94 (m, 1H), 6.93 – 6.88 (m, 2H), 6.85 – 6.81 (m, 2H); $^{31}P\{^1H\}$ (162 MHz, C_6D_6, 25°C) δ (ppm) = 169.6 (s, 1P); $^{11}B\{^1H\}$ (128.4 MHz, C_6D_6, 25°C) δ (ppm) = 4.0 (br, 1B); $^{19}F\{^1H\}$ (376.5 MHz, C_6D_6, 25°C) δ (ppm) = –129.3 (br, 2F), –154.5 (br, 1F), –162.9 to –163.1 (m, 2F).

*Reaction of **2** with para-anisidine to generate **5a/b***

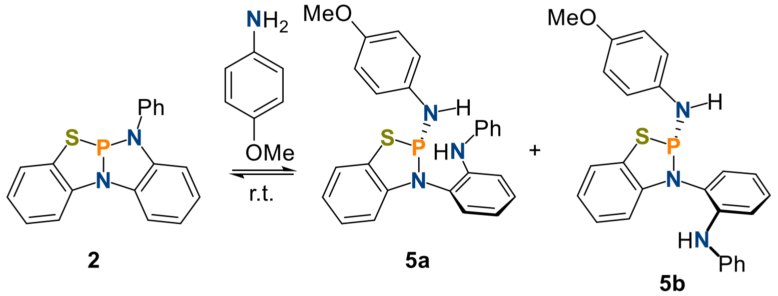

2 (5.0 mg, 16 µmol, 1.0 eq.) and *para*-anisidine (9.6 mg, 78 µmol, 10 eq.) were dissolved in $CDCl_3$ (0.5 mL) in a J-Young NMR tube and stirred for 16 h. The solution was analyzed by (VT)-NMR. The proton resonances were assigned based on ^{31}P–^1H 2D NMR experiments. ^1H (500 MHz, $CDCl_3$, 25°C) δ (ppm) = 7.64 - 7.62 (m, 1H, **2**), 7.56 – 7.51 (m, 2H, **2**), 7.47 – 7.42 (aromatic signals of both isomers), 7.30 – 6.82 (aromatic signals of both isomers), 6.53 – 6.48 (aromatic signals of both isomers), 6.46 (br, 1H, N*H*, **5a**), 5.76 (br, 1H, N*H*, **5b**), 5.34 (d, $^2J_{HP}$ = 16.0 Hz, 2H, PN*H*2, **5a**), 5.31 (d, $^2J_{HP}$ = 11.0 Hz, 2H, PN*H*2, **5b**); $^{31}P\{^1H\}$ (202 MHz, $CDCl_3$, 25°C) δ (ppm) = 165.9 (s, 1P, **2**), 88.4 (d, $^2J_{HP}$ = 16.0 Hz, 1P, **5a**), 83.5 (d, $^2J_{HP}$ = 11 Hz, 1P, **5b**).). No product peak detected in ESI-MS (toluene, THF, $CDCl_3$).

*Reaction of **2** with ammonia to generate **6a/b***

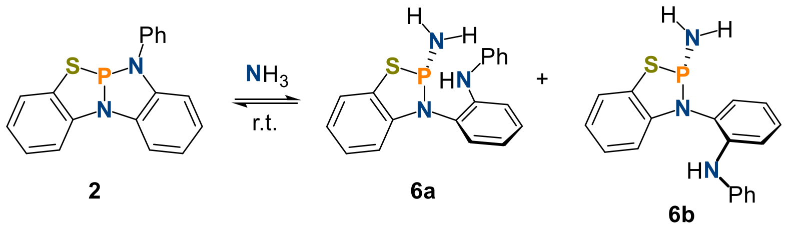

2 (5.0 mg, 16 µmol, 1.0 eq.) was dissolved in THF(-d8) (0.5 mL) in a J-Young NMR tube and degassed by two pump-freeze-thaw cycles. Dry gaseous ammonia was introduced to the tube at room temperature, the tube is shaken for 2 minutes and analyzed by NMR spectroscopy. No changes in the proton or phosphorus NMR spectra were observed when the reaction is carried out in the presence of $B(C_6F_5)_3$. ^1H (500 MHz, THF-d8, 25°C) δ (ppm) = 7.44 – 7.36 (m, 2H), 7.34 (br, 1H, N*H*, **6a**), 7.32 – 7.29 (m, 2H), 7.23 -7.15 (m, 3H), 7.11 – 7.09 (m, 1H), 7.04 – 6.85 (m, 3H), 6.79 – 6.79 (m, 1H), 6.37 – 6.34 (m, 1H), 6.30 (br, 1H, N*H*, **6b**), 4.57 (d, $^2J_{HP}$ = 15.2 Hz, 2H, N*H*2, **6a**), 4.31 (d, $^2J_{HP}$ = 14.8 Hz, 2H, N*H*2, **6b**); $^{13}C\{^1H\}$ (125.7 MHz, THF-d8, 25°C) δ (ppm) = 146.0 (d, J_{HP} = 12.9 Hz, 1C), 145.5 (d, J_{HP} = 10.2 Hz, 1C), 144.3 (d, J_{HP} = 1.8 Hz, 1C), 143.8 (1C), 143.6 (1C), 143.5 (d, J_{HP} = 4.0 Hz, 1C), 133.0 (d, J_{HP} = 6.0 Hz, 1C), 132.5 (d, J_{HP} = 3.5 Hz, 1C), 129.3 (d, J_{HP} = 2 Hz, 1C), 129.9 (d, J_{HP} = 12.0 Hz, 2C), 129.3 (1C), 129.2 (1C), 129.1 (d, J_{HP} = 6 Hz, 1C), 129.0 (1C), 126.3 (d, J_{HP} = 3.0 Hz, 1C), 125.9 (d, J_{HP} = 4.0 Hz, 1C), 125.8 (d, J_{HP} = 6.0 Hz, 1C), 125.8 (1C), 125.7 (1C), 125.6 (d, J_{HP} = 4.0 Hz, 1C), 122.6 (1C), 122.3 (1C), 120.9 (2C), 120.6 (d, J_{HP} =132 Hz, 1C) 120.4 (d, J_{HP} = 7.3 Hz, 1C), 120.3 (1C), 116.6 (2C), 113.6 (d, J_{HP} = 2.4 Hz, 1C), 112.7 (d, J_{HP} = 2.1 Hz, 1C); $^{31}P\{^1H\}$ (202 MHz, THF-d8, 25°C) δ (ppm) = 92.1 (t, $^2J_{HP}$ = 15.2 Hz, 1P, **5a**),

83.5 (t, $^2J_{HP}$ = 14.8 Hz, 1P, **5b**); IR: (ATR, neat, cm^{-1}) ν = 3398 (br, m), 3320 (br, m), 3054 (br,m), 1592 (s), 1509 (m), 1494 (m), 1458 (s), 1310 (m), 1281 (m), 742 (s). VT-NMR under argon atmosphere: After preparing **6** in THF, the solvent is removed and the sample was redissolved in THF-d$_8$ (0.5 mL) and analyzed by variable temperature NMR. NMR under vacuum: After preparing **6** in THF, the solvent was removed and the sample redissolved in THF-d$_8$ (0.5 mL), degassed *via* three pump-freeze-thaw cycles and heated to 60°C for 1h. NMR spectroscopy indicates full regeneration of **2**. Reaction of **6** with $B(C_6F_5)_3$: After preparing **6** in THF, the solvent was removed, one equivalent of $B(C_6F_5)_3$ was added and the mixture redissolved in THF (0.5 mL). After stirring for 16 h the sample was analyzed by NMR spectroscopy. Formation of an ammonia BCF adduct was confirmed by boron and fluorine NMR spectroscopy.[3] ^{31}P{^{1}H} (162 MHz, THF, 25°C) δ (ppm) = 165.5 (s, 1P, **2**), 139.7 (s, 2P, dimeric **2**); ^{11}B{^{1}H} (128.4 MHz, THF, 25°C) δ (ppm) = –10.7 (br, 1B); ^{19}F{^{1}H} (376.5 MHz, THF, 25°C) δ (ppm) = –134.5 to – 134.6 (m, 2F), –159.2 (t, $^3J_{FF}$ = 20.4 Hz, 1F), –165.4 - –165.5 (m, 2F).). No product peak detected in ESI-MS (toluene, THF, CDCl$_3$).

*Synthesis of **7a/b***

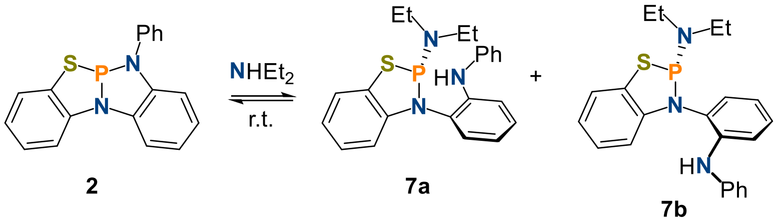

Route A: **2** (10.0 mg, 125 µmol, 10.0 eq.) is dissolved in THF (5 mL) and diethylamine (129 µL, 1.25 mmol, 10.0 eq.) and stirred for 16 h at room temperature. The solvent is removed and the residue is extracted with pentane (3 x 10 mL). Lyophilization out of benzene yield **7** as a colorless powder as a white solid (20.0 mg, 50.8 µmol, 41%), with small amounts of **2** being regenerated upon drying (ca 1%). *The product is poorly soluble in pentane, however ^{31}P{^{1}H} NMR of the reaction mixture shows quantitative conversion to the desired product with minor side products (under 5%) which are not soluble in pentane. Route B:* **2** (10.0 mg, 31.2 µmol, 1.00 eq.) was dissolved in diethylamine (0.5 mL, 4.83 mmol, 155 eq.) and stirred at room temperature in a J-Young NMR tube for 3 h. The solvent was removed and the product dried under vacuum for 48 h. **7** is obtained almost quantitatively as a white solid, with small amounts of **2** being regenerated upon drying (up to 9%). ^{1}H (400 MHz, THF-d$_8$, 25°C) δ (ppm) = 7.40 – 7.34 (m, **7a/b**), 7.26 – 7.23 (m, **7a/b**), 7.12 – 7.04 (m, **7a/b**, 7.01 – 6.93 (m, **7a/b**), 6.86 – 6.81 (m, **7a/b**), 6.79 – 6.65 (m, **7a/b**), 6.58 – 6.55 (m, 1H, **7b**), 6.51 – 6.48 (m, 1H, **7b**), 6.34 (br, 1H, N*H*, **7a**), 5.91 (br, 1H, N*H*, **7b**), 2.92 – 2.75 (m, 2H, NC*H*$_2$, **7a/b**), 2.69 – 2.56 (m, 2H, NC*H*$_2$, **7a/b**), 0.74 (t, $^3J_{HH}$ = 7.1 Hz, 6H, NCH$_2$C*H*$_3$, **7b**), 0.69 (t, $^3J_{HH}$ = 7.1 Hz, 6H, NCH$_2$C*H*$_3$, **7a**); ^{13}C{^{1}H} (125.7 MHz, THF-d$_8$, 25°C) δ (ppm) = 146.2 (J_{CP} = 11.4 Hz), 142.9 (J_{CP} = 2.7 Hz), 142.7, 142.6, 142.1, 132.0 (J_{CP} = 4.1 Hz), 131.1 (J_{CP} = 5.5 Hz), 130.8 (J_{CP} = 13.0 Hz), 129.8, 129.6, 129.5, 128.9 (J_{CP} = 2.7 Hz), 128.7 (J_{CP} = 2.7 Hz), 127.3, 127.2, 125.8 (J_{CP} = 4.1 Hz), 128.6, 125.2 (J_{CP} = 4.5 Hz), 125.1, 122.6, 122.3, 121.6 (J_{CP} = 2.3 Hz), 120.8, 120.7, 119.88 (J_{CP} = 2.3 Hz), 119.7, 116.1, 115.9 (J_{CP} = 2.3 Hz), 112.5 (J_{CP} = 2.7 Hz), 111.3 (J_{CP} = 2.7 Hz), 41.3 (d ,$^2J_{CP}$ = 19.6 Hz, 2C, N*C*H$_2$CH$_3$, **7a**), 41.1 (d ,$^2J_{CP}$ = 16.7 Hz, 2C, N*C*H$_2$CH$_3$, **7b**), 14.7 (d, $^3J_{CP}$ = 3.6 Hz, 2C, NCH$_2$*C*H$_3$, **7a**), 14.5 (d, $^3J_{CP}$ = 3.6 Hz, 2C, NCH$_2$*C*H$_3$, **7b**); ^{31}P{^{1}H} (162 MHz, THF, 25°C) δ (ppm) = 105.6 (t, $^3J_{HP}$ = 9.4 Hz, 1P, **7a**), 98.2 (t, $^3J_{HP}$ = 9.1 Hz, 1P, **7b**); IR: (ATR, neat, cm^{-1}) ν = 3401 (br, m), 2969 (m), 1592 (s), 1510 (s), 1461 (s), 1310 (m), 1283 (m), 1174 (m), 1016 (m), 740 (s); ESI-MS (CDCl$_3$): 393.0 ([M+H]$^+$).

Analytical data

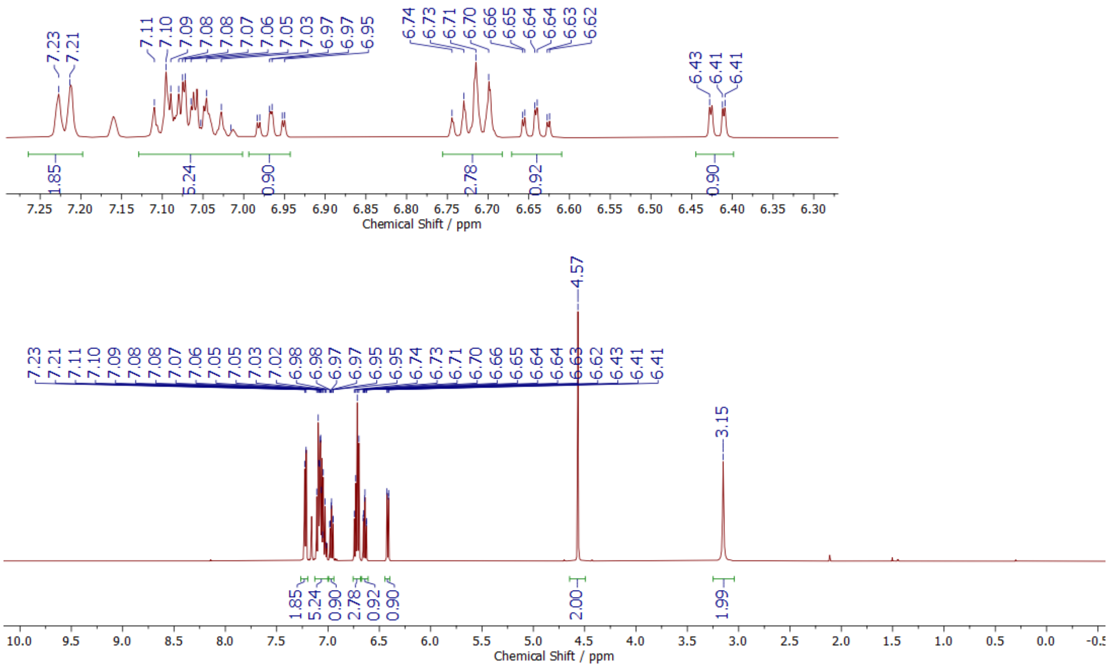

Figure S1. ^{1}H NMR spectrum of **SM1**, C_6D_6, 25°C.

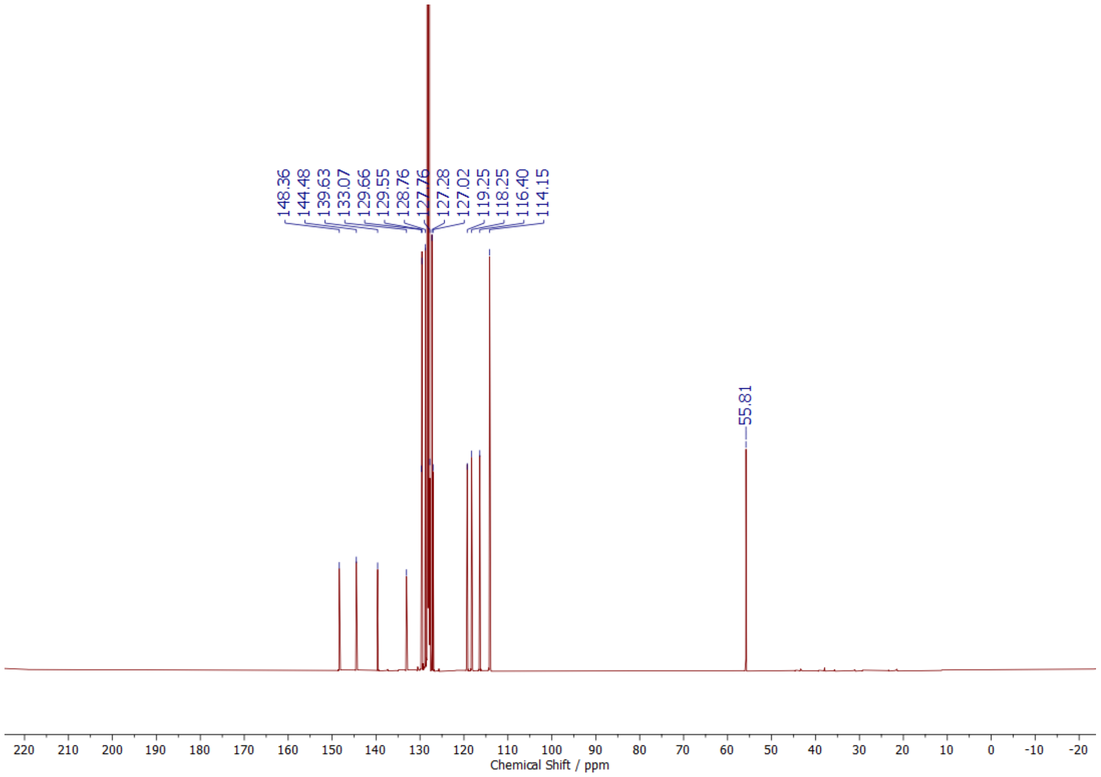

Figure S2. $^{13}C\{^{1}H\}$ NMR spectrum of **SM1**, C_6D_6, 25°C.

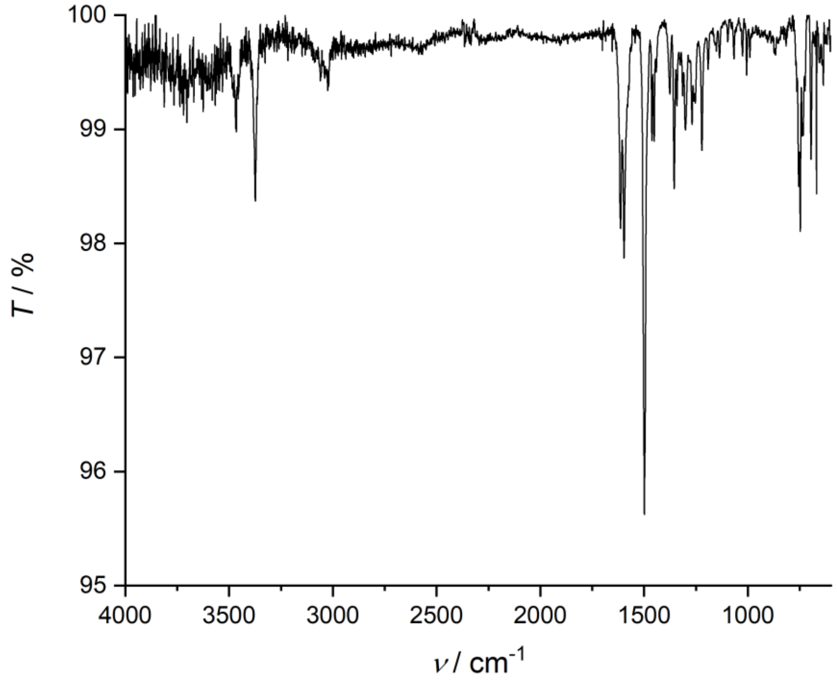

Figure S3. IR spectrum of **SM1**, neat, ATR, 25°C.

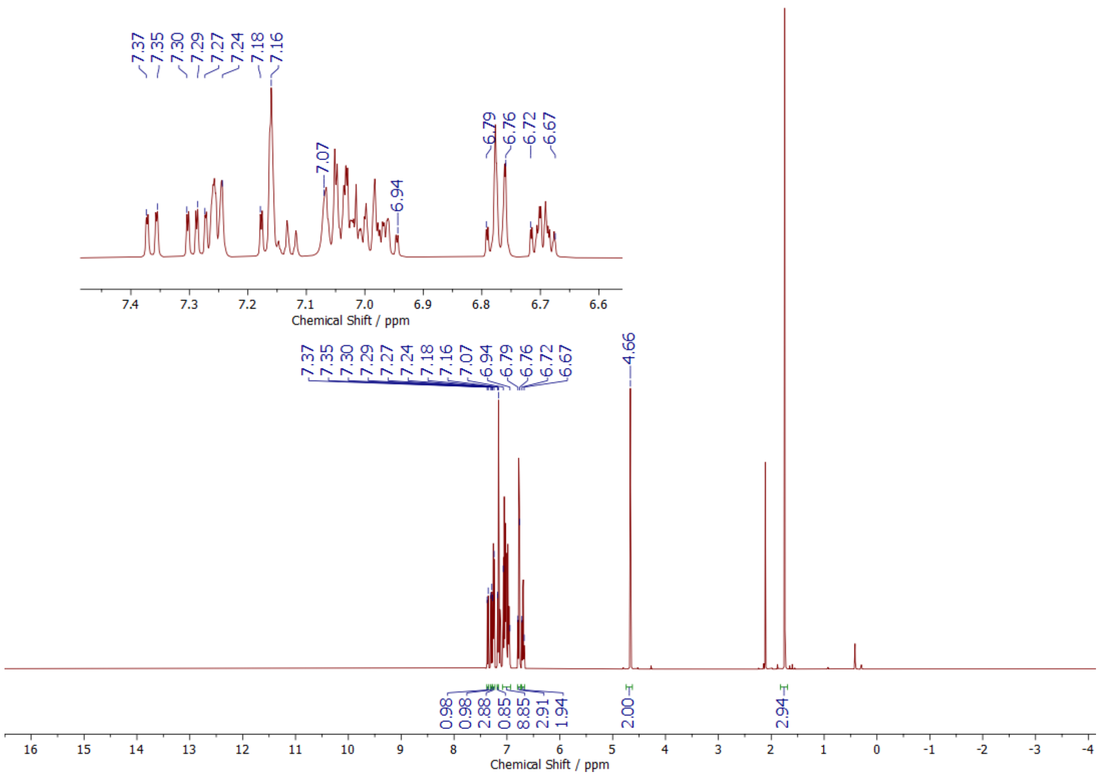

Figure S4. ^{1}H NMR spectrum of **SM2**, C_6D_6, 25°C, residual toluene from the work-up is detected at δ_H = 7.13, 7.02, 2.11 ppm.

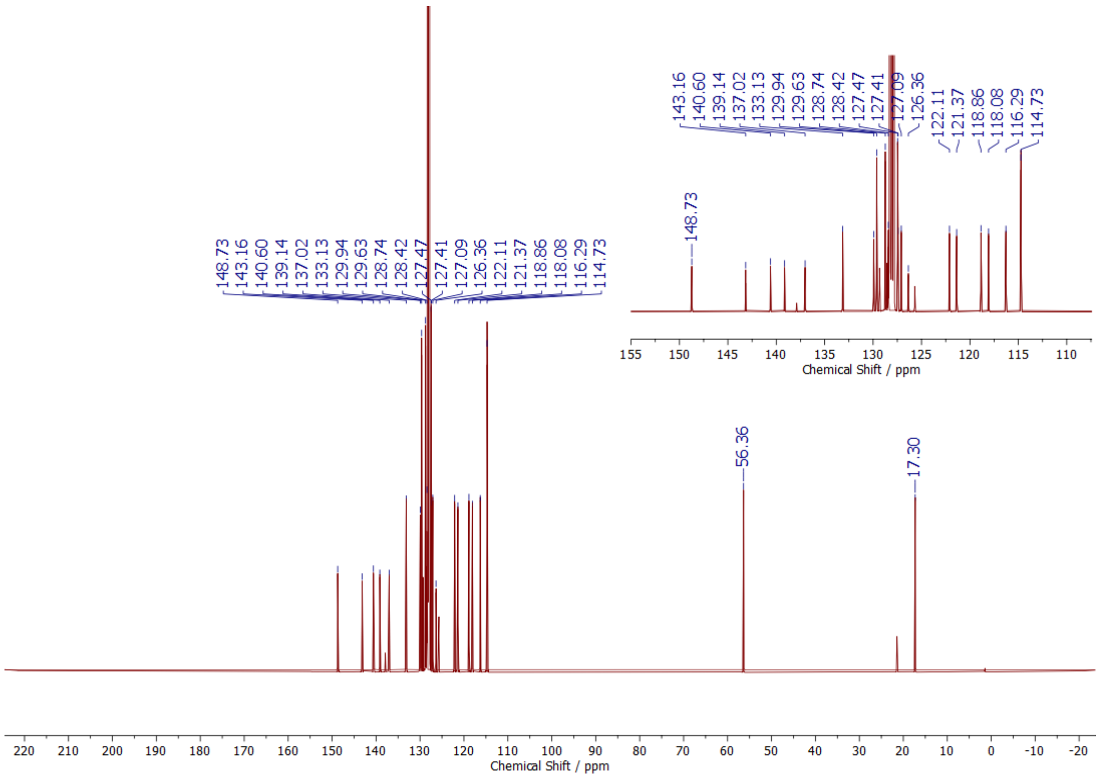

Figure S5. $^{13}C\{^{1}H\}$ NMR spectrum of **SM2**, C_6D_6, 25°C, residual toluene from the work-up is detected at δ_C = 137.9, 129.3, 128.6, 125.7, 21.1 ppm.

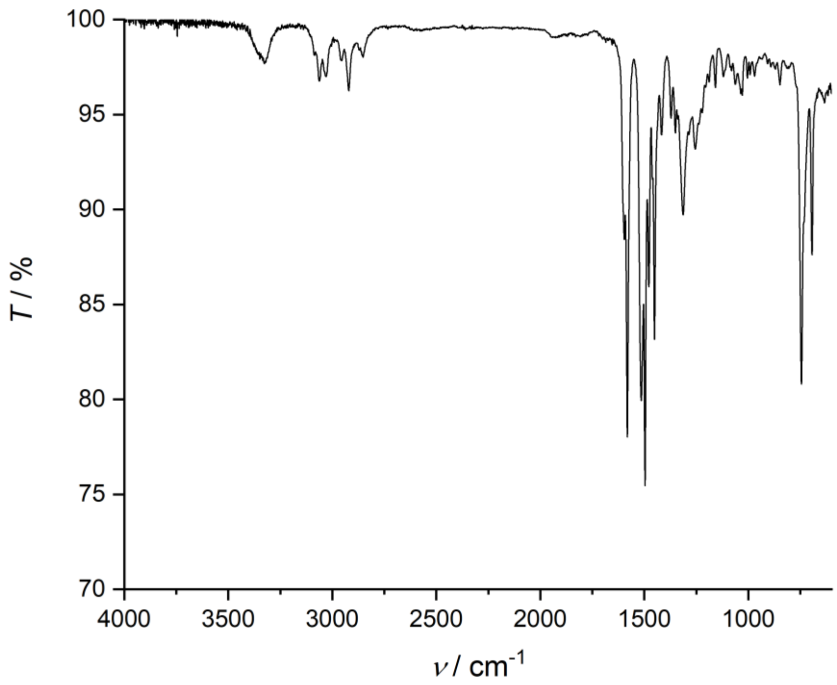

Figure S6. IR spectrum of **SM2**, neat, ATR, 25°C.

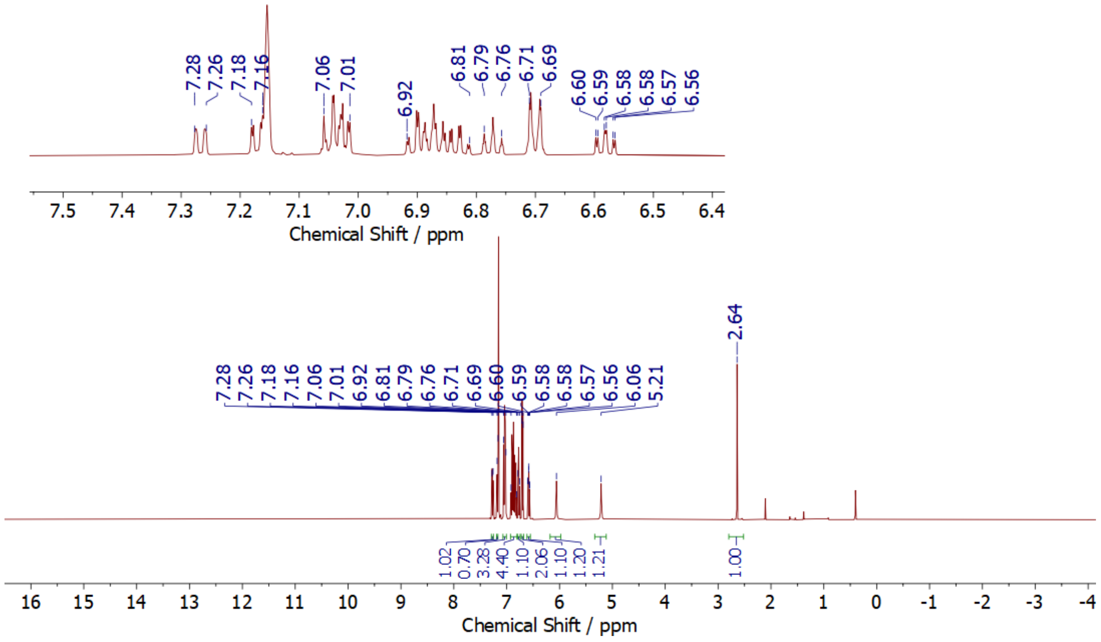

Figure S7. ^{1}H NMR spectrum of **1**, C_6D_6, 25°C, residual toluene from the work-up is detected at δ_H = 7.13, 7.02, 2.11 ppm.

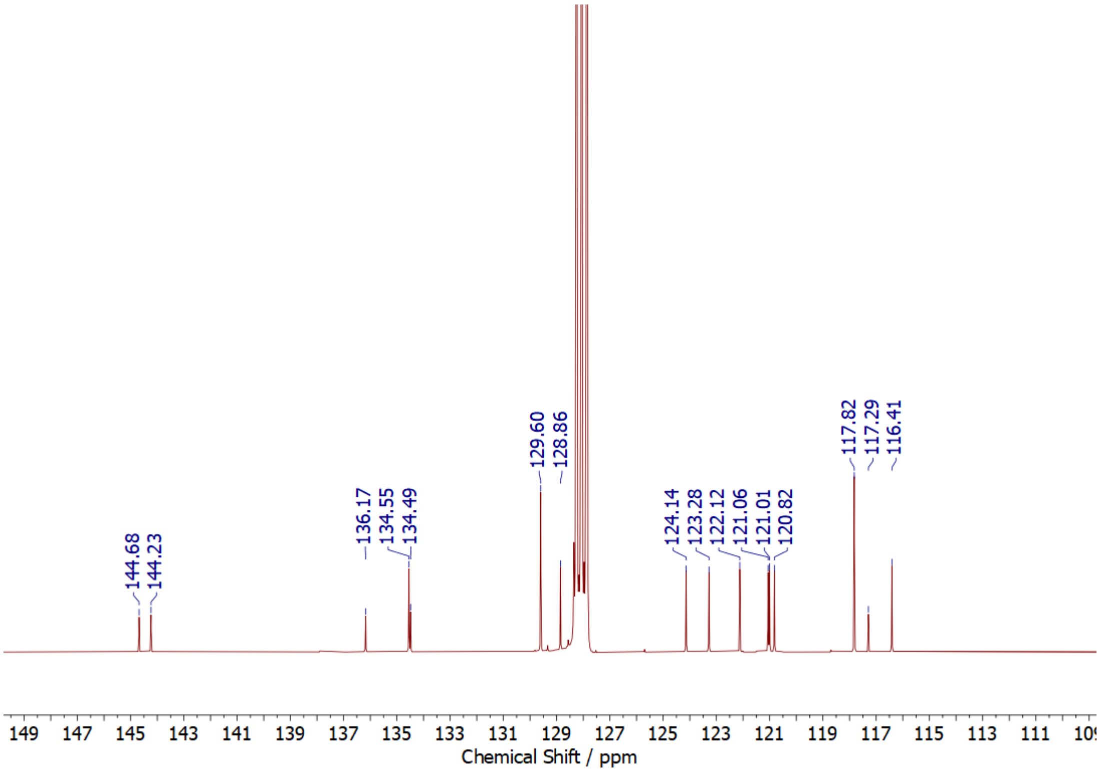

Figure S8. $^{13}C\{^{1}H\}$ NMR spectrum of **1**, C_6D_6, 25°C, residual toluene from the work-up is detected at δ_C = 137.9, 129.3, 128.6, 125.7, 21.1 ppm.

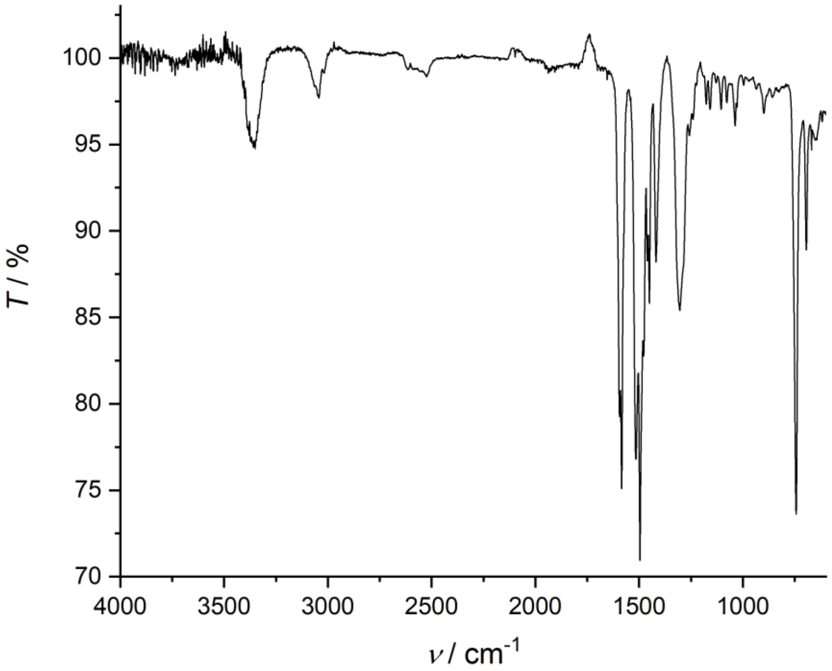

Figure S9. IR spectrum of **1**, neat, ATR, 25°C.

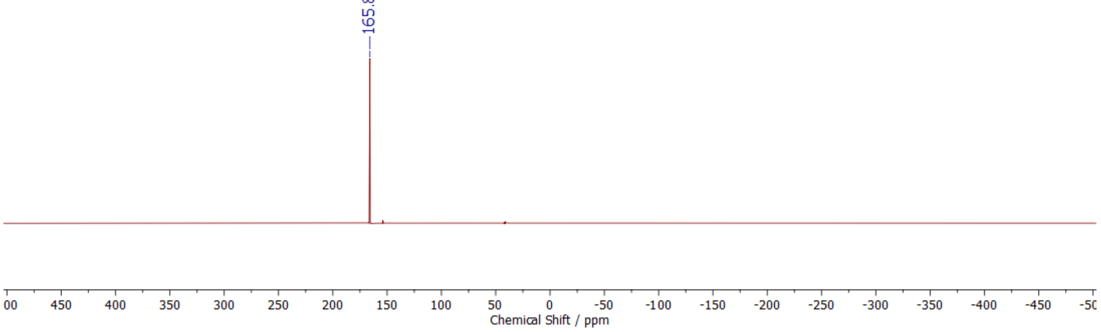

Figure S10. $^{31}P\{^{1}H\}$ NMR spectrum of **2**, C_6D_6, 25°C.

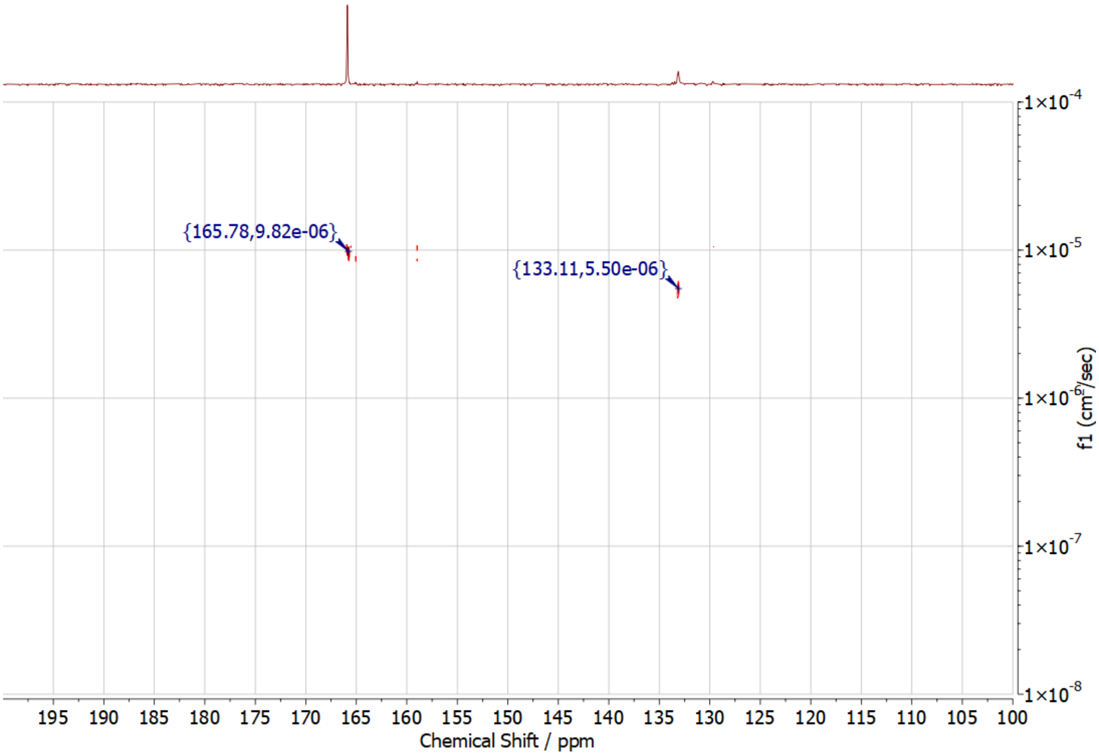

Figure S11. $^{31}P\{^{1}H\}$-DOSY NMR spectrum of **2** showing the formation of a dimeric species, C_6D_6, 25°C. Depending on the synthesis reaction conditions, this peak can have varying integrals When reacted with amines, Se or $B(C_6F_5)_3$ clean conversion towards the desired products (**3** ,**4**, **5a/b**, **6a/b**, **7a/b**) is detected. Relative radii derived from the Stokes-Einstein relation: $r_{\text{Dimer}} = r_{\mathbf{2}}\, D_{\mathbf{2}}/D_{\text{Dimer}} = 1.79{*}r_{\mathbf{2}}$.

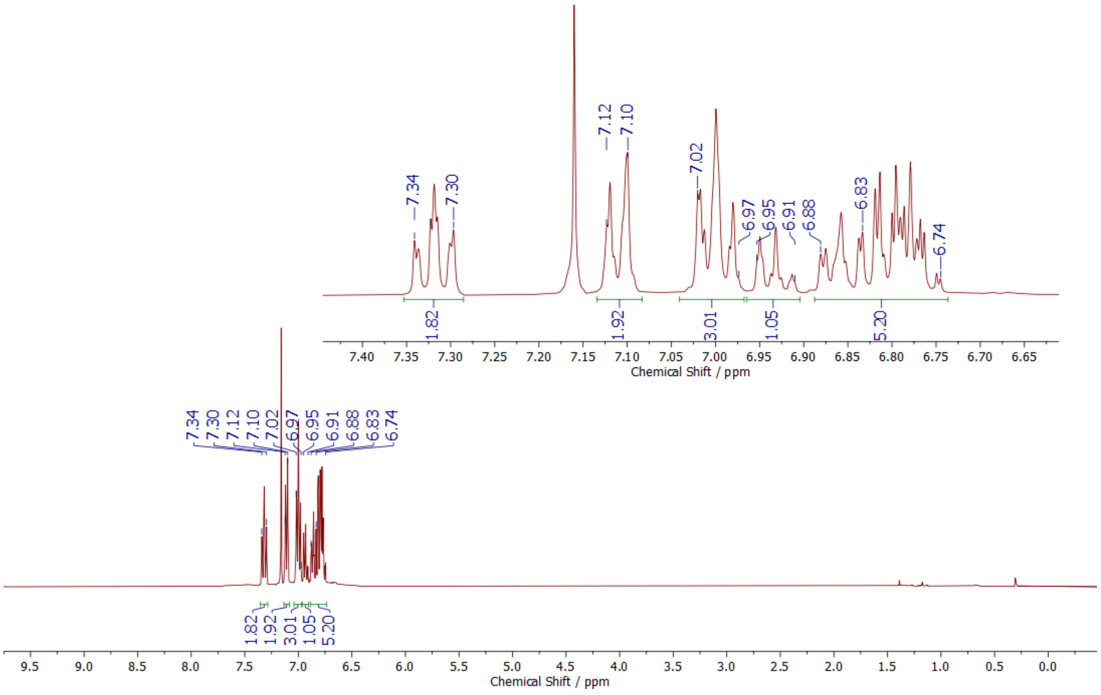

Figure S12. ^{1}H NMR spectrum of **2**, C_6D_6, 25°C.

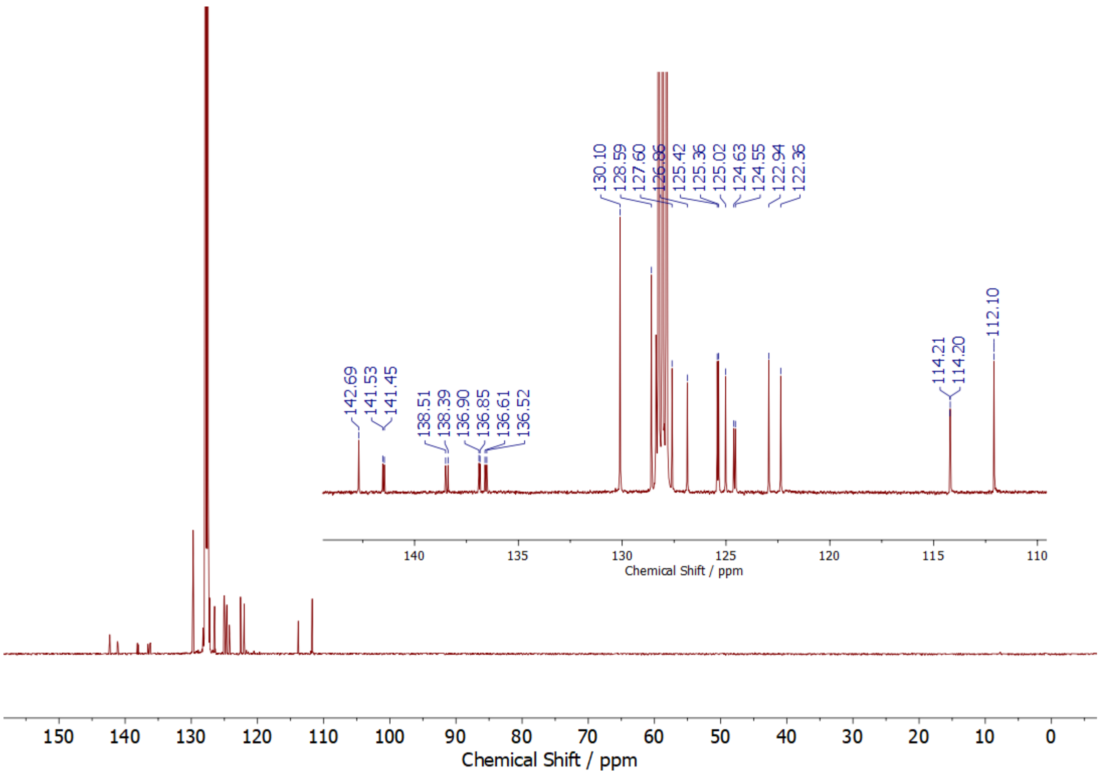

Figure S13. $^{13}C\{^{1}H\}$ NMR spectrum of **2**, C_6D_6, 25°C.

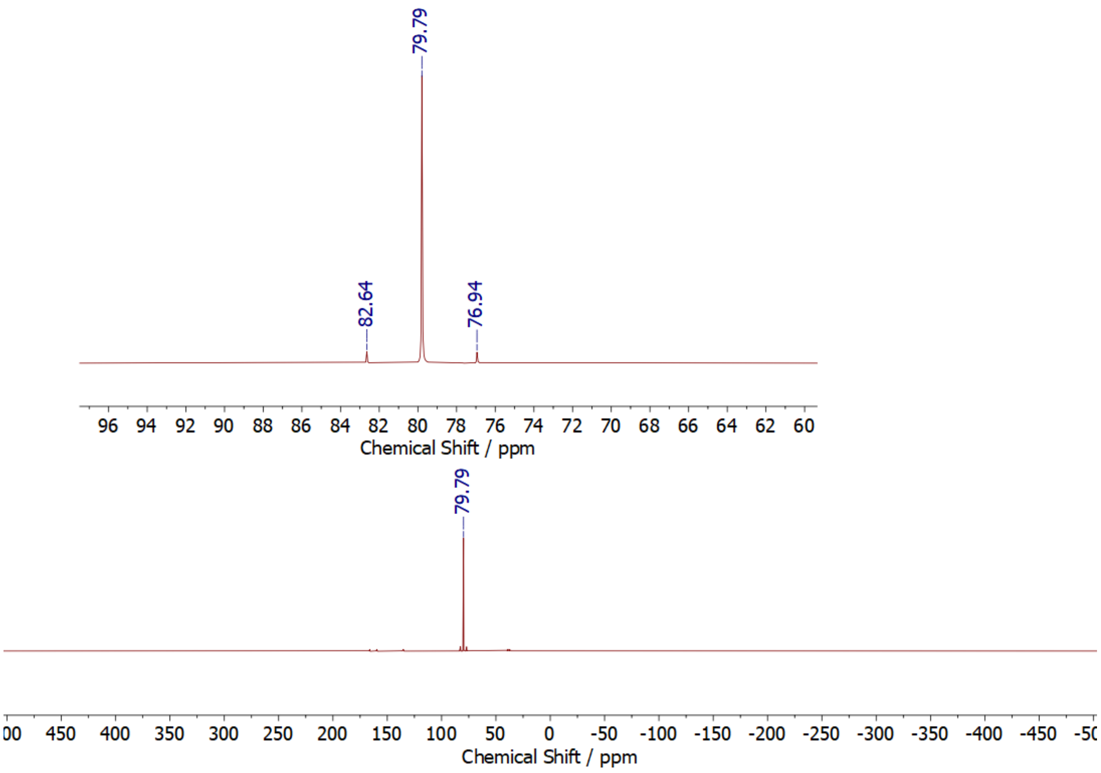

Figure S14. $^{31}P\{^{1}H\}$ NMR spectrum of **3**, $CDCl_3$, 25°C.

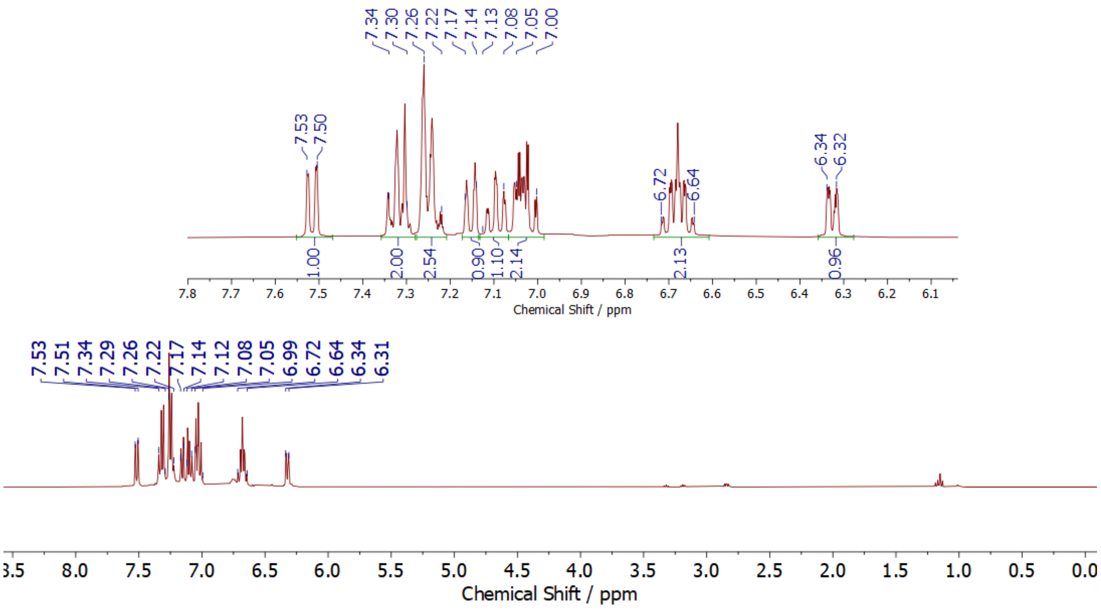

Figure S15. ^{1}H NMR spectrum of **3**, $CDCl_3$, 25°C.

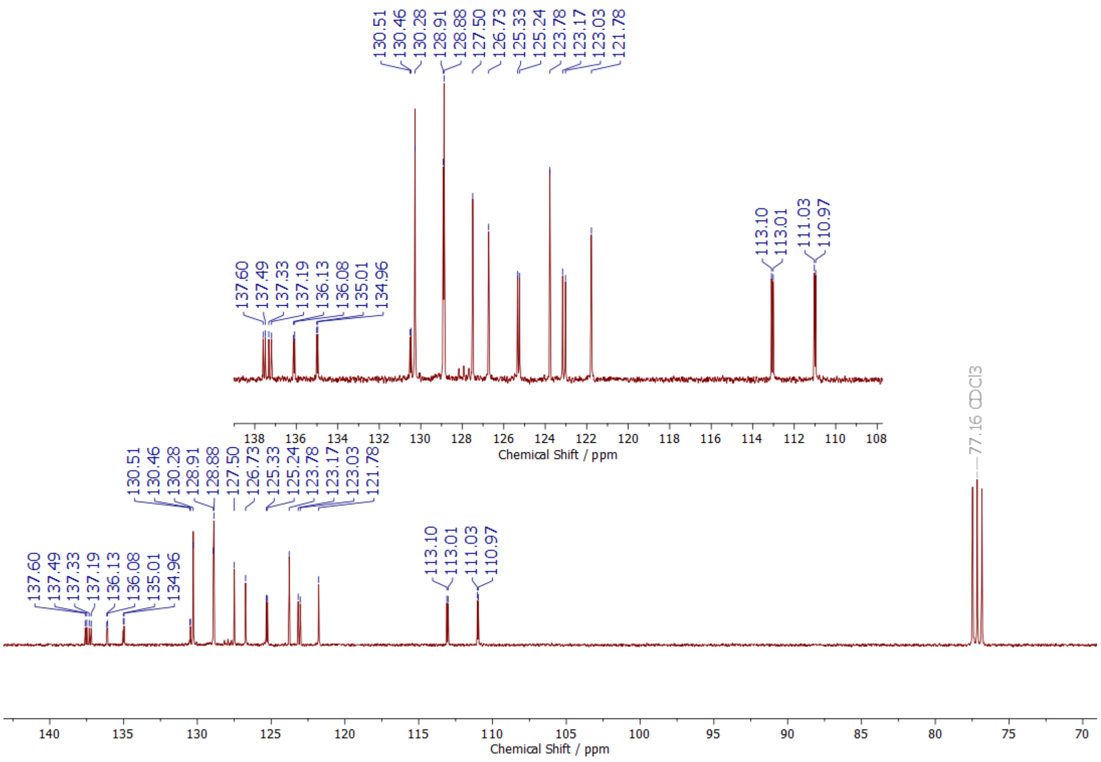

Figure S16. $^{13}C\{^{1}H\}$ NMR spectrum of **3**, $CDCl_3$, 25°C.

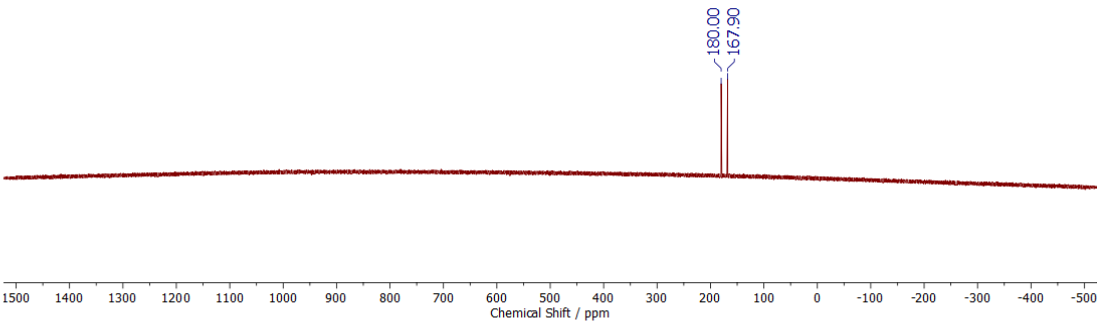

Figure S17. $^{77}Se\{^1H\}$ NMR spectrum of **3**, $CDCl_3$, 25°C.

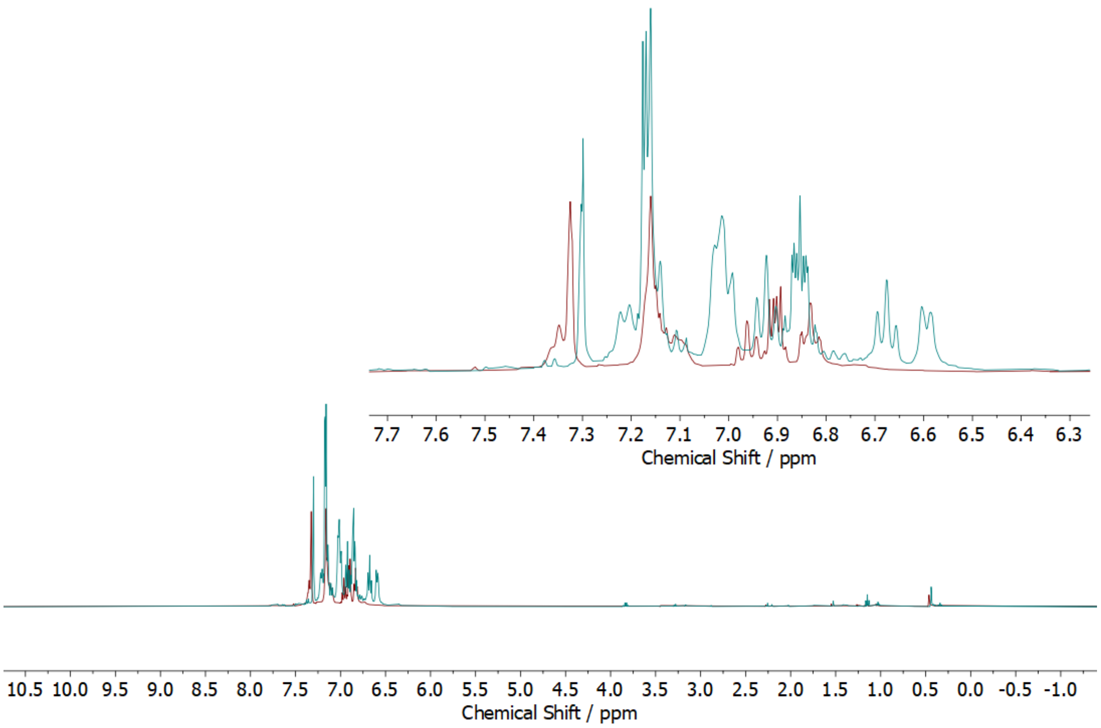

Figure S18. ^1H NMR spectra of the reaction of **2** with $B(C_6F_5)_3$ to generate **4** (0.5 eq. {red} and 1.0 eq. {turquoise}), C_6D_6, 25°C.

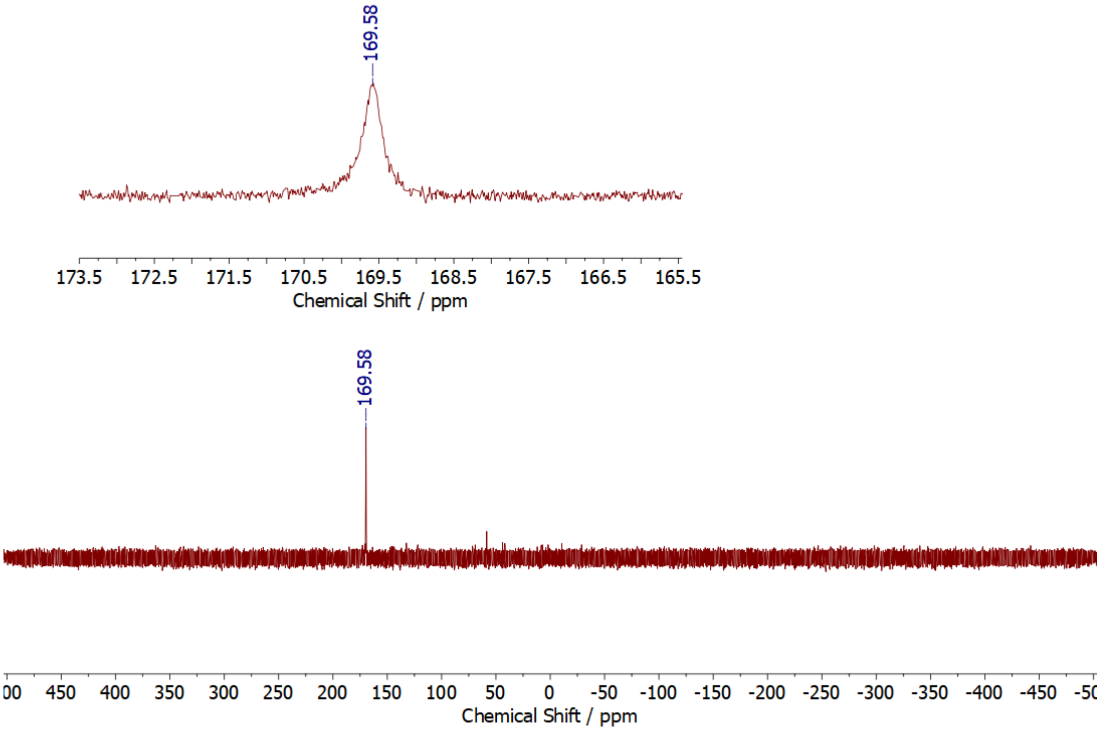

Figure S19. $^{31}P\{^{1}H\}$ NMR spectrum of the reaction of **2** with 0.5 eq. $B(C_6F_5)_3$ to generate **4**, C_6D_6, 25°C.

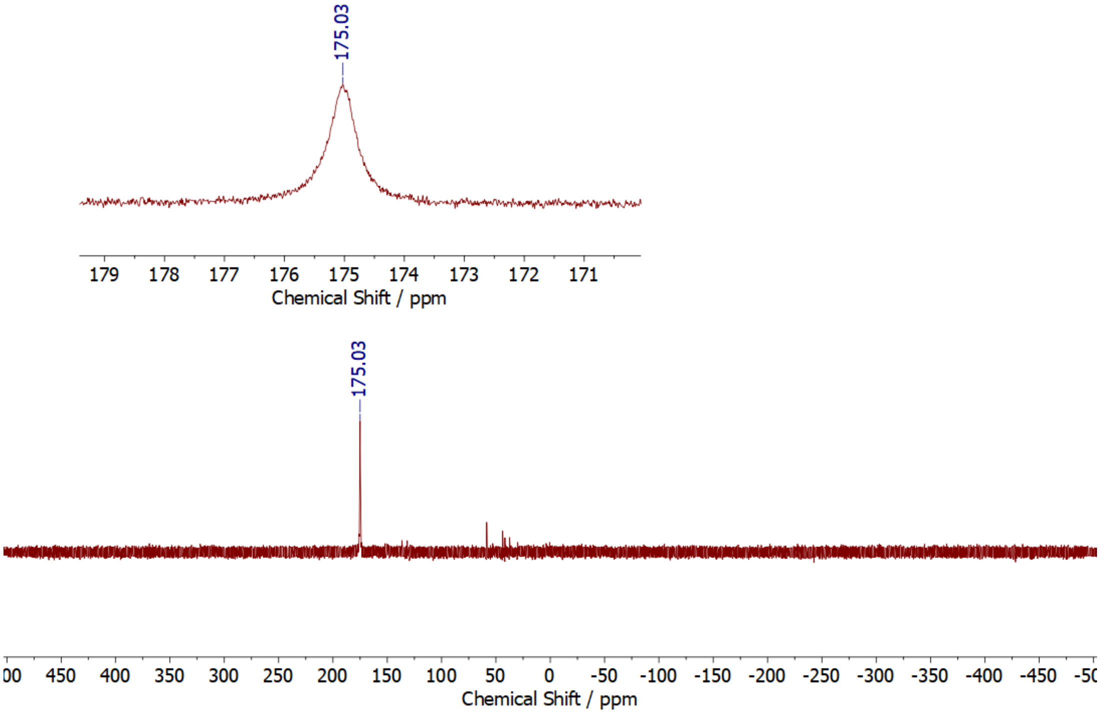

Figure S20. $^{31}P\{^{1}H\}$ NMR spectrum of the reaction of **2** with 1.0 eq. $B(C_6F_5)_3$ to generate **4**, C_6D_6, 25°C.

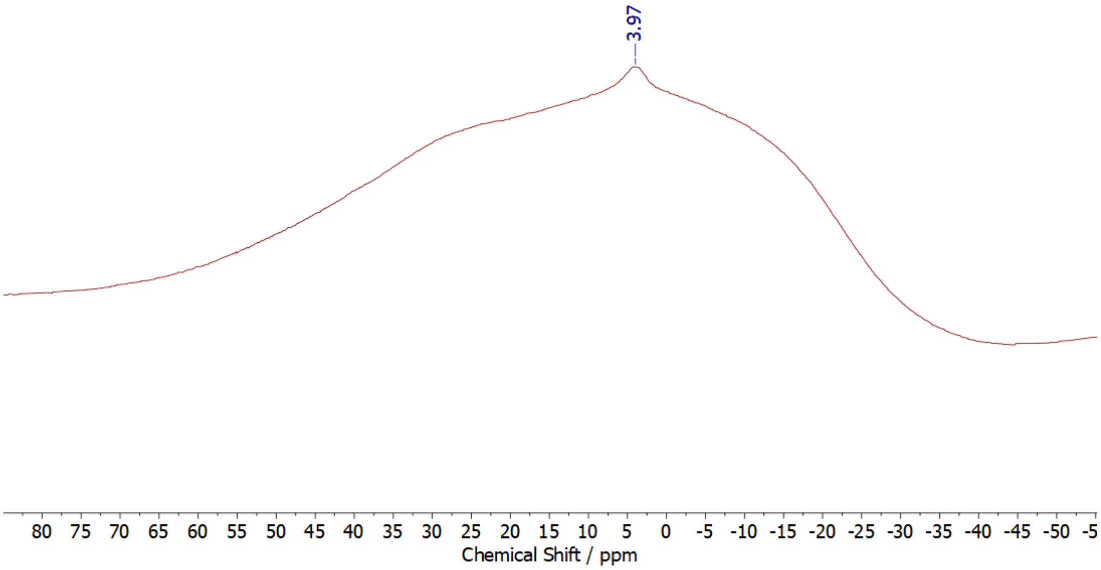

Figure S21. $^{11}B\{^{1}H\}$ NMR spectrum of the reaction of **2** with 0.5 eq. $B(C_6F_5)_3$ to generate **4**, C_6D_6, 25°C.

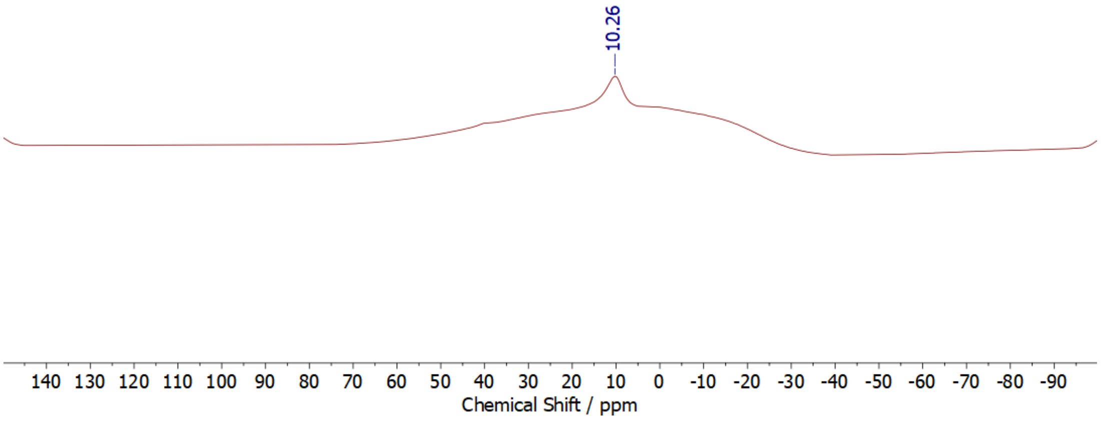

Figure S22. $^{11}P\{^{1}H\}$ NMR spectrum of the reaction of **2** with 1.0 eq. $B(C_6F_5)_3$ to generate **4**, C_6D_6, 25°C.

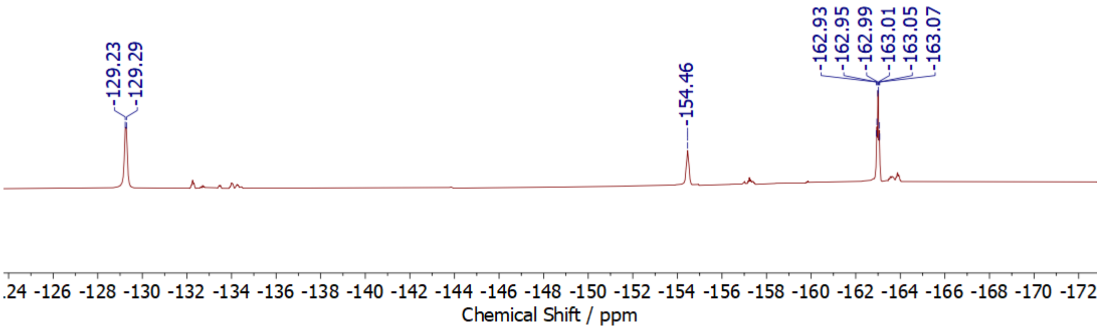

Figure S23. $^{19}F\{^{1}H\}$ NMR spectrum of the reaction of **2** with 0.5 eq. $B(C_6F_5)_3$ to generate **4**, C_6D_6, 25°C.

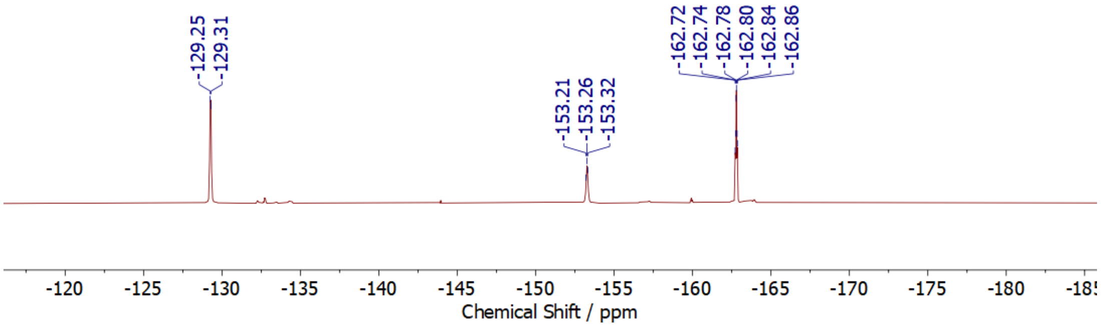

Figure S24. $^{19}F\{^{1}H\}$ NMR spectrum of the reaction of **2** with 1.0 eq. $B(C_6F_5)_3$ to generate **4**, C_6D_6, 25°C.

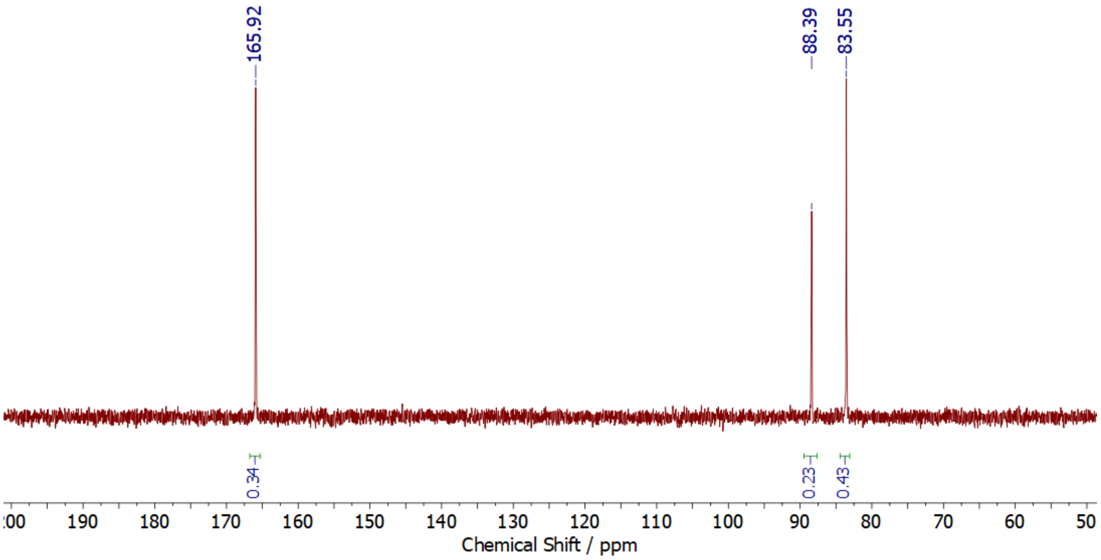

Figure S25. $^{31}P\{^{1}H\}$ NMR spectrum of the reaction of **2** with 10 equivalents of *para*-anisidine producing **5a/b**, $CDCl_3$, 25°C, partial precipitation of *para*-anisidine prevents the extraction of an equilibrium constant.

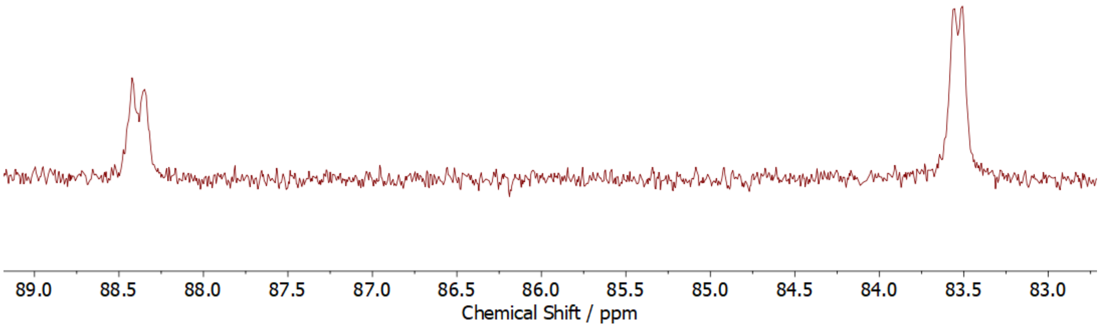

Figure S26. ^{31}P NMR spectrum of the reaction of **2** with 10 equivalents of *para*-anisidine producing **5a/b**, $CDCl_3$, 25°C.

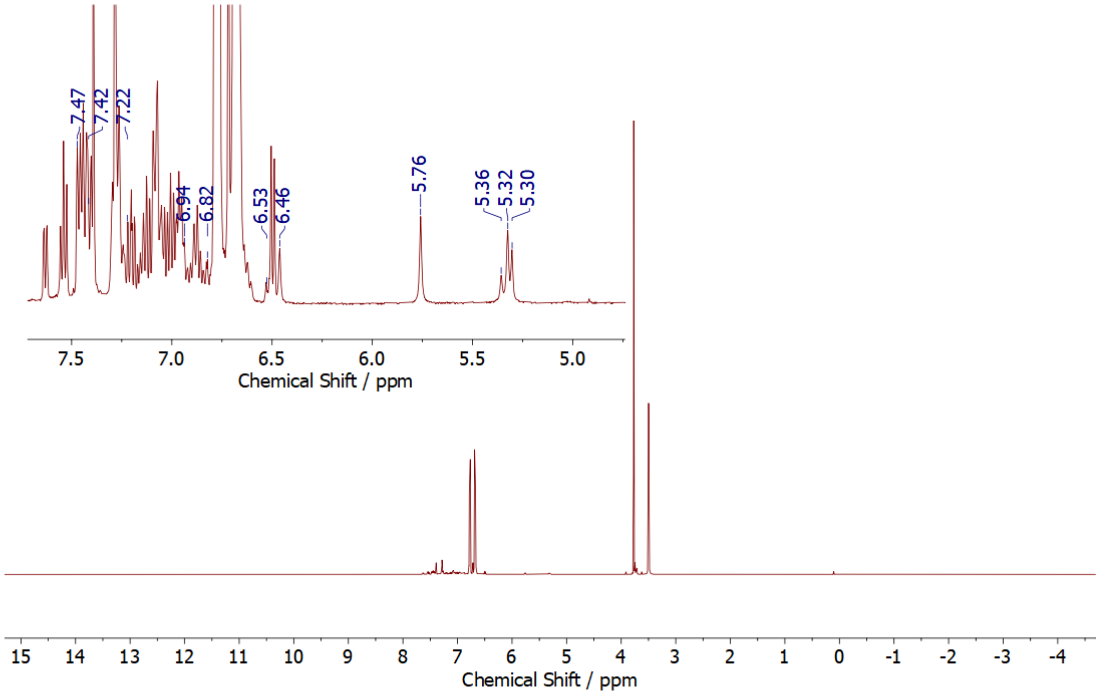

Figure S27. ^{1}H NMR spectrum of the reaction of **2** with 10 equivalents of *para*-anisidine producing **5a/b**, $CDCl_3$, 25°C.

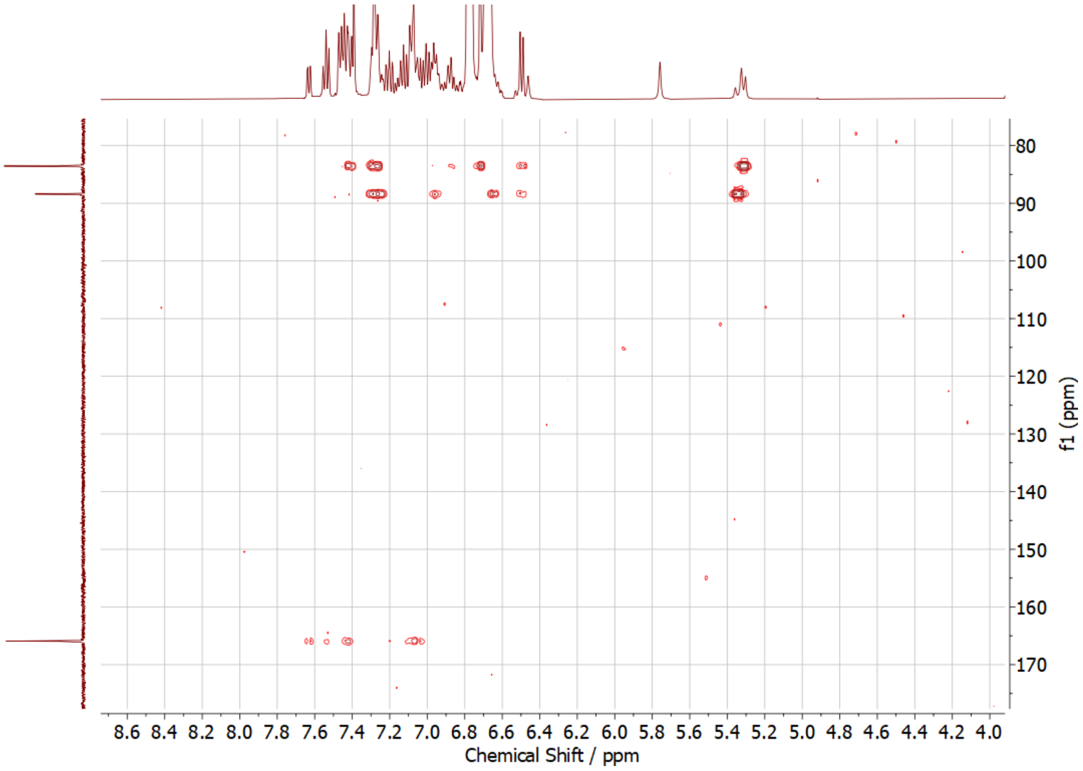

Figure S28. ^{31}P–^{1}H HMQC NMR spectrum of the reaction of **2** with 10 equivalents of *para*-anisidine producing **5a/b**, $CDCl_3$, 25°C.

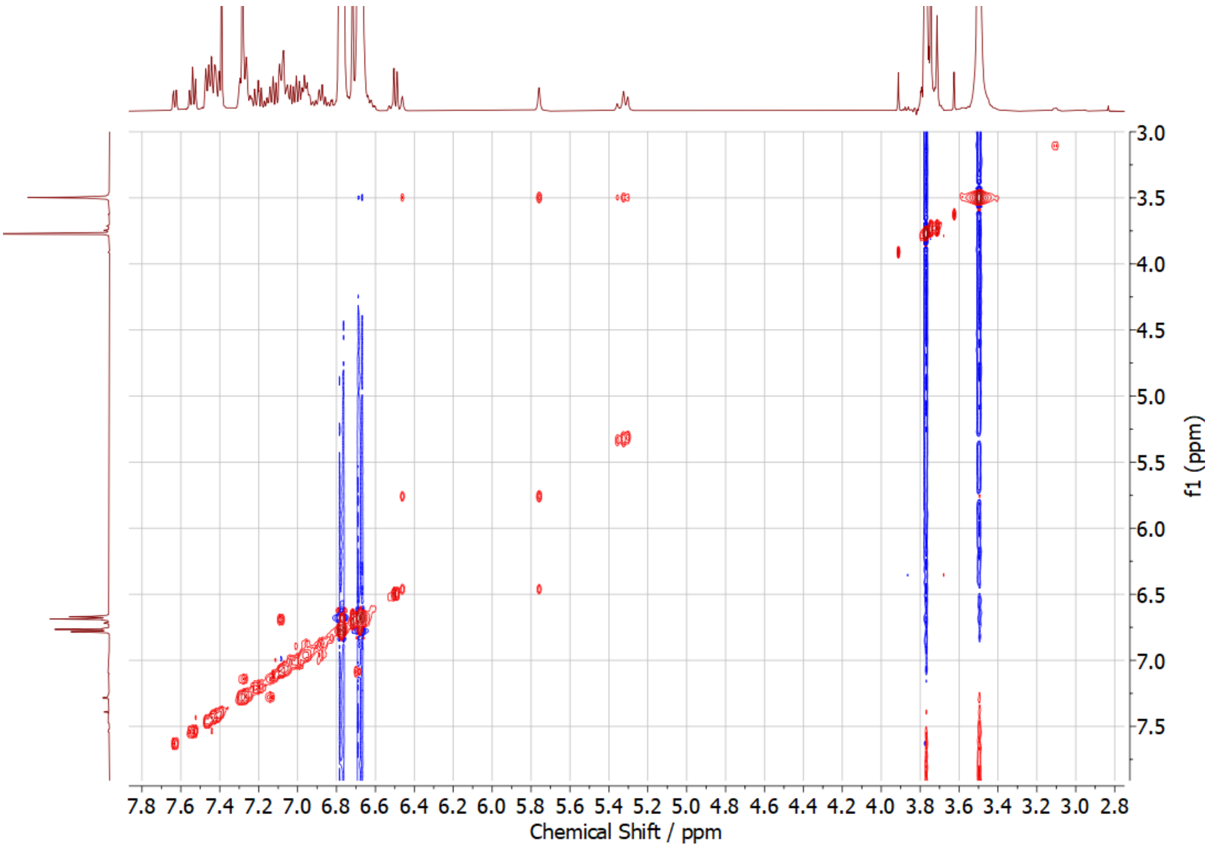

Figure S29. ^{1}H-EXSY NMR spectrum of the reaction of **2** with 10 equivalents of *para*-anisidine producing **5a/b**, $CDCl_3$, 25°C.

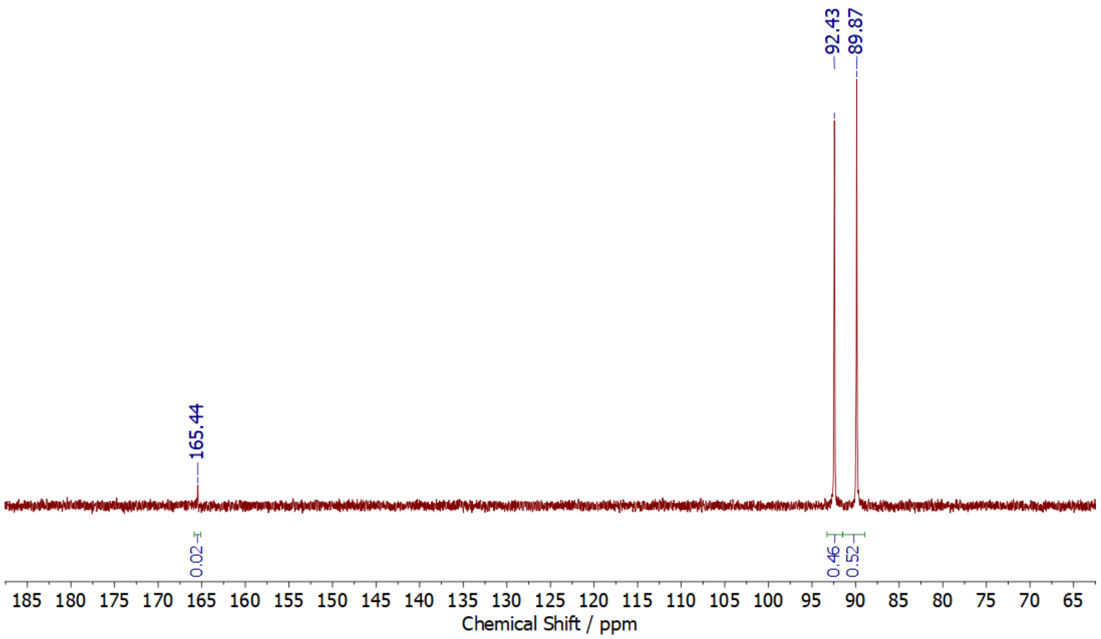

Figure S30. $^{31}P\{^{1}H\}$ NMR spectrum of **2** under one ammonia atmosphere to generate **6a/b**, THF-d_8, 25°C.

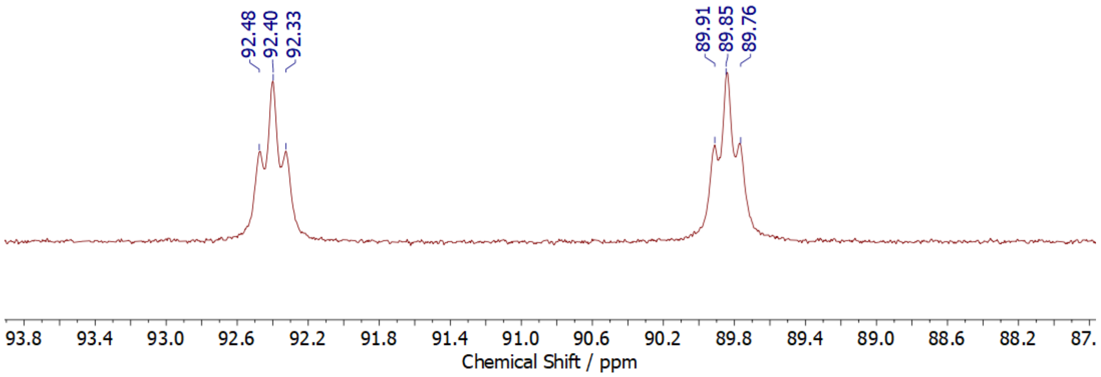

Figure S31. ^{31}P NMR spectrum of **2** under one ammonia atmosphere to generate **6a/b**, THF-d_8, 25°C.

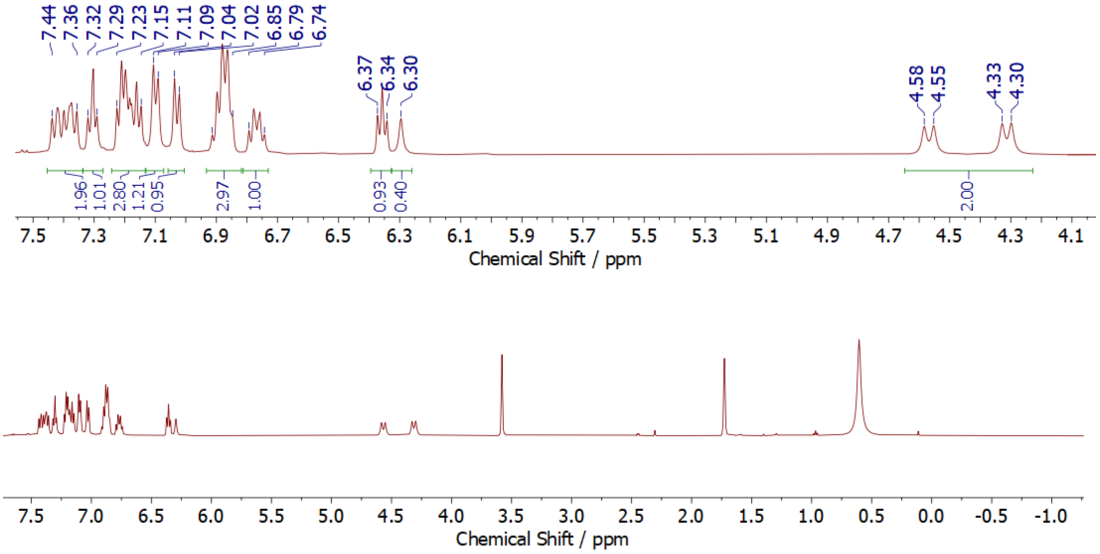

Figure S 32. ^{1}H NMR spectrum of **2** under one ammonia atmosphere to generate **6a/b**, THF-d_8, 25°C.

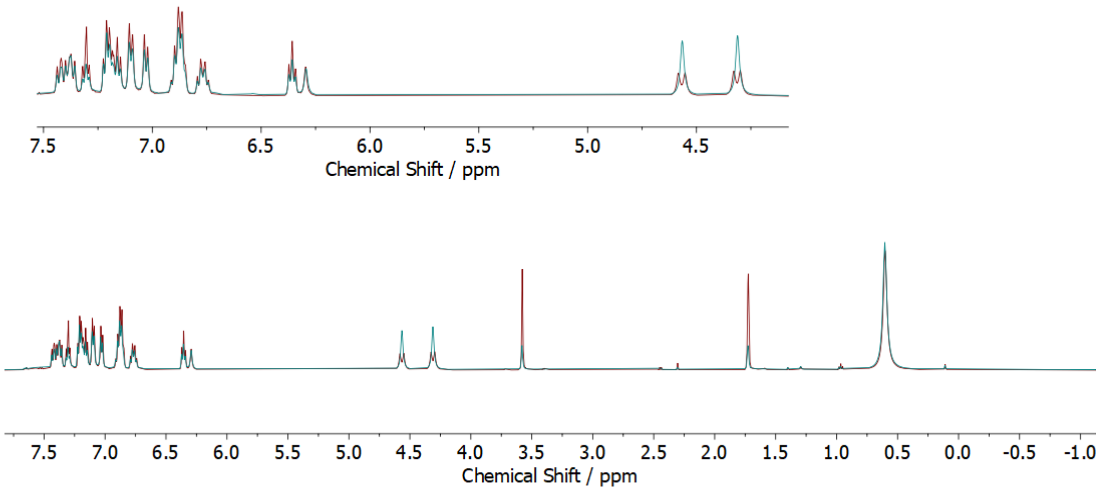

Figure S33. ^{1}H (red) and $^{1}H\{^{31}P\}$ (teal) NMR spectrum of **2** under one ammonia atmosphere to generate **6a/b**, THF-d_8, 25°C.

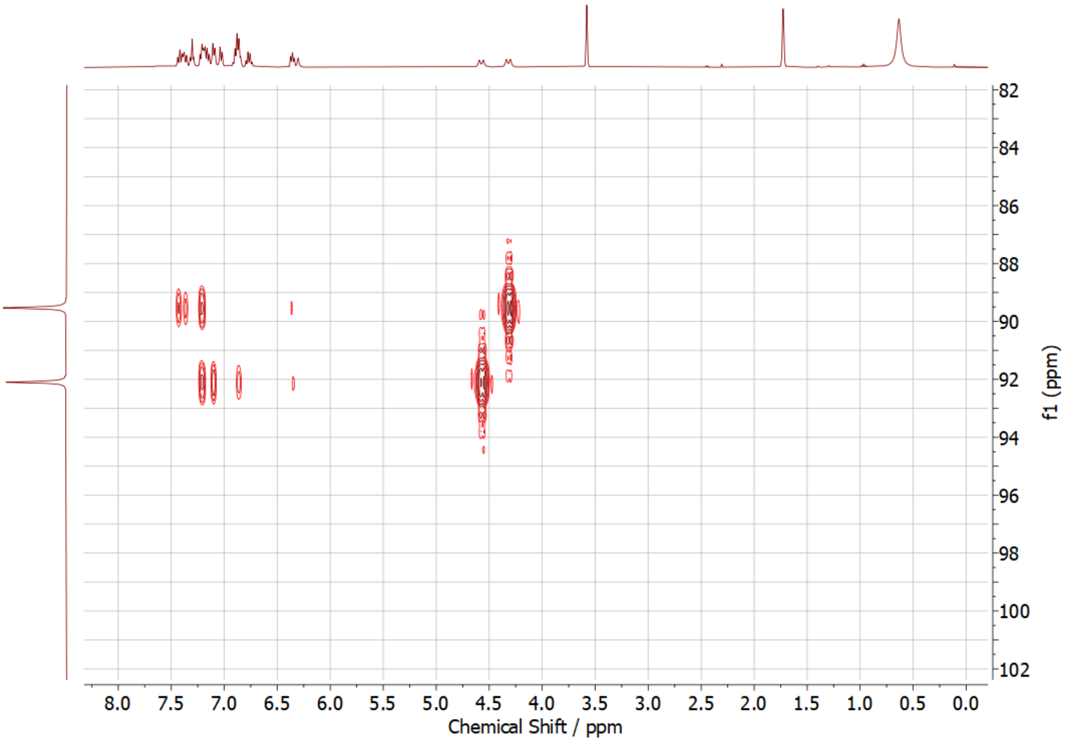

Figure S34. ^{31}P–^{1}H HMQC NMR spectrum of the reaction of **2** under one ammonia atmosphere to generate **6a/b**, THF-d_8, 25°C.

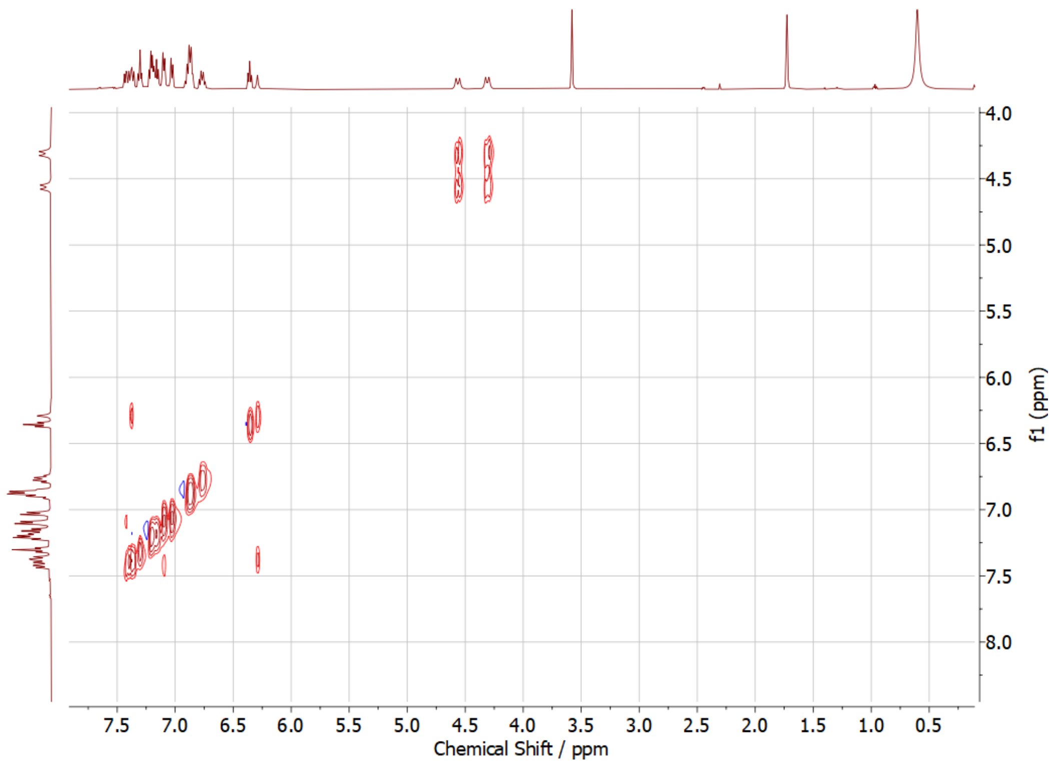

Figure S35. ^{1}H EXSY NMR spectrum of **2** under one ammonia atmosphere to generate **6a/b**, THF-d_8, 25°C.

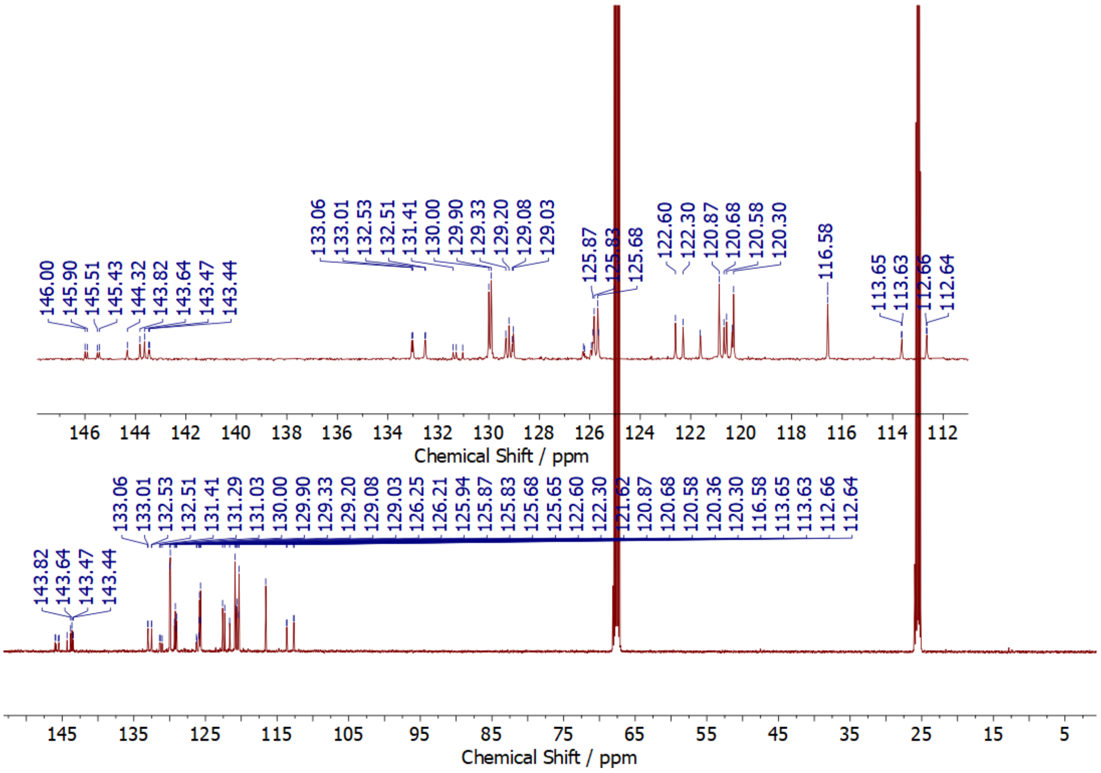

Figure S36. $^{13}C\{^{1}H\}$ NMR spectrum of **2** under one ammonia atmosphere to generate **6a/b**, THF-d_8, 25°C.

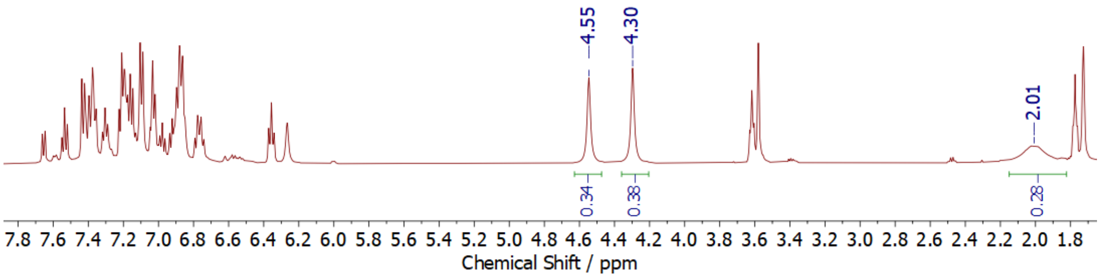

Figure S37. ^{1}H NMR spectrum of **6a/b** after removal of the solvent and redissolving, THF-d_8, 25°C. The integrals of the aminophosphines (4.55 ppm and 4.30 ppm) in relation to residual ammonia (2.01 ppm) showcases that ammonia is partially removed upon drying.

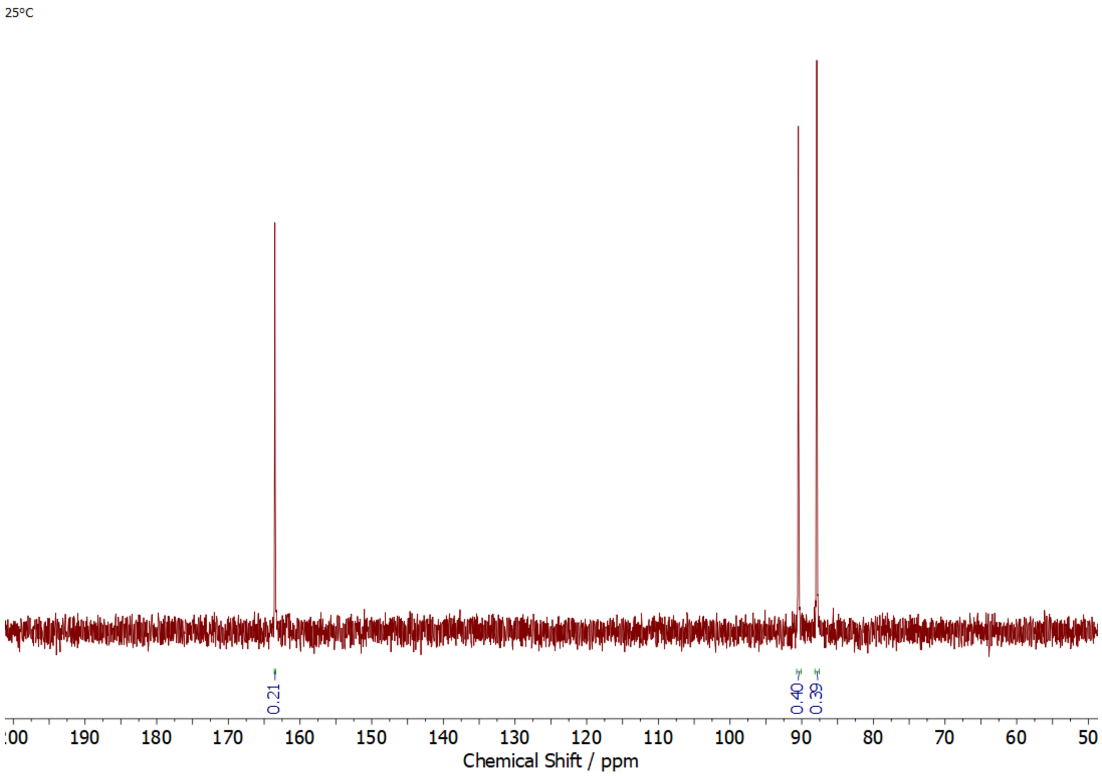

Figure S38. $^{31}P\{^{1}H\}$ NMR spectrum of **6a/b** after removal of the solvent and redissolving, THF-d_8, 25°C.

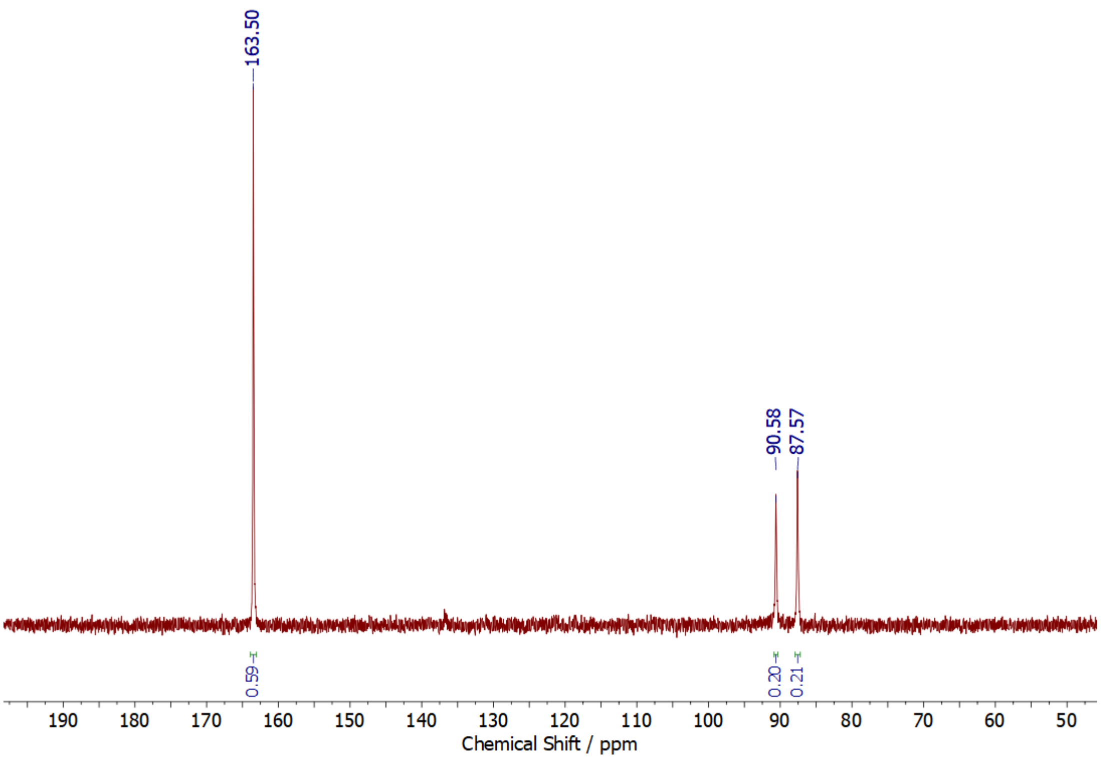

Figure S39. $^{31}P\{^{1}H\}$ NMR spectrum of **6a/b** after removal of the solvent and redissolving, THF-d_8, 60°C, 1h equilibration.

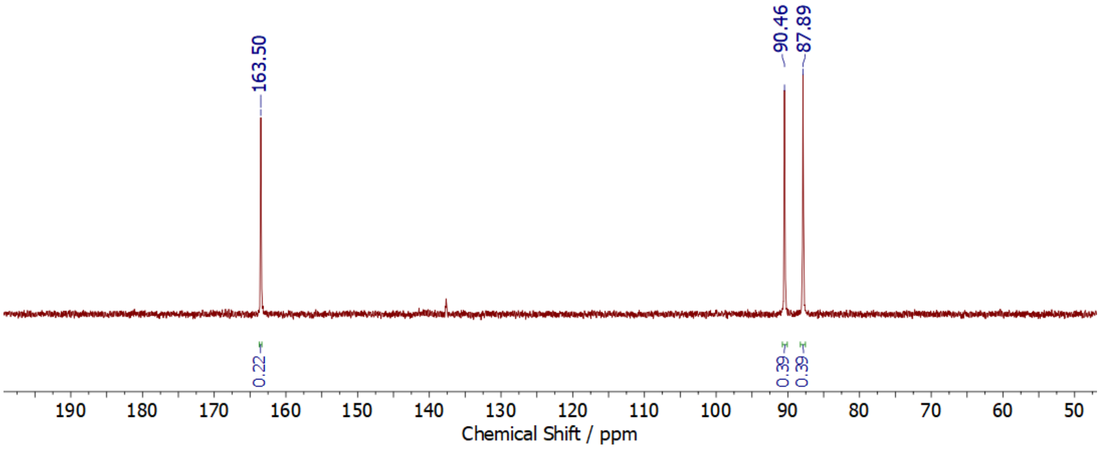

Figure S40. $^{31}P\{^{1}H\}$ NMR spectrum of **6a/b** after removal of the solvent and redissolving, THF-d_8, 25°C, after VT-NMR.

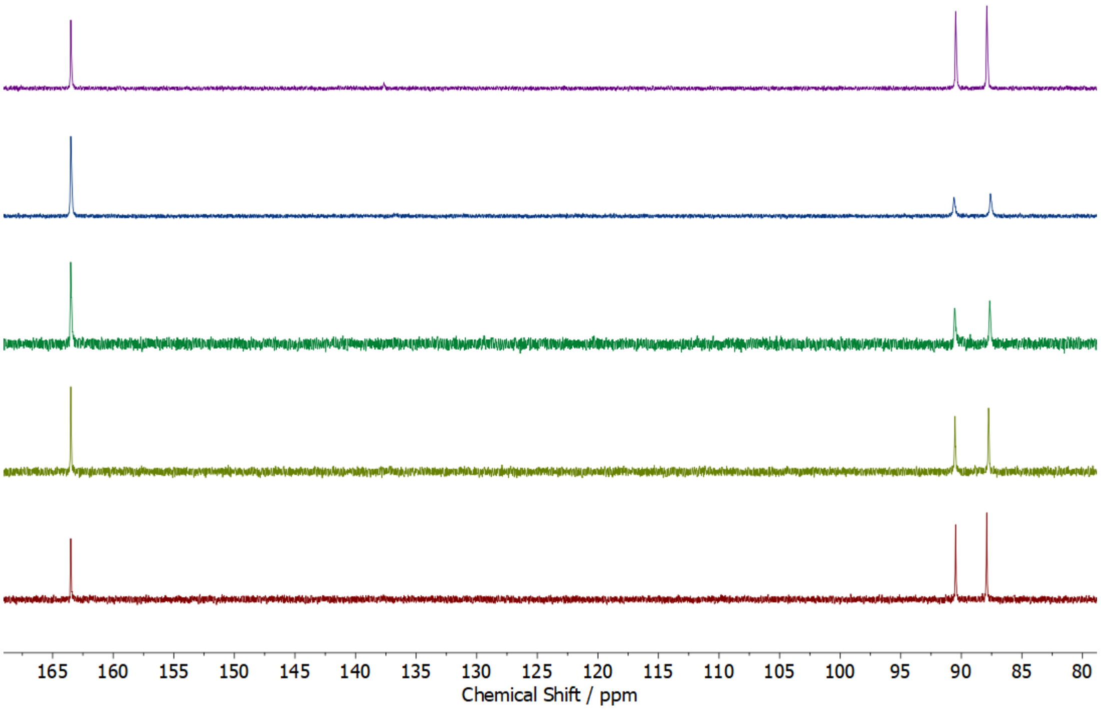

Figure S41. $^{31}P\{^1H\}$ NMR spectra of **6a/b** after removal of the solvent and redissolving, THF-d_8, 25°C (red, 21% **2**), 40°C (yellow, 33% **2**), 50°C (green, 43% **2**), 60°C (blue, 59% **2**), after VT at 25°C (purple 22% **2**), 1h equilibration each, except for the last measurement (8 h).

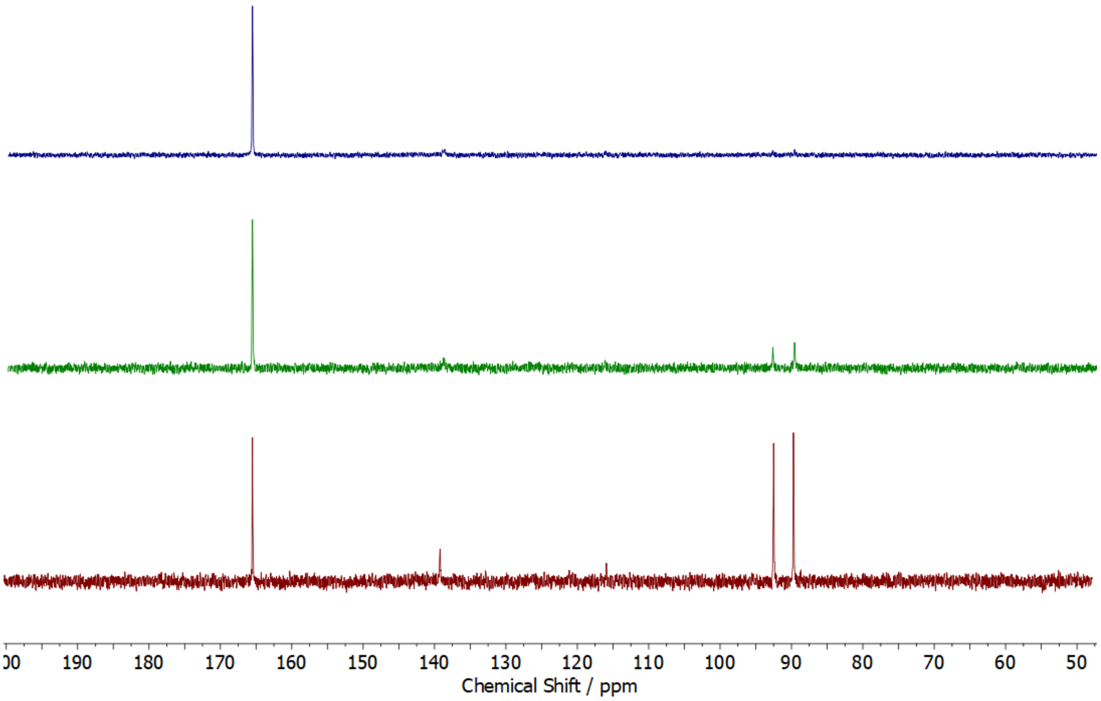

Figure S42. $^{31}P\{^1H\}$ NMR spectra of **6a/b** after removal of the solvent, redissolving in THF and degassing, 40°C for 1h (red), 60°C for 10 minutes (green), 60°C for 20 minutes (blue).

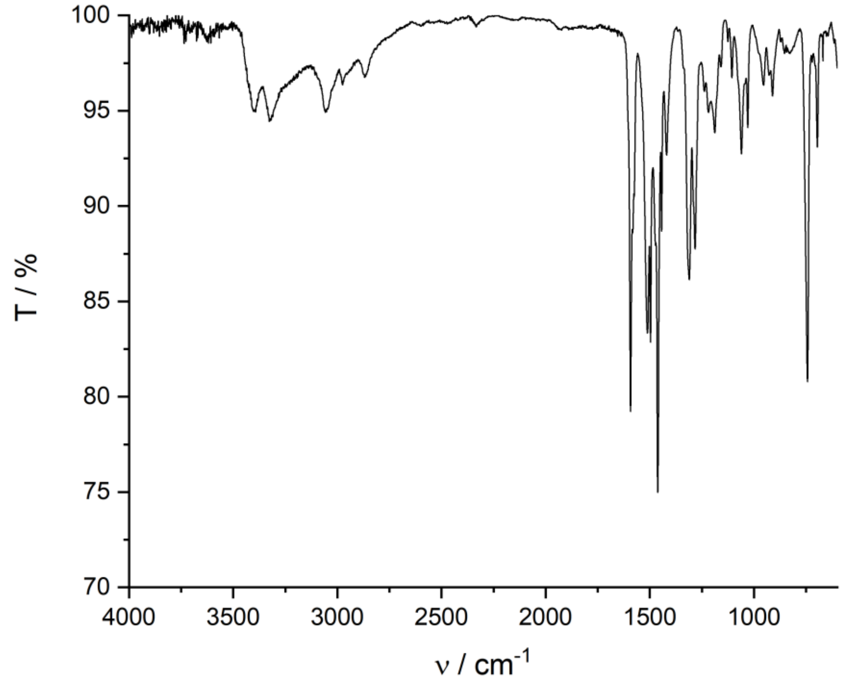

Figure S43. IR spectrum of **6a/b**, neat, ATR, 25°C.

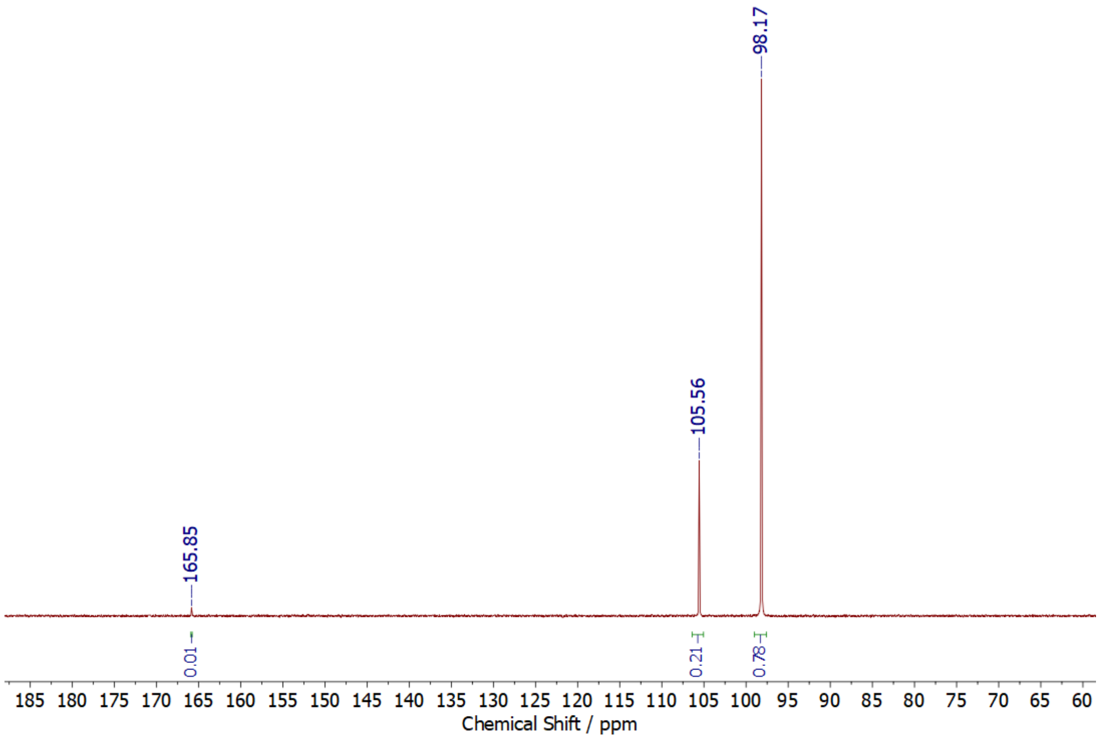

Figure S44. $^{31}P\{^{1}H\}$ NMR spectrum of **7a/b** prepared *via* route A, C_6D_6, 25°C.

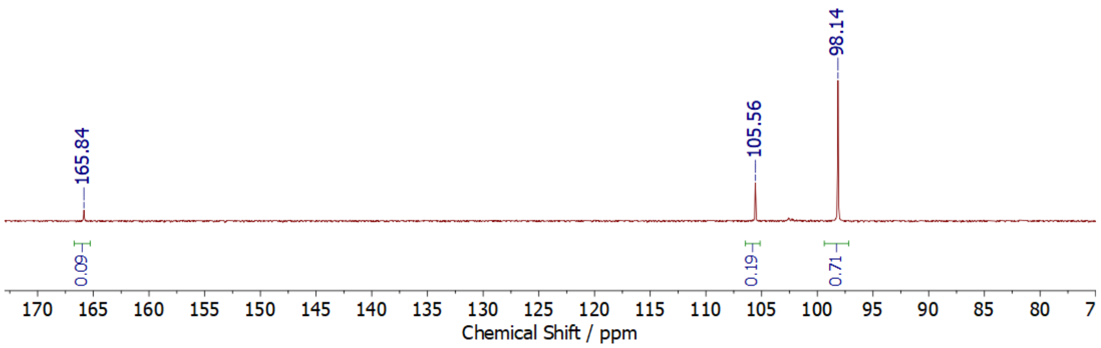

Figure S45. $^{31}P\{^{1}H\}$ NMR spectrum of **7a/b** prepared *via* route B, C_6D_6, 25°C.

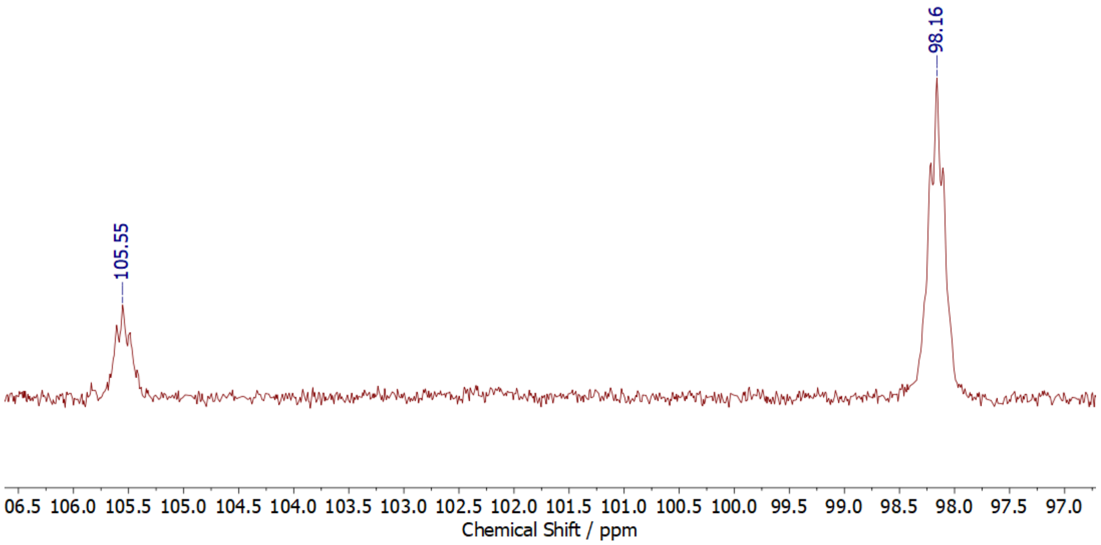

Figure S46. ^{31}P NMR spectrum of **7a/b**, C_6D_6, 25°C.

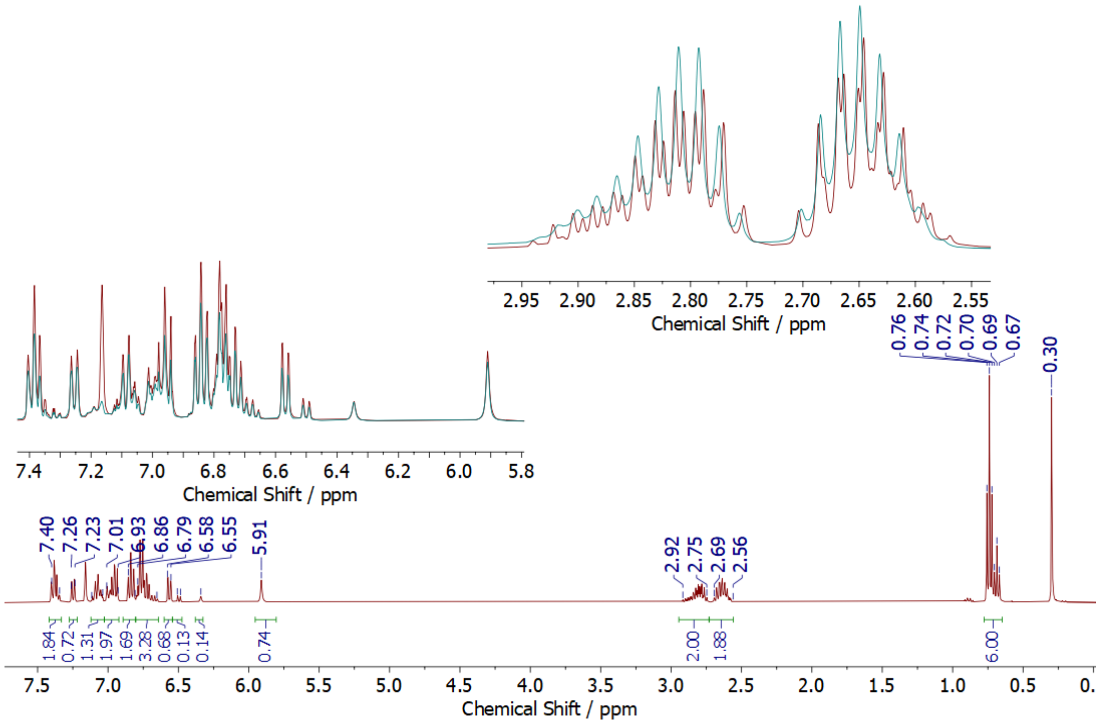

Figure S47. ^{1}H NMR spectrum of **7a/b** prepared *via* route A, C_6D_6, 25°C, a grease impurity is detected at 0.30 ppm; inset: $^{1}H\{^{31}P\}$ NMR spectrum of **7a/b**, C_6D_6, 25°C.

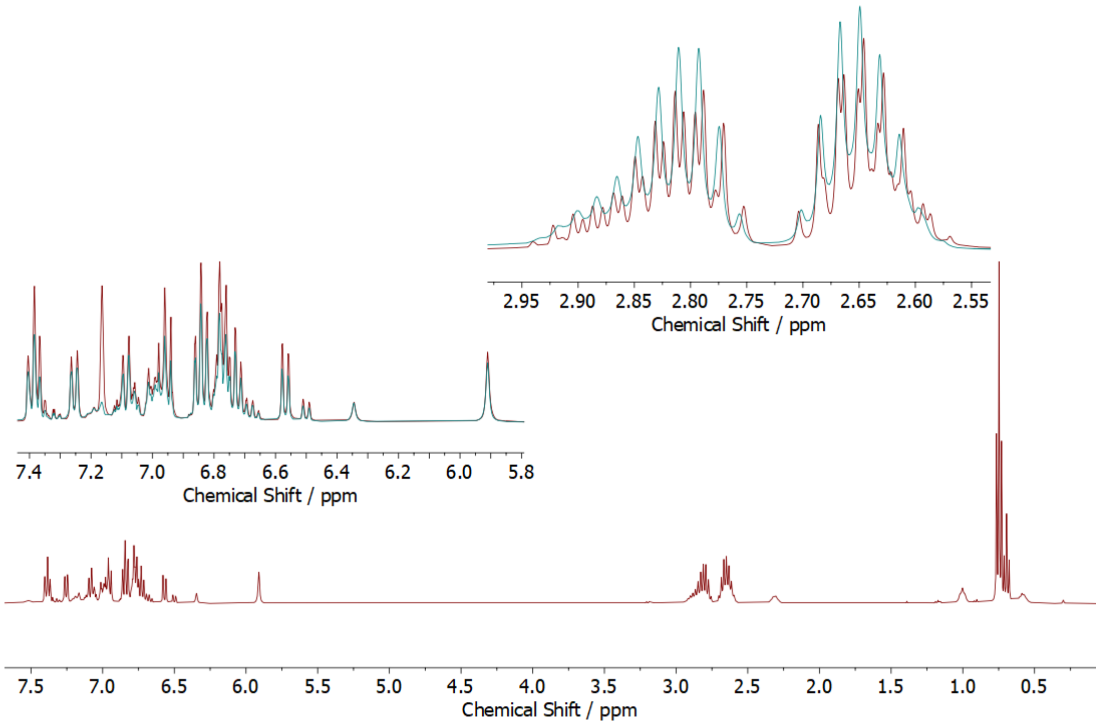

Figure S48. ^{1}H NMR spectrum of **7a/b** prepared *via* route B, C_6D_6, 25°C, residual trimethylamine hydrochloride is detected at 2.32 and 1.01 ppm; inset: $^{1}H\{^{31}P\}$ NMR spectrum of **7a/b**, C_6D_6, 25°C.

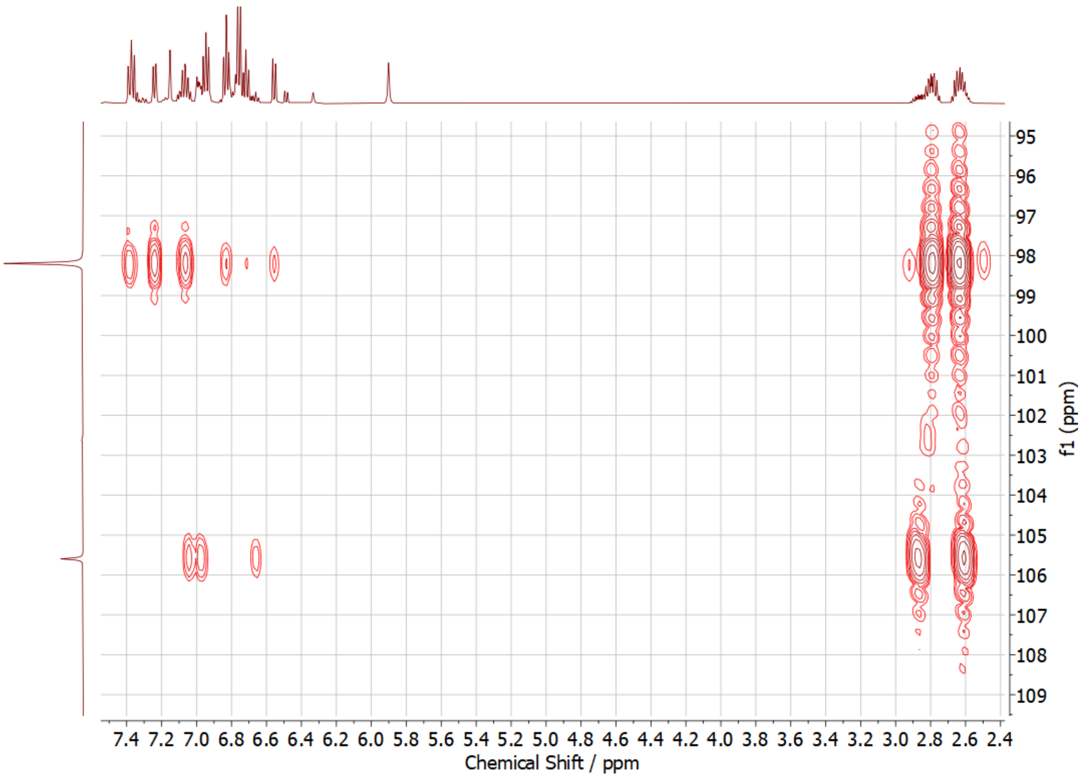

Figure S49. ^{31}P–^{1}H HMQC NMR spectrum of 7**a/b**, C_6D_6, 25°C.

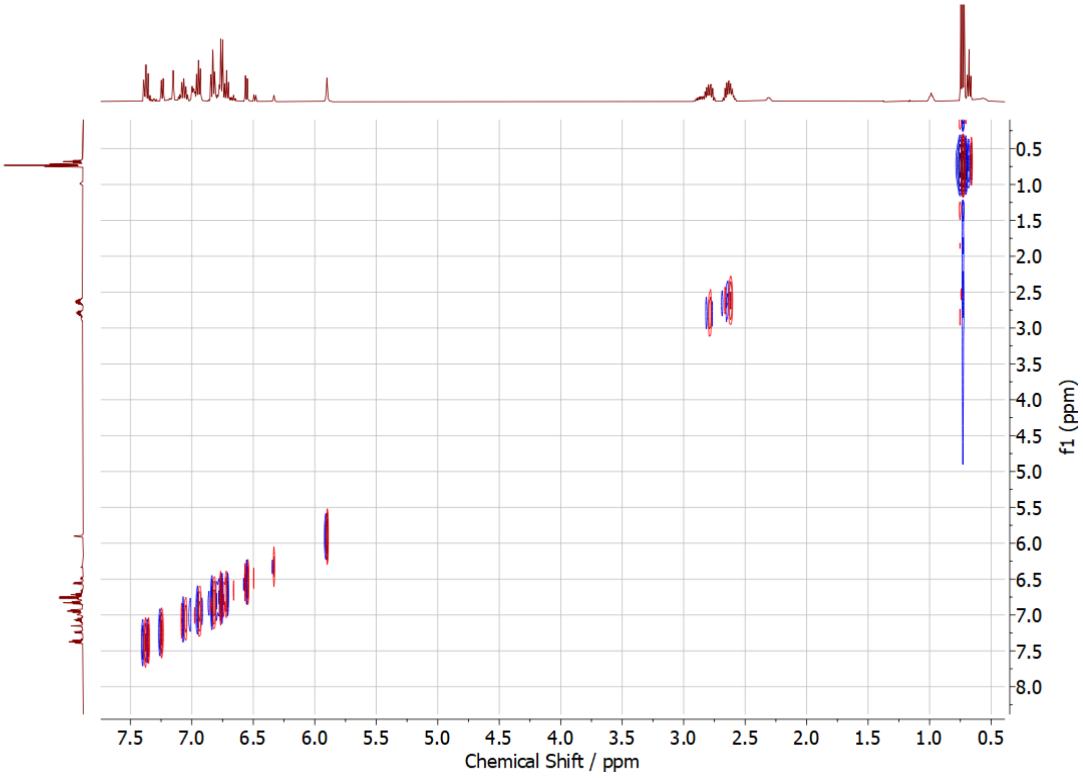

Figure S50. ^{1}H EXSY NMR spectrum of **7a/b**, C_6D_6, 25°C.

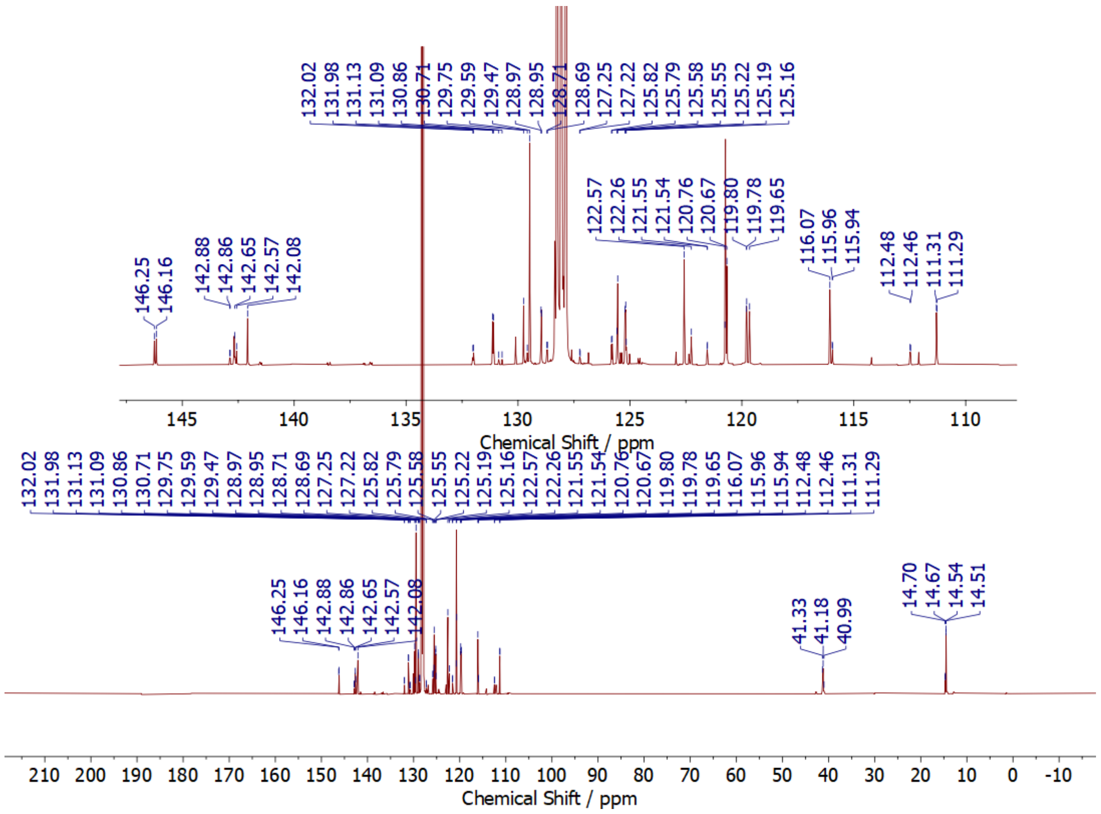

Figure S51. $^{13}C\{^{1}H\}$ NMR spectrum of 7**a/b**, C_6D_6, 25°C, unlabelled peaks correspond to small amounts of **2** which are being regenerated upon extensive drying.

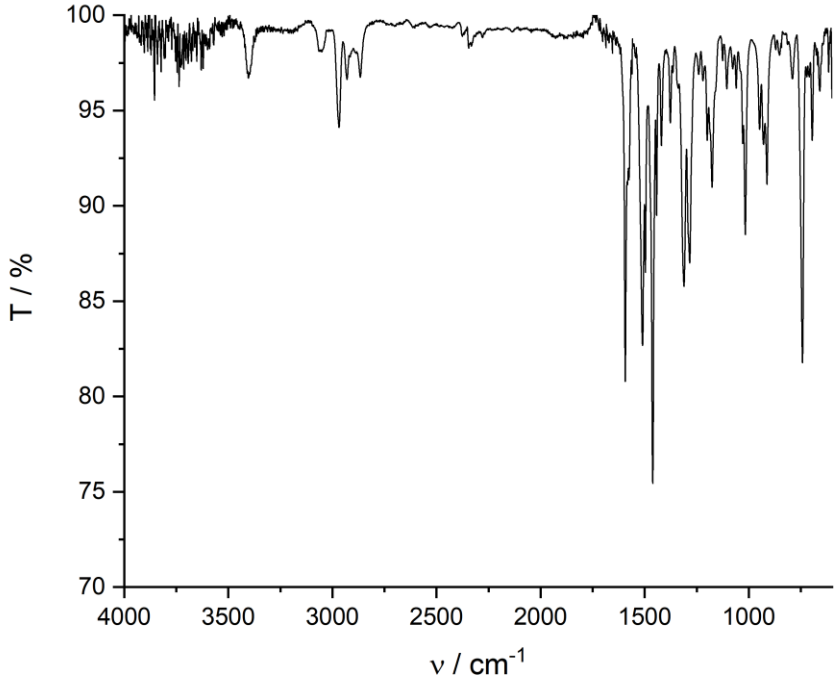

Figure S52. IR spectrum of **7a/b**, neat, ATR, 25°C.

Single crystal X-ray diffraction data

Table S1. Selected X-ray data collection and refinement parameters for **2**, **3**, **4** and **7b**.

	**2**	**3**	**4**	**7b**
Formula	$C_{18}H_{13}N_2PS$	$C_{18}H_{13}N_2PSSe$	$C_{36}H_{13}BF_{15}N_2PS$	$C_{22}H_{24}N_3PS$
CCDC	2097495	2097496	2097497	2097498
Fw [g mol^{-1}]	320.33	399.29	832.32	393.47
Crystal system	monoclinic	monoclinic	monoclinic	monoclinic
Space group	$P2_1/n$	$P2_1/n$	$P2_1/n$	$P2_1/n$
a (Å)	9.6578(2)	13.9642(7)	11.6192(2)	11.0697(2)
b (Å)	12.5738(2)	8.8709(3)	13.9694(2)	13.5582(3)
c (Å)	24.4697(6)	14.6930(7)	19.1350(3)	13.8325(3)
α (°)	90	90	90	90
β (°)	91.155(2)	115.169(6)	97.1090(10)	104.197(2)
γ (°)	90	90	90	90
V (Å^3)	2970.88(11)	1647.29(15)	3081.99(8)	2012.65(7)
Z	8	4	4	4
Radiation, λ (Å)	Cu Kα, 1.54184	Cu Kα, 1.54184	Cu Kα, 1.54184	Cu Kα, 1.54184
Temp (K)	150(2)	150(2)	150(2)	150(2)
ρ_{calc} (g cm^{-3})	1.432	1.610	1.794	1.299
μ (mm^{-1})	2.914	5.178	2.590	2.258
Reflections collected	39432	8529	37083	10305
Independent reflections	6178	3409	6436	4156
Parameters	397	208	505	250
R(int)	0.0550	0.0323	0.0448	0.0296
R1/wR2,[a] I ≥ 2σI (%)	3.50/8.36	4.16/10.84	4.00/10.40	3.43/8.26
R1/wR2,[a] all data (%)	5.28/9.07	4.64/11.45	4.86/11.09	4.53/8.97
GOF	1.032	1.036	1.036	1.043

$R1 = [\Sigma||F_o| - |F_c||]/\Sigma|F_o|$; $wR2 = \{[\Sigma w[(F_o)^2 - (F_c)^2]^2]/[\Sigma w(F_o{}^2)^2\}^{1/2}$; $w = [\sigma^2(F_o)^2 + (AP)^2 + BP]^{-1}$, where $P = [(F_o)^2 + 2(F_c)^2]/3$ and the A and B values are 0.0456 and 0.30 for **2**. 0.0718 and 1.11 for **3**, 0.0662 and 1.26 for **4**, and 0.0398 and 0.59 for **7b**.

Computational Details

Geometry optimizations, frequency calculations and PCM solvent corrections were run with Gaussian 16 Revision C.01[4][6] using the BP86[5,6] functional. For geometry optimisations, P and S centres were described with Stuttgart RECPs and associated basis sets,[7] with added d-orbital polarization on P (ζ = 0.387) and Si (ζ = 0.503).[8] 6-31G** basis sets were used for C, H, B, N, O and F.[9,10] Single point energy calculations were performed on the optimised geometries, at the BP86/def2-TZVP level of theory.[11] Stationary points were fully characterized using analytical frequency calculations as either minima (all positive eigenvalues) or transition states (one negative eigenvalue). IRC calculations and subsequent geometry optimizations were used to confirm the minima linked by the transition states. Energies reported in the text are based on the gas-phase free energies and incorporate a correction for dispersion effects using Grimme's D3 parameter set[12] (i.e. BP86-D3) as well as solvation (PCM approach) in benzene, tetrahydrofuran (THF) or chloroform. Energies are given in atomic units unless otherwise stated.

In the case of **2TS**, many efforts were made to fully optimise to a transition state, all of which resulted in relaxation towards **2**. However, a single point energy calculation of a partially optimised geometry found in a potential energy surface scan resulted in a stationary point which has one negative eigenvalue. Visualisation of this vibration shows the desired S–P–N bending mode.

Intrinsic bond orbital (IBO) calculations[13,14] were performed and visualised on the optimised IRC geometries using *IboView* at the PBE0/def2-TZVP level of theory. Density fitting was employed using univ-JFIT basis sets.[15]

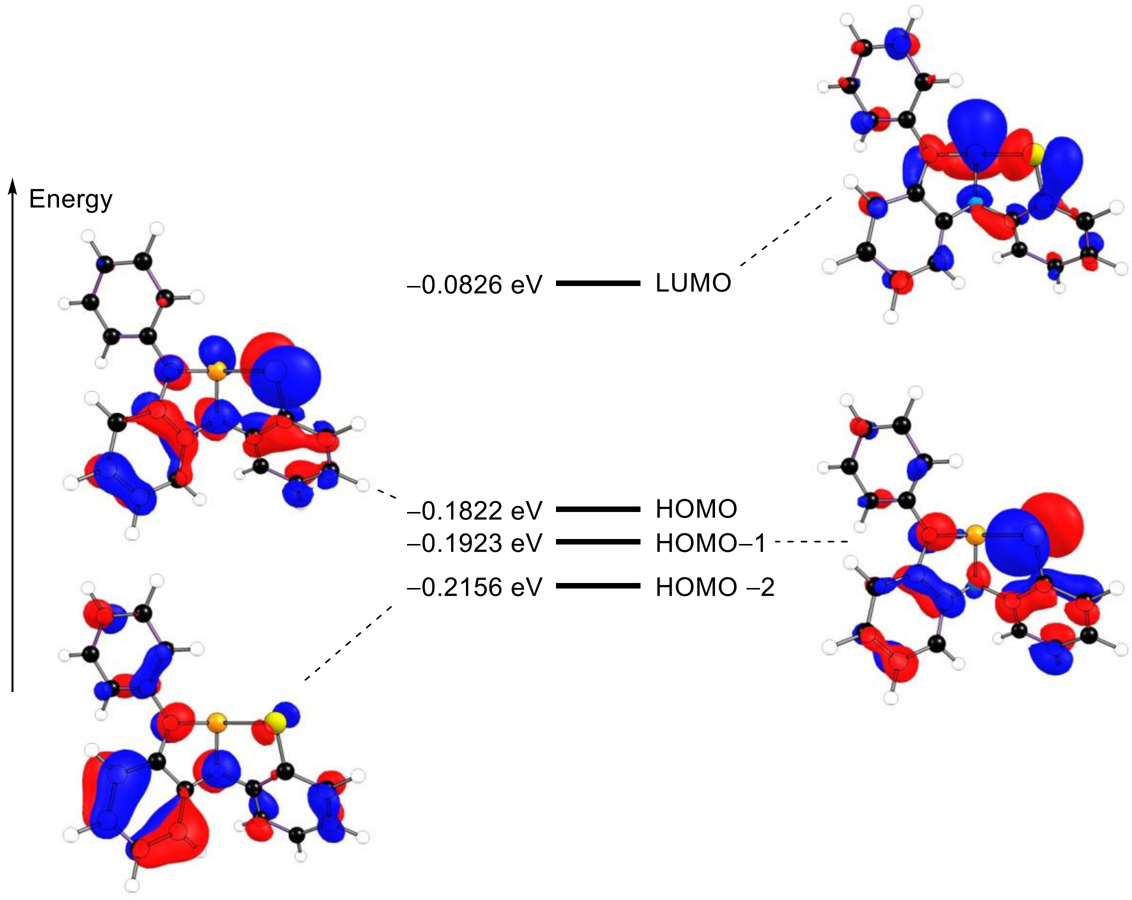

Figure S53. Molecular orbital (BP86/def2TZVP//SDDALL/6-31g**) diagram of **2**.

Table S1. Calculated energies (kcal/mol; BP86-D3/def2TZVP/PCM) relative to free **2** + free substrate.

**Structure**	**Relative Energy (kcal/mol)**
**2TS**	10.8
**2+BCF**	–2.3
**4TS**	–1.8
**4a**	–3.6
**4b**	1.5
**5a**	–0.7
**5b**	–0.9
**5c**	3.7
**6TScoop**	22.0
**6TSox**	56.0
**6a**	0.0
**6b**	-0.4
**6c**	4.9
**7a**	–4.0
**7b**	–3.3
**7c**	5.8
**SH**	6.7

The computed HOMO of **2** suggests that coordination of $B(C_6F_5)_3$ towards the sulfur atom would also be feasible. The P–B adduct **4a** was computed to be thermodynamically favored (ΔG_{DFT} = –3.6 kcal/mol) over S–B coordination (**4b**, ΔG_{DFT} = +1.5 kcal/mol). Formation of **4a** is associated with a minor activation barrier (**4TS**, $\Delta G^{‡}_{DFT}$ = +-1.8 kcal/mol) after encounter complex formation (**2+BCF**, ΔG_{DFT} = –2.3 kcal/mol).

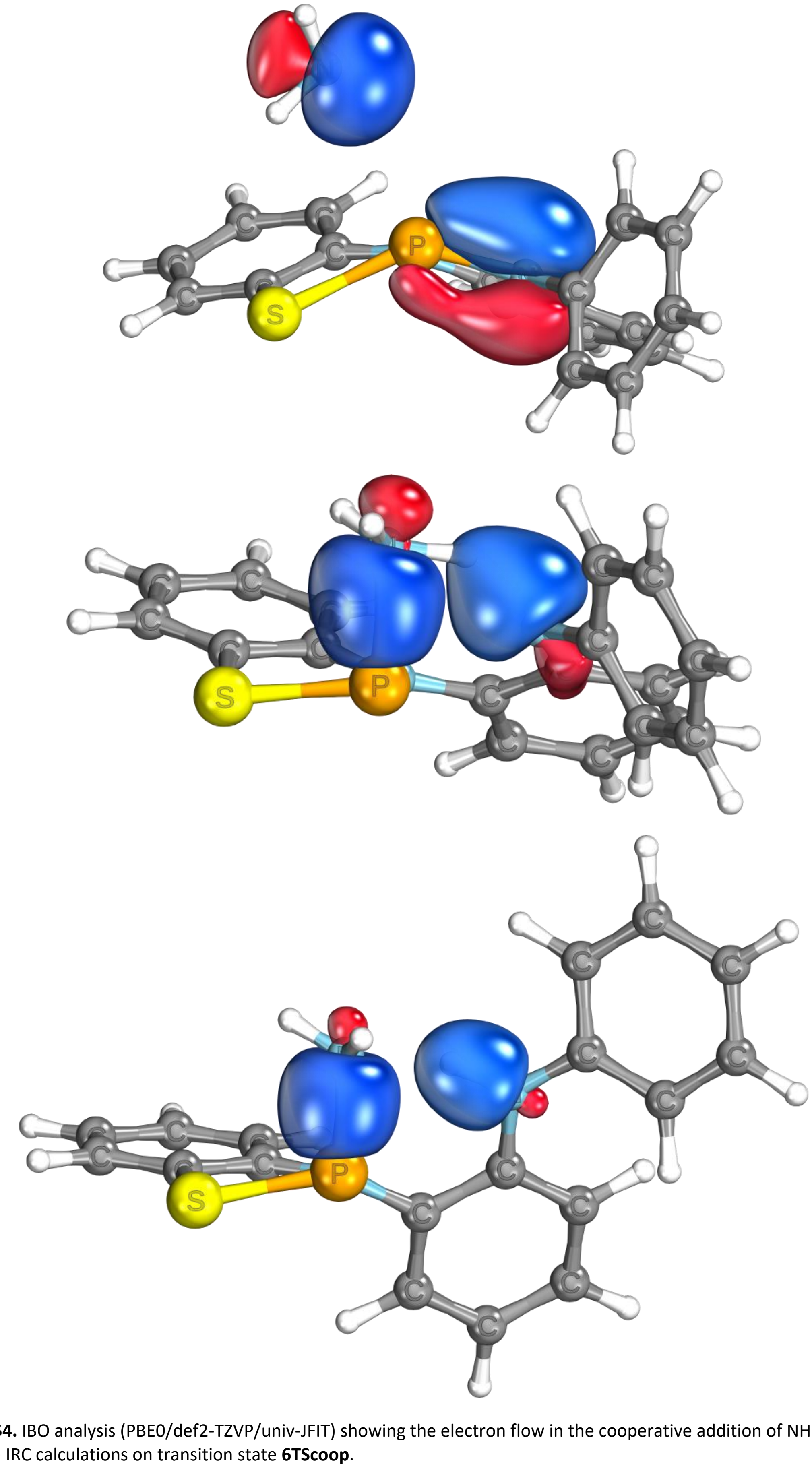

Figure S54. IBO analysis (PBE0/def2-TZVP/univ-JFIT) showing the electron flow in the cooperative addition of NH_3 to **6a** using the IRC calculations on transition state **6TScoop**.

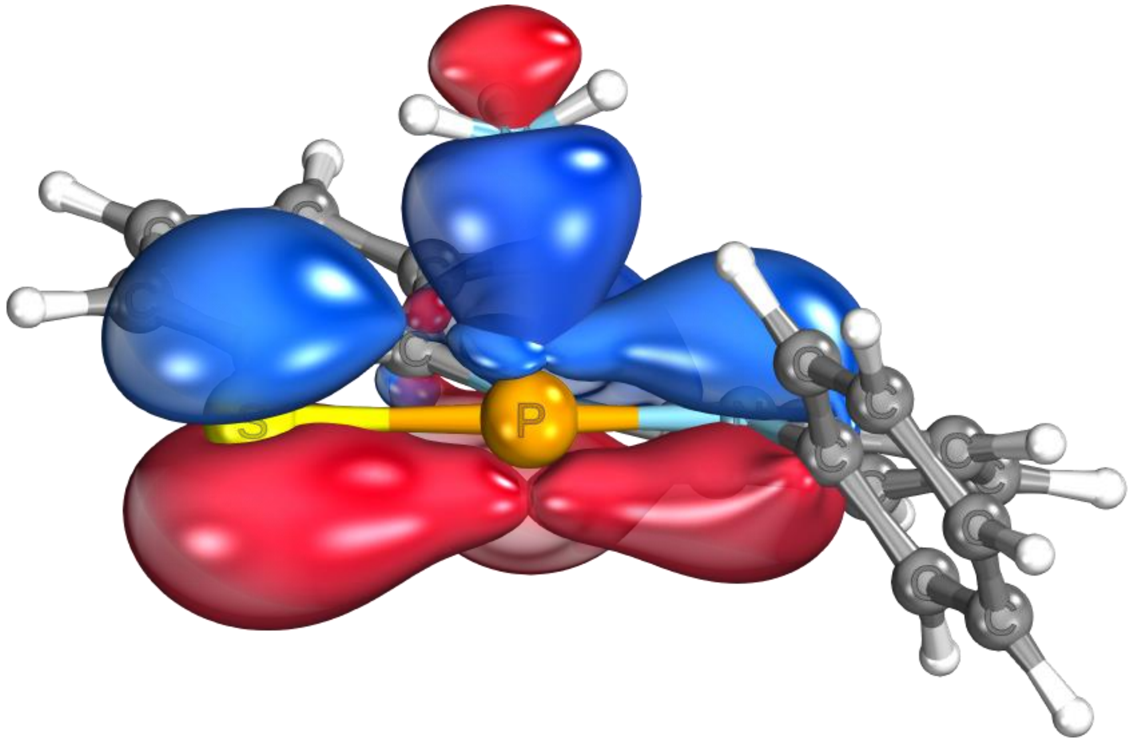

Figure S55. IBO analysis (PBE0/def2-TZVP/univ-JFIT) showing the NH_3–P donation in the formation of a T-shaped phosphine and its stabilization by the adjacent donor atoms of the pincer ligands.

DFT optimised geometries

NH3

SCF = -56.5546454199

H(0 K) = -56.521091

H(298 K) = -56.517281

G(298 K) = -56.540192

SCF+D3 = -56.5546875199

PCM SCF (THF) = -56.5592736461

BS2 (def2-tzvp) = -56.5838760737

Low Freq. = 1071.9824 cm^{-1}, 1650.1618 cm^{-1}

pOMeNH2

SCF = -402.124761046

H(0 K) = -401.979344

H(298 K) = -401.969806

G(298 K) = -402.012264

SCF+D3 = -402.137942816

PCM SCF (Chloroform) = -402.130558702

BS2 (def2-tzvp) = -402.277509814

Low Freq. = 81.8914 cm^{-1}, 151.6499 cm^{-1}

Et2NH

SCF = -213.793966172

H(0 K) = -213.648581

H(298 K) = -213.640612

G(298 K) = -213.678797

SCF+D3 = -213.803786932

PCM SCF (THF) = -213.796105916

BS2 (def2-tzvp) = -213.868879814

Low Freq. = 107.9233 cm^{-1}, 113.9635 cm^{-1}

BCF

SCF = -2208.21458010

H(0 K) = -2208.065591

H(298 K) = -2208.034849

G(298 K) = -2208.128032

SCF+D3 = -2208.26002607

BS2 (def2-tzvp) = -2209.20564338

Low Freq. = 19.2390 cm^{-1}, 19.2559 cm^{-1}

2

SCF = -820.073758742

H(0 K) = -819.816166

H(298 K) = -819.797535

G(298 K) = -819.862608

SCF+D3 = -820.114885472

PCM SCF (THF) = -820.080358238

PCM SCF (Chloroform) = -820.079413008

BS2 (def2-tzvp) = -1543.21157760

Low Freq. = 32.2906 cm^{-1}, 41.9420 cm^{-1}

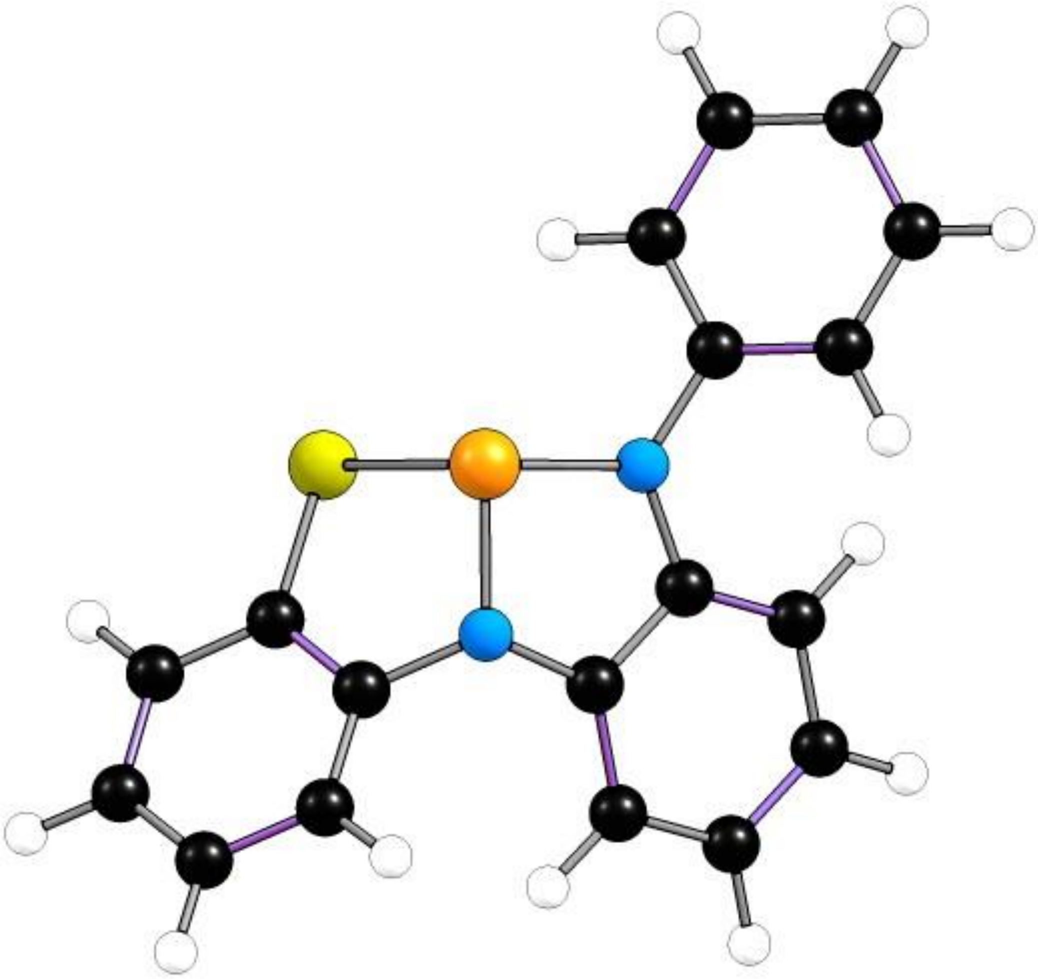

2TS

SCF = -820.061466323

H(0 K) = -819.804123

H(298 K) = -819.786191

G(298 K) = -819.849020

SCF+D3 = -820.100064343

PCM SCF (THF) = -820.068180499

BS2 (def2-tzvp) = -1543.19515181

Low Freq. = -67.4181 cm^{-1}, 40.9804 cm^{-1}

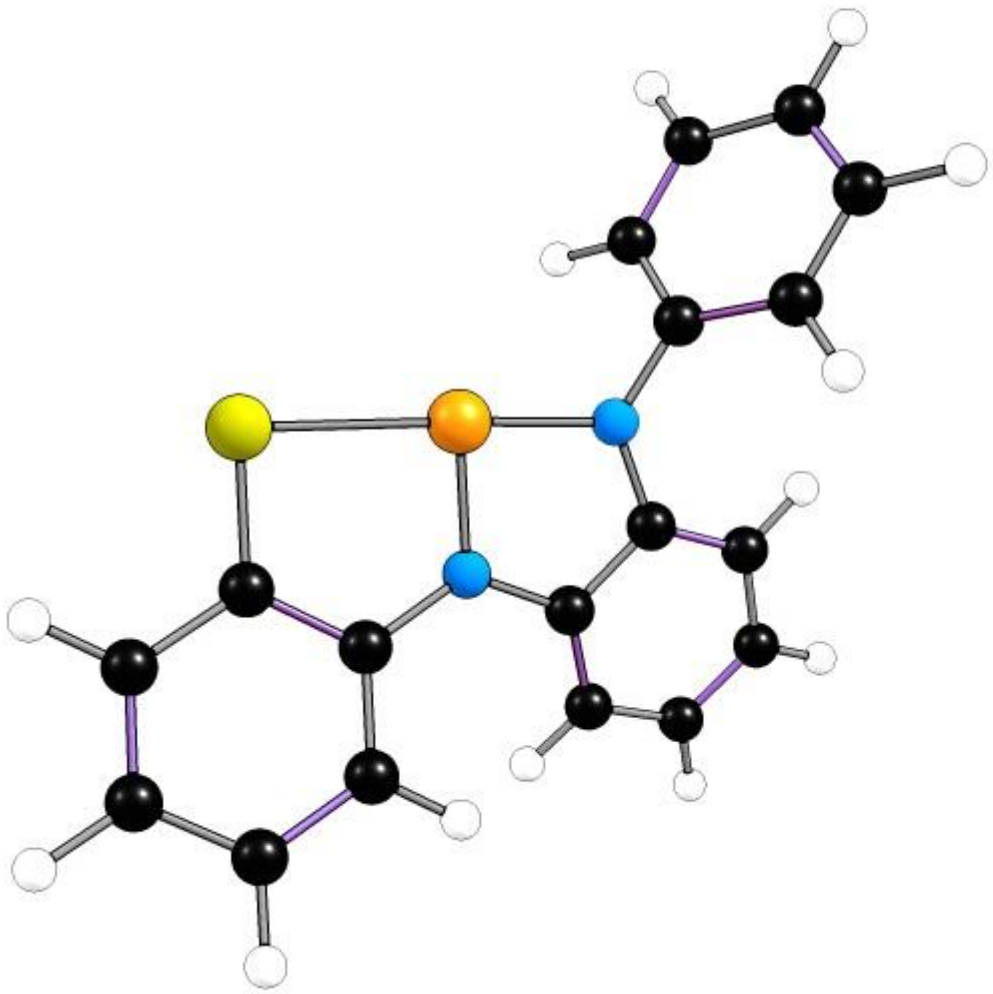

2+BCF

SCF = -3028.29300710

H(0 K) = -3027.886110

H(298 K) = -3027.835251

G(298 K) = -3027.976808

SCF+D3 = -3028.40376019

BS2 (def2-tzvp) = -3752.41517351

Low Freq. = 8.1743 cm^{-1}, 9.8472 cm^{-1}

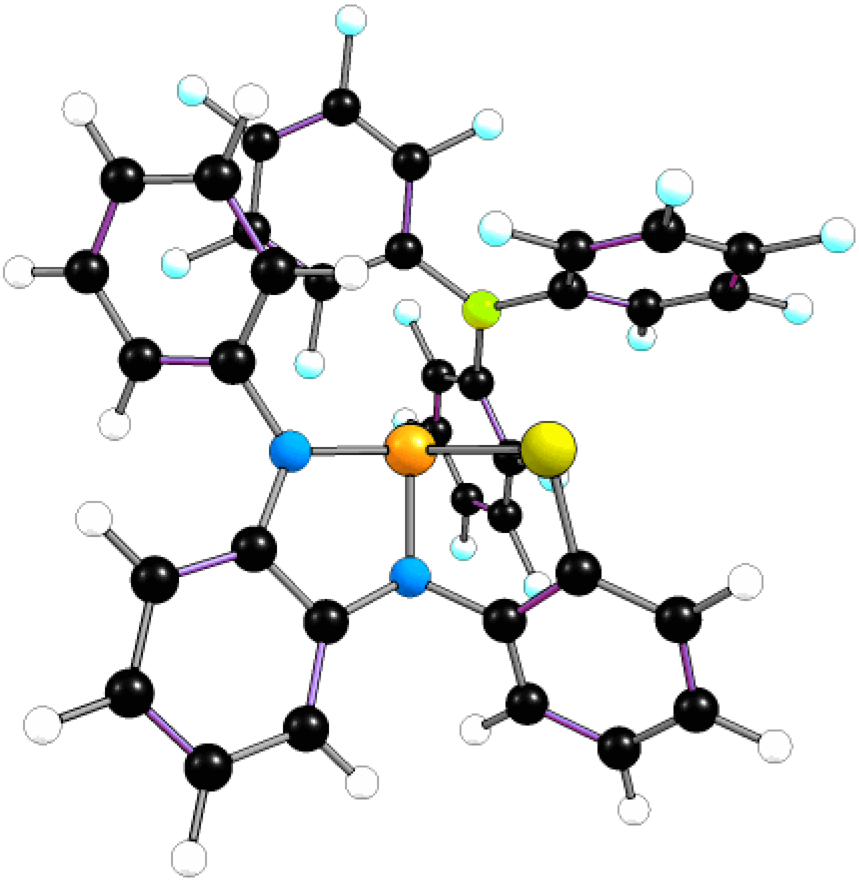

4TS

SCF = -3028.28221008

H(0 K) = -3027.875004

H(298 K) = -3027.825761

G(298 K) = -3027.957887

SCF+D3 = -3028.40865533

BS2 (def2-tzvp) = -3752.40684135

Low Freq. = -16.5033 cm^{-1}, 17.0010 cm^{-1}

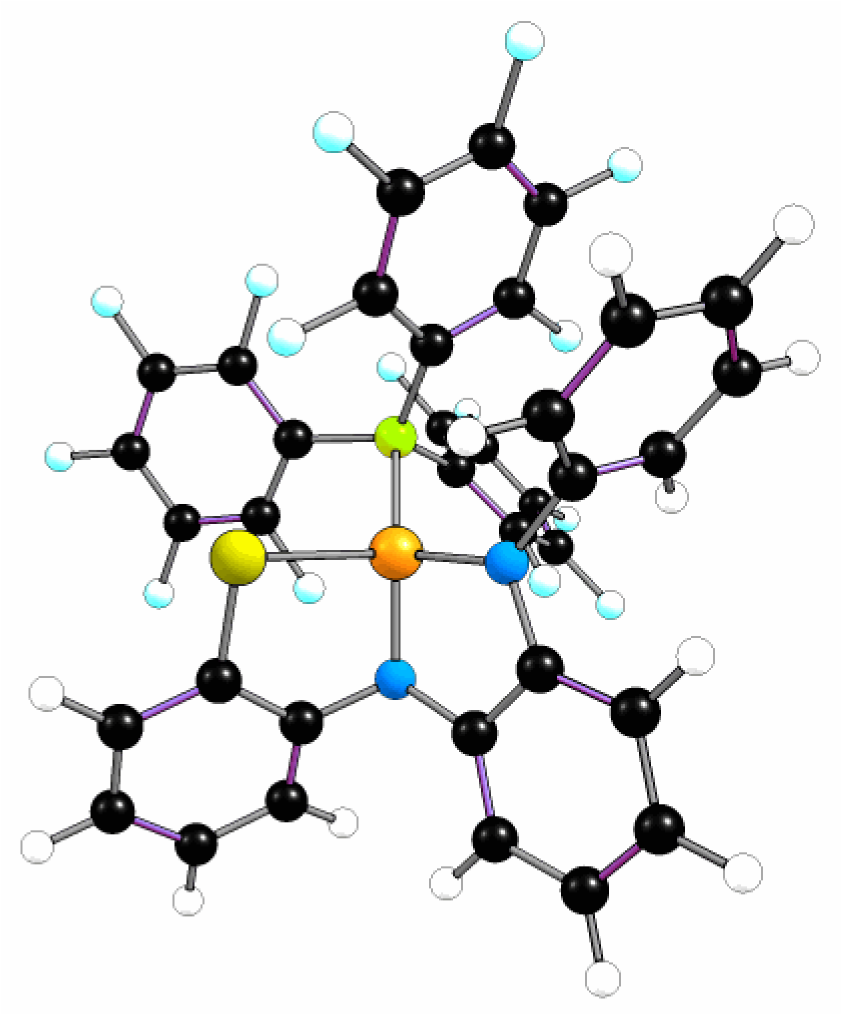

4a

SCF = -3028.28244132

H(0 K) = -3027.875064

H(298 K) = -3027.825037

G(298 K) = -3027.959842

SCF+D3 = -3028.41006291

BS2 (def2-tzvp) = -3752.40673248

Low Freq. = 12.6549 cm^{-1}, 16.7237 cm^{-1}

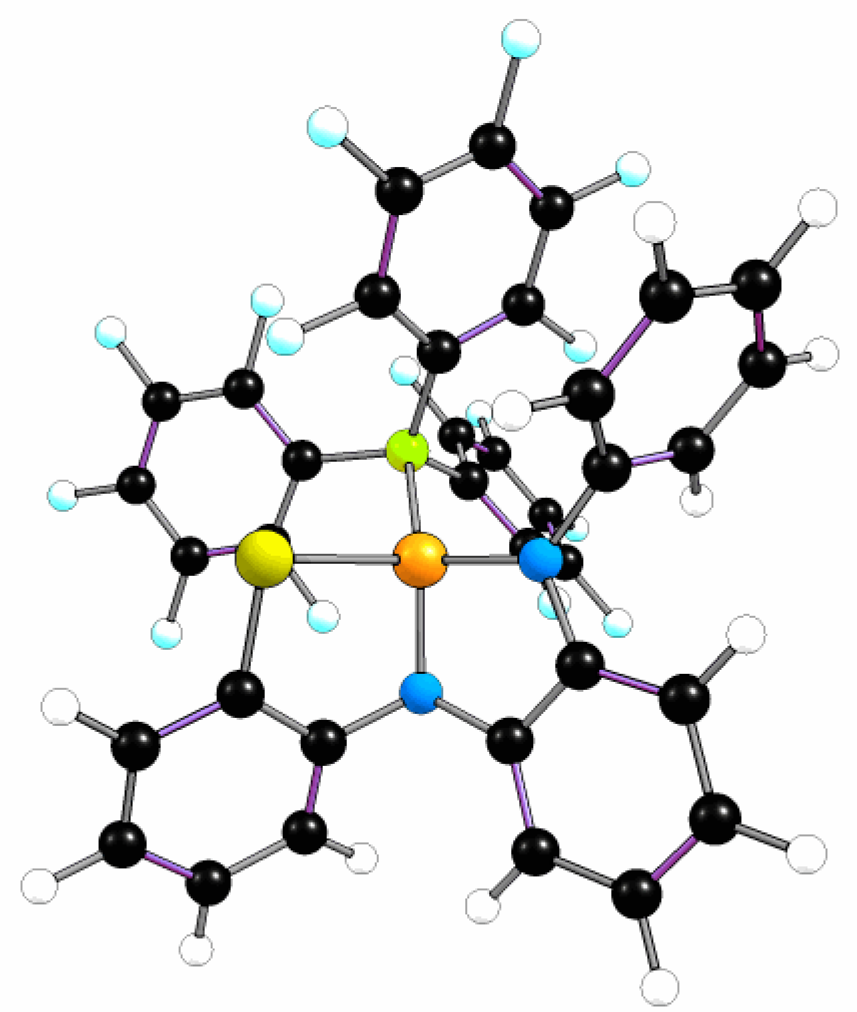

4b

SCF = -3028.29257600

H(0 K) = -3027.884738

H(298 K) = -3027.834716

G(298 K) = -3027.969608

SCF+D3 = -3028.40792543

BS2 (def2-tzvp) = -3752.41134094

Low Freq. = 10.8315 cm^{-1}, 20.4155 cm^{-1}

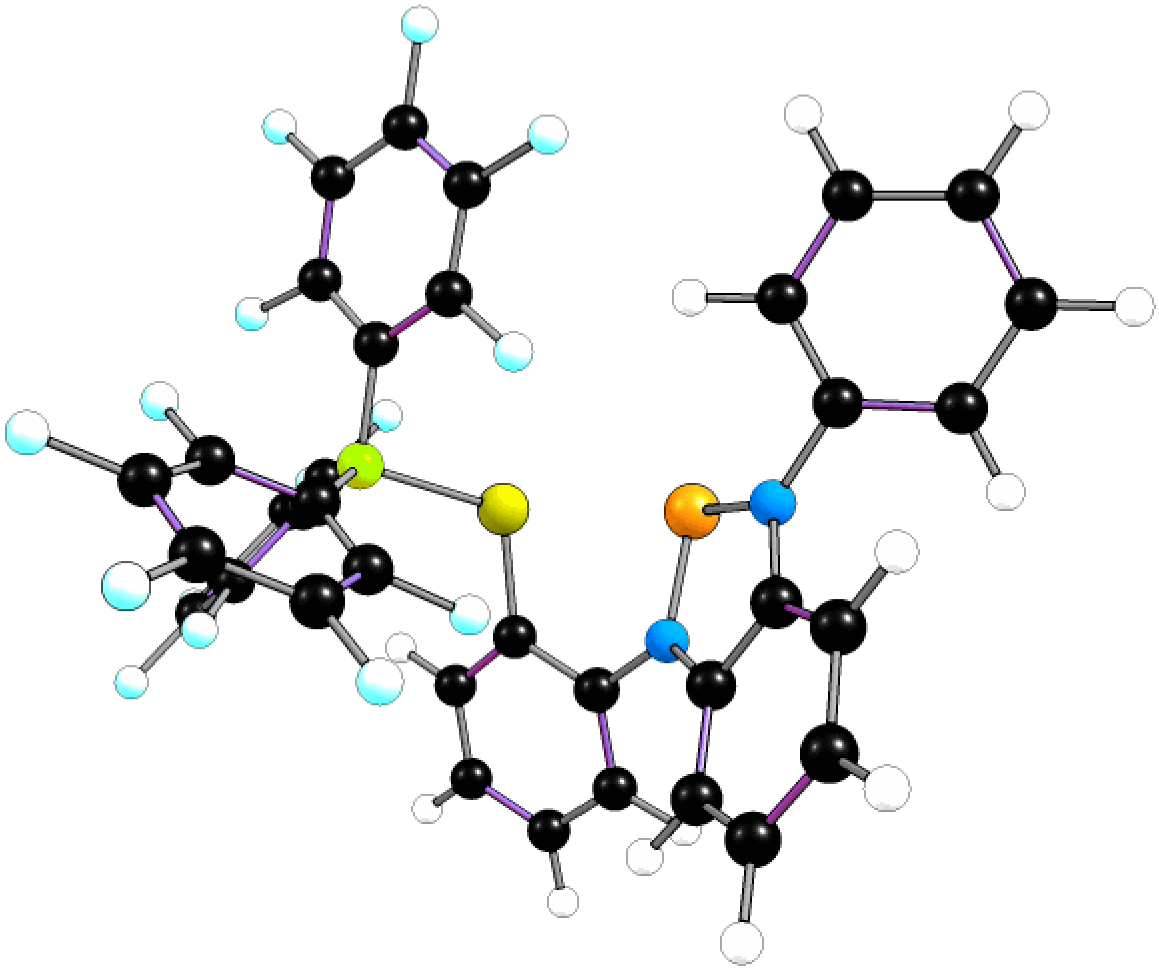

5a

SCF= -1222.21058813

H(0 K)= -1221.806088

H(298 K)= -1221.777321

G(298 K)= -1221.866934

SCF+D3= -1222.27970099

PCM SCF (Chloroform)= -1222.2185014

BS2 (def2-tzvp)= -1945.49931042

Low Freq. = 13.8693 cm^{-1}, 18.7071 cm^{-1}

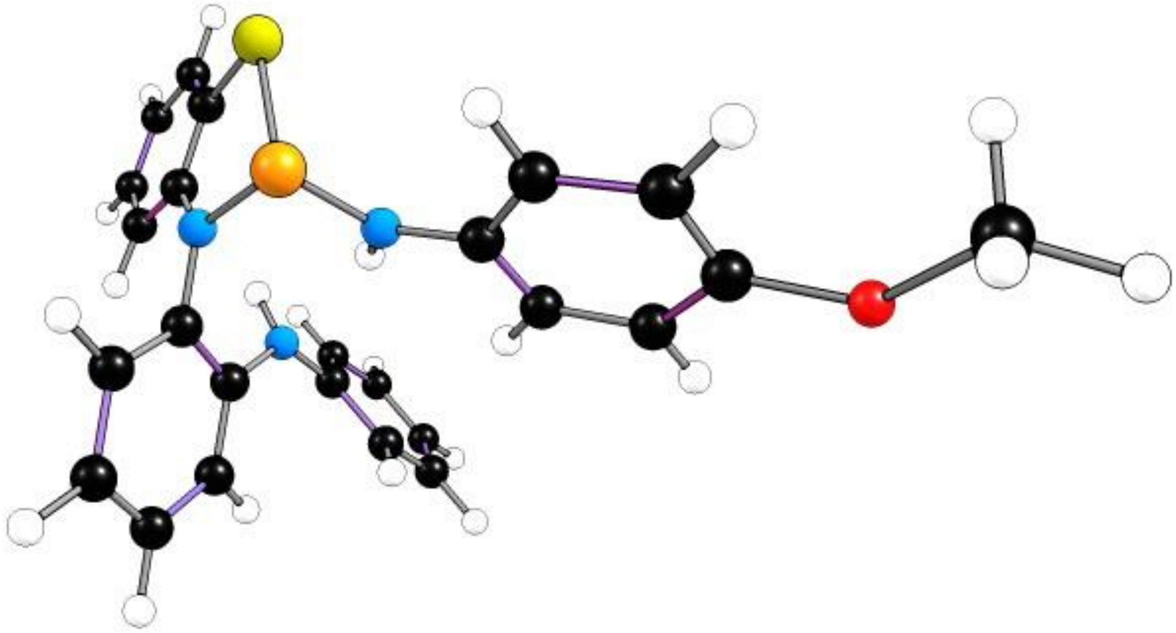

5b

SCF = -1222.21220576

H(0 K) = -1221.807738

H(298 K) = -1221.778745

G(298 K) = -1221.870912

SCF+D3 = -1222.27567429

PCM SCF (Chloroform) = -1222.2205307

BS2 (def2-tzvp) = -1945.50174495

Low Freq. = 6.5336 cm^{-1}, 12.6684 cm^{-1}

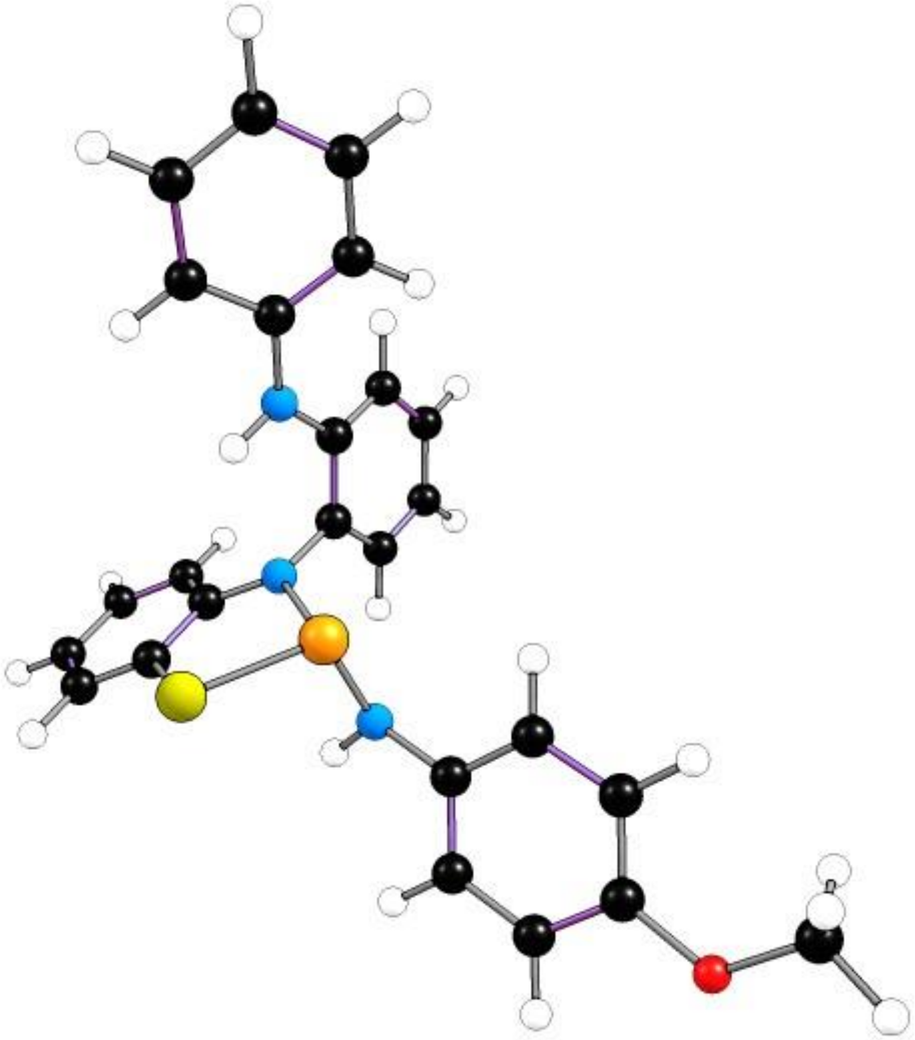

5c

SCF = -1222.19045529

H(0 K) = -1221.786336

H(298 K) = -1221.758395

G(298 K) = -1221.845304

SCF+D3 = -1222.25972878

PCM SCF (Chloroform) = -1222.19849287

BS2 (def2-tzvp) = -1945.49322154

Low Freq. = 14.3052 cm^{-1}, 20.8696 cm^{-1}

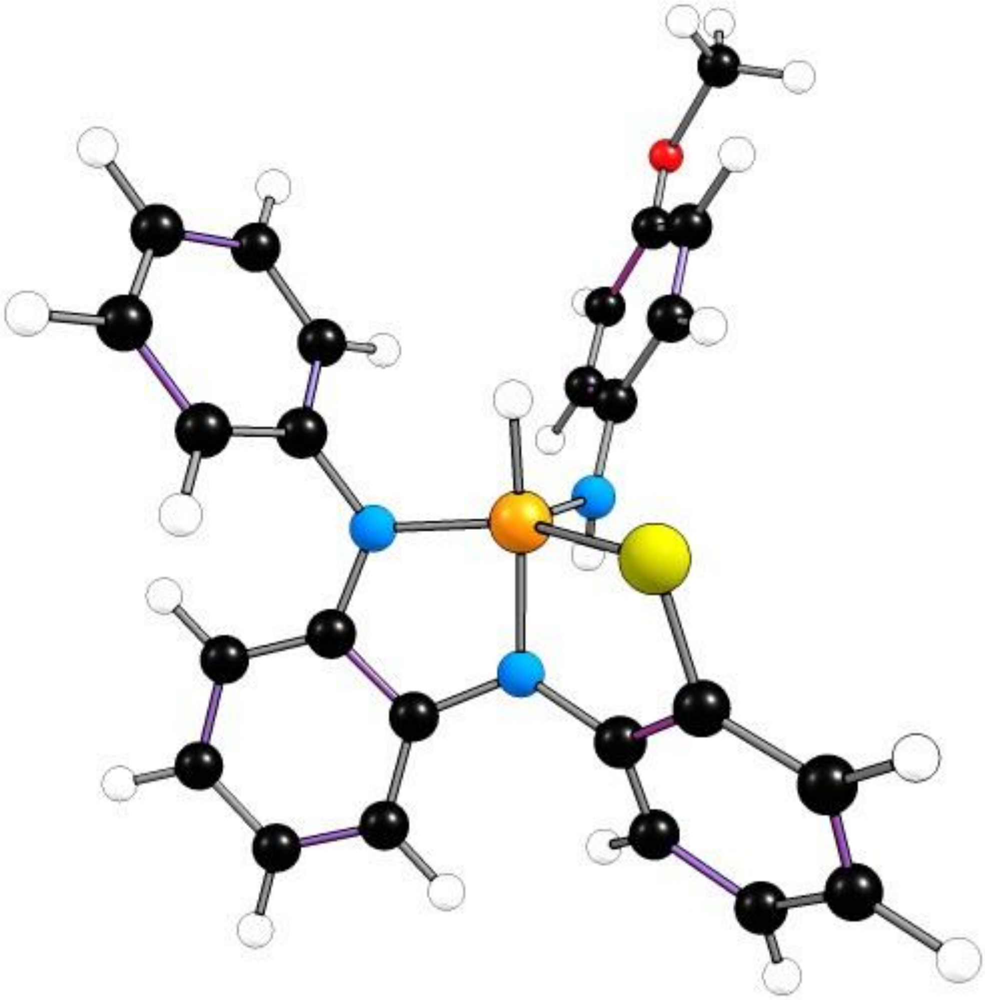

6a

SCF = -876.642254867

H(0 K) = -876.348176

H(298 K) = -876.326543

G(298 K) = -876.398546

SCF+D3 = -876.691533617

PCM SCF (THF) = -876.650405160

BS2 (def2-tzvp) = -1599.80850208

Low Freq. = 21.3200 cm^{-1}, 32.1261 cm^{-1}

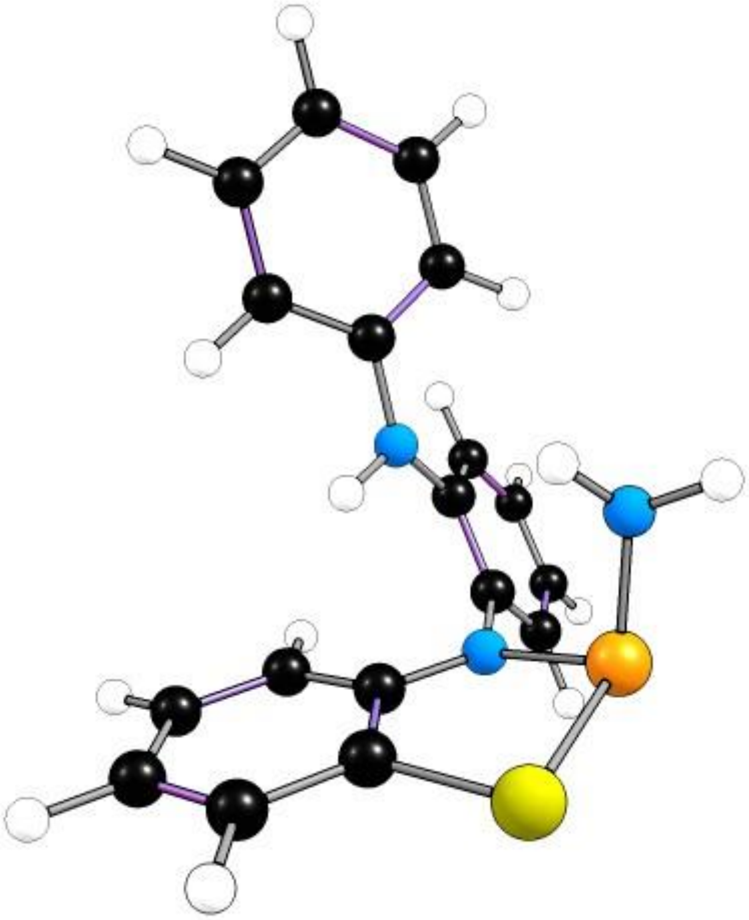

6b

SCF = -876.644239940

H(0 K) = -876.349872

H(298 K) = -876.328258

G(298 K) = -876.401500

SCF+D3 = -876.69101818

PCM SCF (THF) = -876.652650643

BS2 (def2-tzvp) = -1599.81043320

Low Freq. = 9.1439 cm^{-1}, 29.1774 cm^{-1}

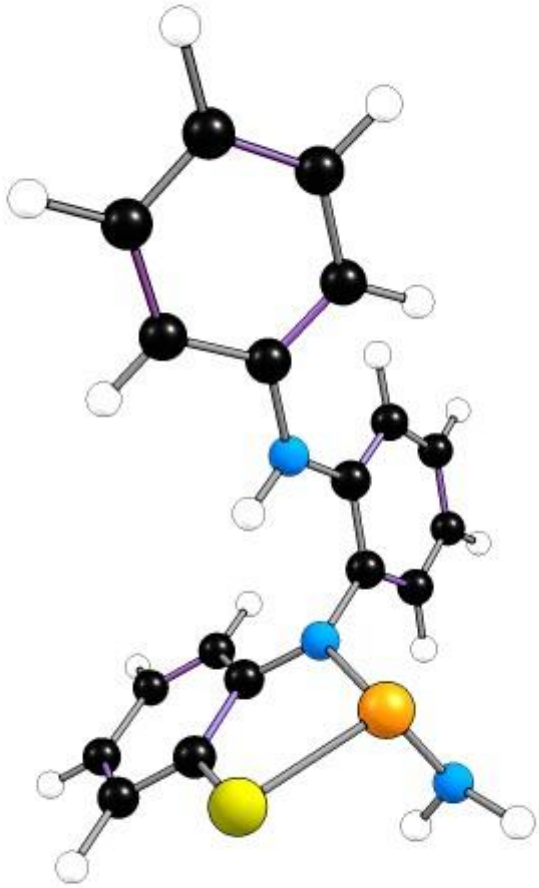

6c

SCF = -876.624564656

H(0 K) = -876.330146

H(298 K) = -876.309755

G(298 K) = -876.378066

SCF+D3 = -876.673889686

PCM SCF (THF) = -876.632662432

BS2 (def2-tzvp) = -1599.80349804

Low Freq. = 27.0349 cm^{-1}, 41.6886 cm^{-1}

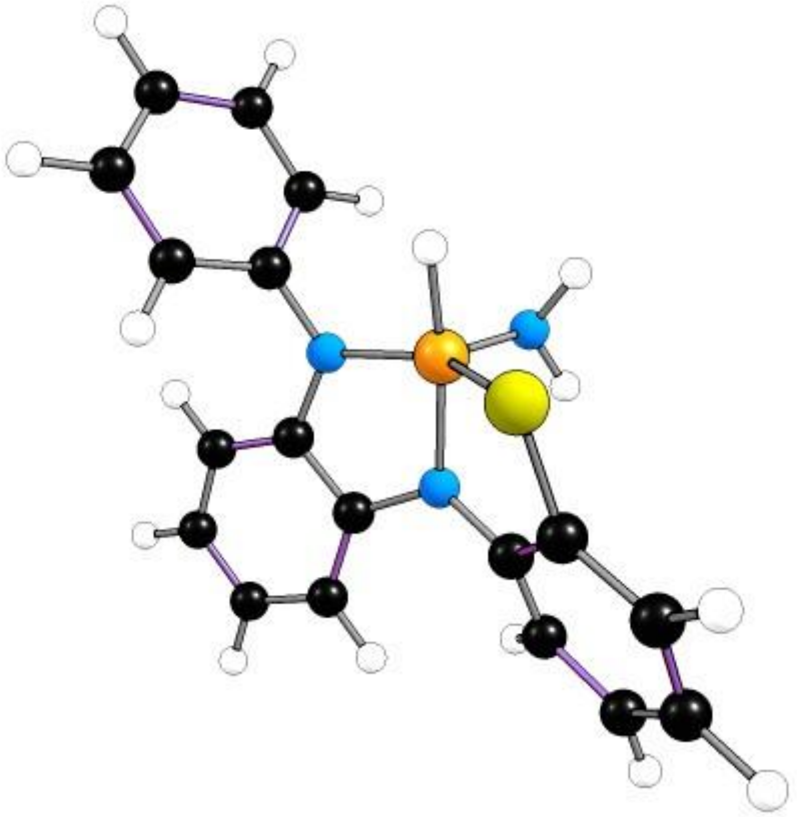

6TScoop

SCF = -876.613025597

H(0 K) = -876.321372

H(298 K) = -876.300979

G(298 K) = -876.369103

SCF+D3 = -876.662737297

PCM SCF (THF) = -876.623392387

BS2 (def2-tzvp) = -1599.77102424

Low Freq. = -363.1052 cm^{-1}, 31.7094 cm^{-1}

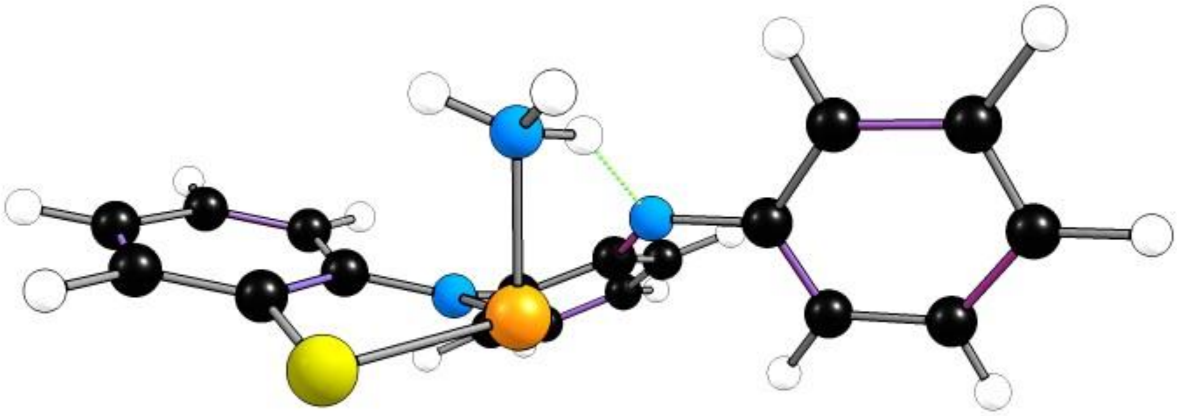

6TSox

SCF = -876.548497044

H(0 K) = -876.260059

H(298 K) = -876.239358

G(298 K) = -876.307587

SCF+D3 = -876.597497794

PCM SCF (THF) = -876.557106725

BS2 (def2-tzvp) = -1599.71628811

Low Freq. = -1457.1915 cm^{-1}, 34.7943 cm^{-1}

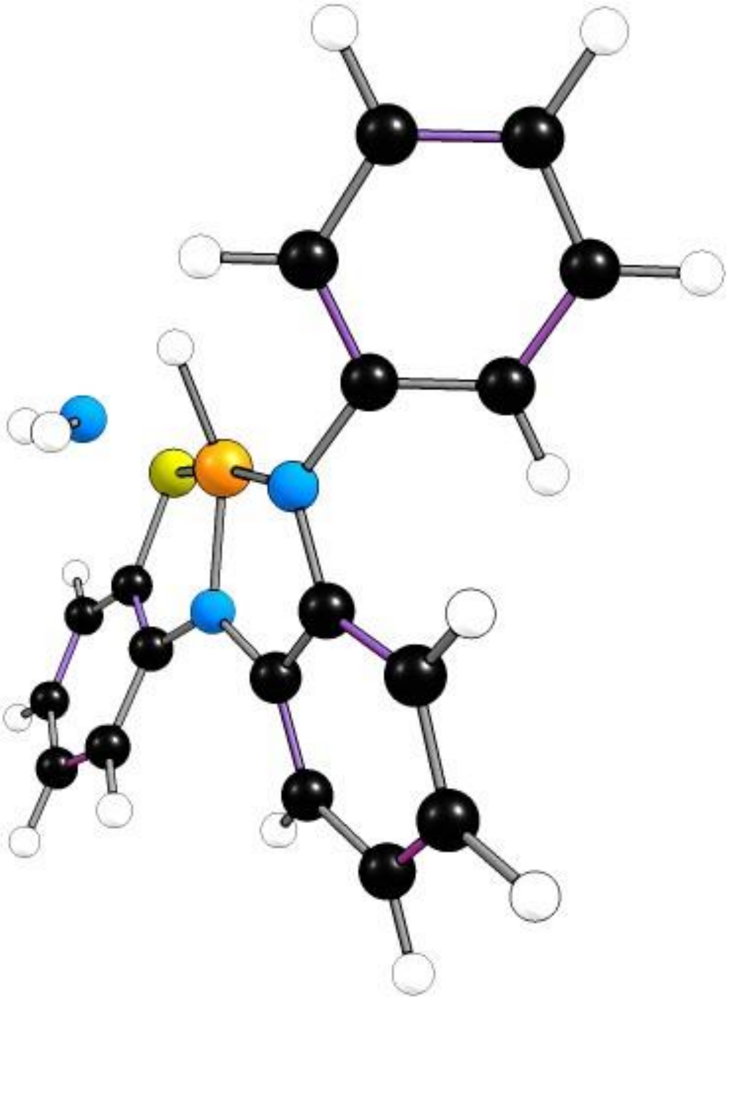

6cto6aTS

SCF = -933.164450155

H(0 K) = -932.838072

H(298 K) = -932.814759

G(298 K) = -932.888955

SCF+D3 = -933.220948415

PCM SCF (THF) = -933.179317191

BS2 (def2-tzvp) = -1656.35475395

Low Freq. = -605.3410 cm^{-1}, 24.9001 cm^{-1}

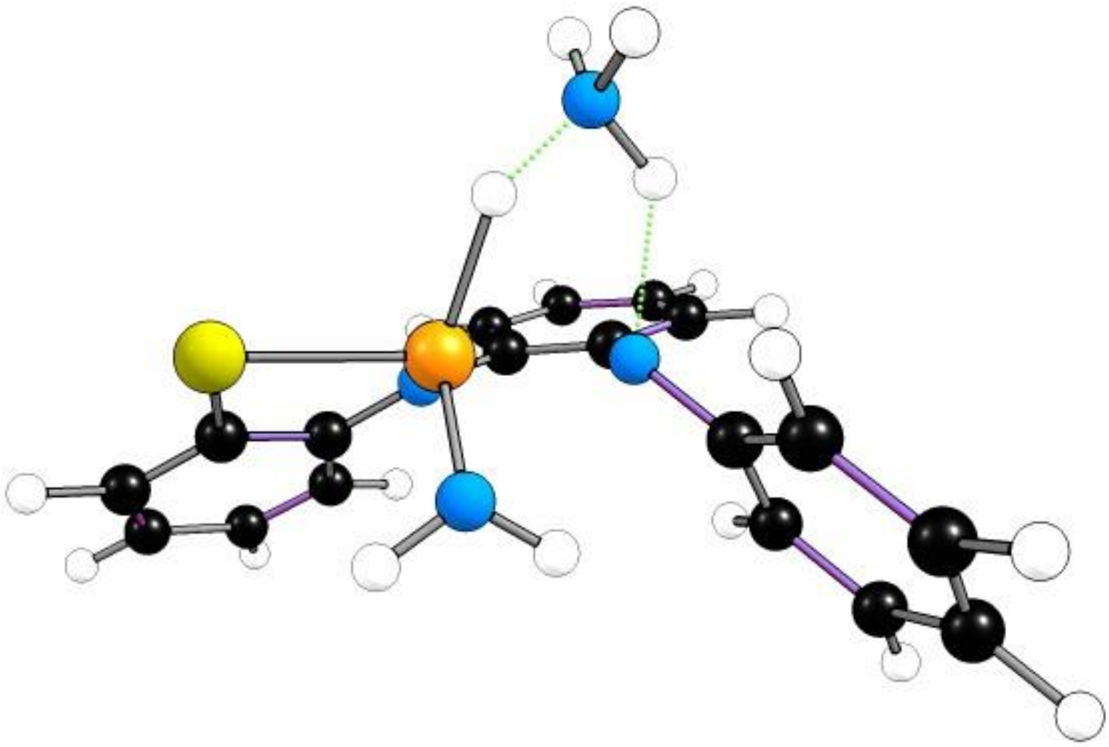

7a

SCF = -1033.87644315

H(0 K) = -1033.472063

H(298 K) = -1033.444643

G(298 K) = -1033.531004

SCF+D3 = -1033.94609725

PCM SCF (THF) = -1033.88271651

BS2 (def2-tzvp) = -1757.08866141

Low Freq. = 14.7525 cm^{-1}, 21.2315 cm^{-1}

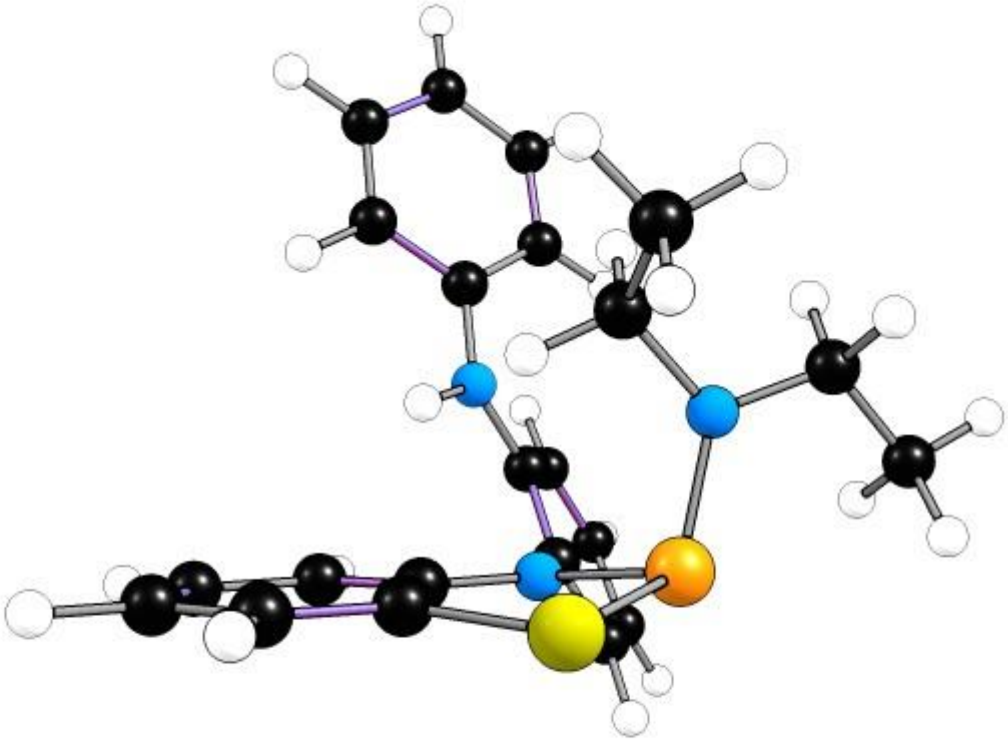

7b

SCF = -1033.87969489

H(0 K) = -1033.475164

H(298 K) = -1033.447823

G(298 K) = -1033.534638

SCF+D3 = -1033.94625522

PCM SCF (THF) = -1033.88664819

BS2 (def2-tzvp) = -1757.09170663

Low Freq. = 6.7616 cm^{-1}, 24.0332 cm^{-1}

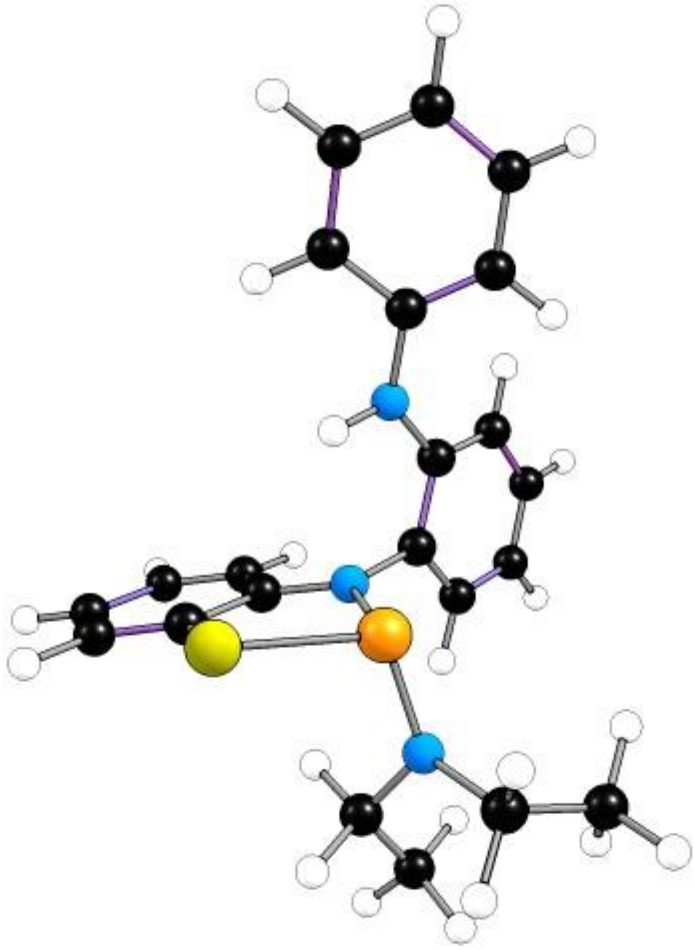

7c

SCF = -1033.85096894

H(0 K) = -1033.446930

H(298 K) = -1033.420634

G(298 K) = -1033.502503

SCF+D3 = -1033.92321612

PCM SCF (THF) = -1033.85702987

BS2 (def2-tzvp) = -1757.07476380

Low Freq. = 28.5781 cm^{-1}, 30.4861 cm^{-1}

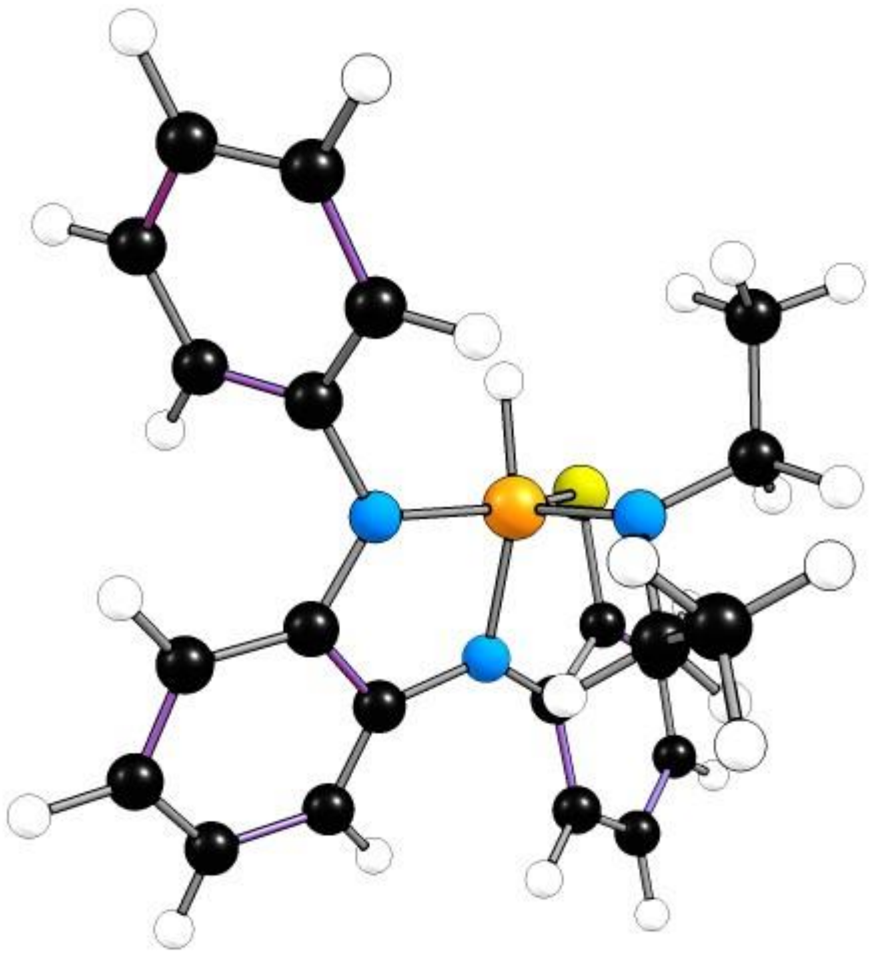

SH

SCF = -876.623986722

H(0 K) = -876.333227

H(298 K) = -876.311478

G(298 K) = -876.383872

SCF+D3 = -876.672743282

PCM SCF (THF) = -876.632975716

BS2 (def2-tzvp) = -1599.79397175

Low Freq. = 24.1123 cm^{-1}, 24.3662 cm^{-1}

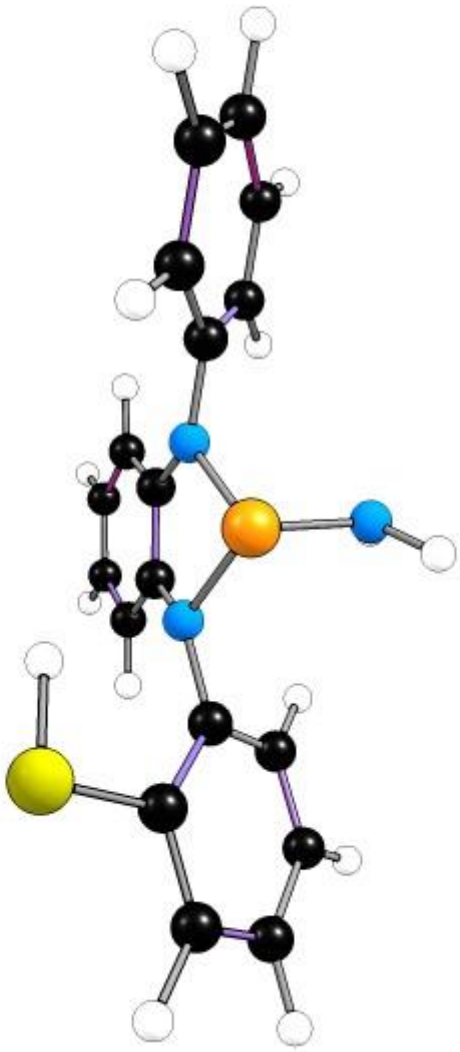

Coordinates of the computed species

4

NH3

N	0.00000	-0.00001	-0.12326
H	-0.30370	0.89070	0.28760
H	-0.61955	-0.70833	0.28762
H	0.92324	-0.18234	0.28762

18

pOMeNH2

N	-3.25555	-0.30341	-0.08195
H	-3.56787	-1.19702	0.29866
C	-1.86086	-0.11172	-0.00695
H	-3.79680	0.46185	0.32105
C	-1.30800	1.19152	-0.00498
C	0.07478	1.38661	-0.00050
C	0.95482	0.28516	0.00035
C	0.42054	-1.01632	-0.00228
C	-0.97196	-1.20562	-0.00495
H	-1.97514	2.06158	-0.01206
H	0.49861	2.39537	0.00411
O	2.30119	0.59055	0.00712
H	1.07312	-1.89315	0.00136
H	-1.37171	-2.22664	-0.01091
C	3.21017	-0.50569	0.00364
H	4.21818	-0.06482	0.00564
H	3.09180	-1.14386	0.90194
H	3.09215	-1.13742	-0.89913

16

Et2NH

N	0.00004	-0.28282	-0.08165
C	-1.22447	0.51926	0.02260
H	0.00030	-0.97247	0.68160
C	1.22448	0.51944	0.02219

C	2.47060	-0.36970	-0.02981
H	1.23482	1.22395	-0.83234
H	1.25382	1.15436	0.94255
H	-1.23477	1.22427	-0.83155
C	-2.47064	-0.36963	-0.02998
H	-1.25386	1.15382	0.94322
H	3.39107	0.23690	0.00064
H	2.50159	-1.06290	0.83044
H	2.47664	-0.97429	-0.95158
H	-3.39101	0.23708	0.00116
H	-2.47698	-0.97350	-0.95223
H	-2.50172	-1.06361	0.82965

34

BCF

C	2.02643	-1.48536	0.72381
C	0.82089	-1.33962	0.00023
C	0.40460	-2.48036	-0.72338
C	1.12886	-3.67868	-0.74503
C	2.31493	-3.77514	0.00039
C	2.76642	-2.67404	0.74565
B	-0.00043	-0.00014	-0.00006
C	-1.57111	-0.04138	-0.00007
C	-2.35041	0.89010	-0.72339
C	-3.75033	0.86256	-0.74522
C	-4.42736	-0.11635	-0.00019
C	-3.69997	-1.05834	0.74496
C	-2.30055	-1.01238	0.72332
F	-1.75218	1.84480	-1.46943
F	-4.44920	1.75778	-1.46578
F	-5.76736	-0.15156	-0.00028
F	-4.35100	-1.98896	1.46549
F	-1.65311	-1.93420	1.46959

F	-0.72088	-2.43993	-1.47009
F	0.70371	-4.73163	-1.46584
F	3.01586	-4.91775	0.00047
F	3.89776	-2.77203	1.46640
F	2.50059	-0.46345	1.46999
C	0.74925	1.38071	-0.00028
C	0.27381	2.49764	0.72404
C	0.93397	3.73243	0.74586
C	2.11288	3.89157	-0.00017
C	2.62135	2.81611	-0.74617
C	1.94505	1.59009	-0.72444
F	-0.84760	2.39767	1.47124
F	2.47198	0.59498	-1.47143
F	3.74553	2.97391	-1.46751
F	2.75265	5.06952	-0.00011
F	0.45435	4.76121	1.46738

35

2

C	-2.44942	-0.17868	-0.54227
C	-2.61557	-1.26025	0.36832
C	-3.55934	0.44839	-1.12535
C	-3.92269	-1.65373	0.71482
C	-4.85738	0.04468	-0.76875
H	-3.39806	1.23984	-1.86361
C	-5.03140	-0.99681	0.15572
H	-4.06809	-2.48054	1.41610
H	-5.72399	0.53489	-1.22238
H	-6.03995	-1.31920	0.43479
C	-0.58680	1.42383	-0.21960
C	-1.29001	2.60629	0.05045
C	0.78622	1.31536	0.11294
C	-0.60859	3.67498	0.65744

H	-2.35433	2.67953	-0.18591
C	1.45875	2.37785	0.73641
C	0.75126	3.56378	0.99456
H	-1.15197	4.59934	0.87613
H	2.50895	2.27460	1.02123
H	1.26755	4.40191	1.47247
C	2.66610	-0.34206	-0.16187
C	3.67848	0.49155	-0.68546
C	3.00617	-1.59971	0.37732
C	5.01301	0.06634	-0.65548
H	3.41005	1.45396	-1.13017
C	4.34347	-2.02516	0.37830
H	2.22397	-2.22883	0.81616
C	5.35214	-1.19258	-0.12936
H	5.79215	0.71815	-1.06410
H	4.59509	-3.00555	0.79521
H	6.39617	-1.52040	-0.11596
S	-1.16374	-2.09289	0.99678
N	-1.10236	0.23730	-0.80948
N	1.29711	0.04396	-0.22666
P	0.06618	-1.09431	-0.76629

35

2TS

C	2.48457	-0.02393	0.09942
C	2.99265	-1.35636	-0.07878
C	3.36710	0.99416	0.53819
C	4.39036	-1.56788	0.02742
C	4.73395	0.74292	0.67022
H	2.97566	1.96657	0.83610
C	5.25597	-0.53297	0.37836
H	4.76880	-2.58064	-0.14067
H	5.39228	1.54335	1.02167

H	6.33033	-0.72395	0.46590
C	0.42787	1.44198	-0.16159
C	1.00536	2.70119	-0.44035
C	-1.00474	1.33200	-0.08353
C	0.18912	3.83249	-0.53420
H	2.07261	2.79292	-0.63939
C	-1.81127	2.49251	-0.17115
C	-1.21004	3.73349	-0.37661
H	0.64711	4.80072	-0.75741
H	-2.89821	2.39178	-0.11285
H	-1.83256	4.63053	-0.45395
C	-2.77653	-0.40924	0.06997
C	-3.60457	0.03423	1.12586
C	-3.26976	-1.35463	-0.85474
C	-4.91445	-0.45047	1.23666
H	-3.20581	0.74038	1.86072
C	-4.57694	-1.84679	-0.72169
H	-2.62824	-1.67740	-1.67996
C	-5.40510	-1.39387	0.31711
H	-5.55102	-0.09890	2.05536
H	-4.95161	-2.57939	-1.44392
H	-6.42743	-1.77323	0.41151
S	1.87804	-2.69834	-0.30971
N	1.08721	0.19088	-0.07018
N	-1.43057	0.02046	-0.03022
P	0.00144	-1.19678	-0.13390

69

2+BCF

C	0.59010	-2.19124	1.78476
C	-0.78778	-1.94803	1.59422
C	-1.60032	-2.13760	2.73390
C	-1.09476	-2.54526	3.97499

C	0.28531	-2.76048	4.11470
C	1.13816	-2.57483	3.01487
B	-1.38105	-1.49955	0.20617
C	-0.83257	-2.13150	-1.12252
C	-0.39202	-3.47394	-1.19788
C	0.11870	-4.04817	-2.36799
C	0.22565	-3.26320	-3.52754
C	-0.19616	-1.92549	-3.50682
C	-0.72344	-1.39609	-2.32431
P	1.22220	1.19904	-0.33690
S	2.35724	0.57944	-2.32003
C	3.73121	0.04924	-1.30824
C	3.78074	0.45418	0.05522
C	4.78565	-0.02133	0.90946
C	5.79235	-0.86175	0.40502
C	5.77512	-1.23263	-0.94833
C	4.74890	-0.79107	-1.79985
N	2.77803	1.38300	0.48957
C	3.11439	2.74793	0.70815
C	4.32483	3.25155	1.20529
C	4.47697	4.64265	1.33407
C	3.44033	5.51801	0.96771
C	2.23003	5.02057	0.45707
C	2.06892	3.63111	0.34394
N	0.94804	2.92673	-0.14542
C	-0.29982	3.52168	-0.49474
C	-0.95994	4.38431	0.40695
C	-2.20185	4.93278	0.06048
C	-2.80475	4.61610	-1.16988
C	-2.15243	3.74783	-2.05882
C	-0.89805	3.20939	-1.73194
C	-2.54274	-0.43992	0.17084

C	-3.61823	-0.52497	-0.74128
C	-4.67448	0.39442	-0.76567
C	-4.67063	1.46617	0.14065
C	-3.62218	1.59928	1.06414
C	-2.59185	0.65179	1.06687
H	4.76432	0.25861	1.96694
H	4.72889	-1.10516	-2.84757
H	6.57672	-1.23183	1.07194
H	6.55552	-1.88888	-1.34726
H	5.14035	2.57172	1.46274
H	5.42047	5.04293	1.71752
H	1.42872	5.69555	0.14549
H	3.57457	6.59922	1.06937
H	-0.50474	4.60181	1.37734
H	-0.36974	2.56299	-2.43960
H	-2.71000	5.59826	0.76543
H	-2.60966	3.49857	-3.02170
H	-3.77708	5.04454	-1.43180
F	-0.48308	-4.28352	-0.11933
F	0.50828	-5.33578	-2.39351
F	0.73393	-3.79047	-4.65063
F	-0.07376	-1.16562	-4.60948
F	-1.09459	-0.09357	-2.36411
F	-3.68406	-1.54594	-1.62467
F	-5.68634	0.26351	-1.64369
F	-5.66903	2.36229	0.12457
F	-3.61936	2.62901	1.93011
F	-1.59504	0.84525	1.96073
F	-2.93832	-1.95893	2.65423
F	-1.91195	-2.73211	5.02764
F	0.78931	-3.14087	5.29830
F	2.46173	-2.77104	3.15386

F	1.45955	-2.01599	0.76268

69

4TS

P	-0.47994	1.12679	-0.54675
S	-1.50378	0.82715	-2.54095
N	0.68976	2.40694	-0.43238
N	-1.76224	2.18757	0.04510
C	0.10312	3.63918	-0.05559
C	0.75722	4.87549	0.04140
H	1.82108	4.95436	-0.19520
C	0.01527	5.99556	0.45078
H	0.51470	6.96489	0.54055
C	-1.35436	5.87981	0.73745
H	-1.92652	6.76011	1.04507
C	-2.00977	4.64035	0.63357
H	-3.07865	4.55954	0.83855
C	-1.27217	3.51743	0.23673
C	-3.03982	1.99554	-0.57289
C	-4.24611	2.34733	0.05039
H	-4.23658	2.68507	1.09012
C	-5.45389	2.23212	-0.65712
H	-6.39345	2.51037	-0.17079
C	-5.45466	1.74385	-1.97272
H	-6.39550	1.64969	-2.52385
C	-4.25668	1.33774	-2.58350
H	-4.26503	0.91972	-3.59446
C	-3.04380	1.45270	-1.88203
C	2.11024	2.32849	-0.65415
C	2.97431	2.35545	0.45746
H	2.54521	2.35384	1.46296
C	4.36119	2.38126	0.25230
H	5.03535	2.40052	1.11426

C	4.88308	2.37214	-1.05300
H	5.96627	2.38948	-1.20920
C	4.01550	2.33872	-2.15696
H	4.41925	2.33316	-3.17430
C	2.62457	2.32745	-1.96256
H	1.93552	2.33136	-2.81301
B	0.21310	-0.88681	0.20761
C	1.68022	-0.99365	-0.47894
C	2.88164	-1.08407	0.25703
C	4.13580	-1.30836	-0.33437
C	4.23711	-1.43312	-1.72466
C	3.07934	-1.32037	-2.50767
C	1.85305	-1.09998	-1.87440
C	0.17865	-0.88382	1.83800
C	0.13425	0.21343	2.70875
C	0.08216	0.10663	4.10660
C	0.09852	-1.16286	4.69811
C	0.17253	-2.29706	3.87739
C	0.21676	-2.13659	2.48509
C	-0.95446	-1.85996	-0.36919
C	-0.75541	-3.03356	-1.12259
C	-1.80489	-3.87196	-1.53148
C	-3.12305	-3.56785	-1.16571
C	-3.37401	-2.42715	-0.39003
C	-2.29347	-1.62519	-0.00568
F	2.89730	-0.95205	1.60341
F	5.24423	-1.38499	0.42789
F	5.43309	-1.62947	-2.30515
F	3.16123	-1.40685	-3.85003
F	0.77523	-0.97066	-2.69558
F	0.14946	1.48681	2.21831
F	0.02798	1.21120	4.87793

F	0.05452	-1.29151	6.03629
F	0.21235	-3.52528	4.42953
F	0.33610	-3.27359	1.75514
F	0.48550	-3.44165	-1.47521
F	-1.55104	-4.97994	-2.25505
F	-4.13618	-4.36362	-1.55045
F	-4.63388	-2.12054	-0.02347
F	-2.58789	-0.52744	0.74688

69

4a

P	-0.55433	1.12289	-0.50666
S	-1.55103	0.80321	-2.50941
N	0.58198	2.42803	-0.34763
N	-1.87148	2.14607	0.07461
C	-0.04078	3.64064	0.03538
C	0.58090	4.89108	0.16049
H	1.64666	4.99898	-0.05499
C	-0.19535	5.98727	0.57053
H	0.27867	6.96696	0.68258
C	-1.56697	5.83472	0.82940
H	-2.16596	6.69671	1.13782
C	-2.19031	4.58178	0.69609
H	-3.26074	4.47310	0.87840
C	-1.41828	3.48218	0.29850
C	-3.13670	1.92634	-0.55570
C	-4.35876	2.24230	0.05639
H	-4.36996	2.57267	1.09841
C	-5.55491	2.09974	-0.66562
H	-6.50688	2.34977	-0.18818
C	-5.52827	1.62023	-1.98429
H	-6.46016	1.50505	-2.54650
C	-4.31340	1.24962	-2.58409

H	-4.29928	0.83748	-3.59737
C	-3.11227	1.39208	-1.86769
C	2.00484	2.39120	-0.56288
C	2.86019	2.45793	0.55343
H	2.42537	2.45893	1.55645
C	4.24724	2.51809	0.35678
H	4.91490	2.56552	1.22251
C	4.77745	2.50445	-0.94469
H	5.86075	2.54663	-1.09448
C	3.91793	2.43388	-2.05380
H	4.32870	2.42728	-3.06832
C	2.52687	2.38863	-1.86851
H	1.84358	2.36375	-2.72320
B	0.21986	-0.84359	0.16584
C	1.63143	-0.89689	-0.64508
C	2.89006	-0.83323	-0.00672
C	4.11150	-0.99280	-0.68000
C	4.11984	-1.21151	-2.06237
C	2.90103	-1.25685	-2.75298
C	1.70779	-1.09538	-2.04055
C	0.33061	-0.87792	1.79438
C	0.14511	0.16067	2.71592
C	0.20955	-0.00655	4.10831
C	0.48830	-1.27333	4.63606
C	0.70356	-2.34649	3.75913
C	0.62512	-2.12780	2.37711
C	-0.94871	-1.87770	-0.30052
C	-0.74326	-3.09068	-0.98688
C	-1.77990	-3.99028	-1.28430
C	-3.08799	-3.70959	-0.86743
C	-3.34158	-2.53105	-0.15135
C	-2.27343	-1.66985	0.12422

F	2.99526	-0.59651	1.32157
F	5.27584	-0.91591	-0.00648
F	5.28198	-1.34864	-2.72321
F	2.88867	-1.44293	-4.08765
F	0.57085	-1.13540	-2.78426
F	-0.09981	1.43281	2.28953
F	0.01395	1.04138	4.93415
F	0.55821	-1.45670	5.96694
F	0.99038	-3.56852	4.24860
F	0.87731	-3.19673	1.58039
F	0.49217	-3.47535	-1.38073
F	-1.52162	-5.13309	-1.94987
F	-4.08863	-4.56375	-1.14551
F	-4.59075	-2.24664	0.26682
F	-2.56690	-0.54377	0.83517

69

4b

P	2.49394	-0.43183	-1.90178
S	-0.16750	-0.35609	-1.50227
N	3.97316	-0.29199	-0.97646
N	2.34168	-2.06975	-1.31423
C	4.27823	-1.40813	-0.19203
C	5.32389	-1.54370	0.74043
H	6.02657	-0.72242	0.90085
C	5.42775	-2.74457	1.44799
H	6.23834	-2.87448	2.17095
C	4.48524	-3.77845	1.25727
H	4.56401	-4.69758	1.84526
C	3.43268	-3.64479	0.34585
H	2.68254	-4.42992	0.24024
C	3.33553	-2.45194	-0.39753
C	1.15295	-2.81249	-1.58174

C	1.22940	-4.19244	-1.85421
H	2.21128	-4.66747	-1.93233
C	0.06197	-4.93765	-2.05612
H	0.13239	-6.00838	-2.26953
C	-1.18708	-4.29778	-2.01472
H	-2.10524	-4.86814	-2.18586
C	-1.26574	-2.91487	-1.80546
H	-2.23402	-2.41210	-1.83636
C	-0.10523	-2.14085	-1.59088
C	4.79245	0.88620	-1.06965
C	6.14272	0.76398	-1.45659
H	6.56177	-0.22521	-1.66384
C	6.92507	1.91856	-1.59304
H	7.97314	1.82515	-1.89466
C	6.36540	3.18802	-1.36488
H	6.98031	4.08600	-1.47986
C	5.01824	3.29974	-0.99010
H	4.57174	4.28125	-0.80535
C	4.22652	2.15117	-0.83074
H	3.18947	2.23373	-0.49639
B	-1.43512	0.32563	0.06080
C	-0.99417	-0.09187	1.57742
C	-1.20316	1.94507	-0.08756
C	-2.86120	-0.26705	-0.45293
C	-1.75729	0.47028	2.62155
C	0.08315	-0.87771	2.00904
C	0.08349	2.49732	0.05338
C	-2.22426	2.90623	-0.23130
C	-3.60390	-1.25501	0.22637
C	-3.41967	0.13100	-1.68885
C	-1.49042	0.27980	3.98317
F	-2.83681	1.23694	2.31802

C	0.39263	-1.09890	3.36202
F	0.90903	-1.49292	1.11949
C	0.37416	3.86522	-0.01406
F	1.15528	1.67696	0.28142
C	-1.98392	4.28945	-0.29658
F	-3.52421	2.54246	-0.29398
C	-4.80663	-1.79607	-0.26192
F	-3.17988	-1.77235	1.40482
C	-4.60971	-0.38603	-2.21565
F	-2.78599	1.05412	-2.45326
C	-0.39771	-0.51519	4.35844
F	-2.26866	0.84530	4.92868
F	1.44798	-1.86933	3.70540
C	-0.67398	4.77643	-0.19494
F	1.64907	4.30805	0.10672
F	-3.00649	5.15457	-0.44516
C	-5.31461	-1.35980	-1.49125
F	-5.46697	-2.74013	0.44007
F	-5.08329	0.03938	-3.40432
F	-0.11503	-0.71661	5.66017
F	-0.42708	6.09773	-0.25831
F	-6.46099	-1.87089	-1.97648

53

5a

C	-5.56129	-3.41938	-1.85542
C	-4.65860	-3.90978	-0.89859
C	-3.73370	-3.05945	-0.27576
C	-3.71411	-1.68126	-0.59305
C	-4.61950	-1.19189	-1.56446
C	-5.52547	-2.05531	-2.19116
N	-2.79489	-0.77631	-0.03278
C	-2.15146	-0.79735	1.19803

C	-1.05549	0.10098	1.39395
C	-0.37543	0.13858	2.62242
C	-0.76502	-0.68967	3.68465
C	-1.85903	-1.55340	3.50575
C	-2.54835	-1.60990	2.28867
N	-0.67524	0.95686	0.30138
P	0.63244	0.37101	-0.75805
N	2.04683	0.83522	0.18719
C	-1.03406	2.32417	0.25811
C	-0.61376	3.03983	-0.89108
C	-0.92952	4.39894	-1.04049
C	-1.68675	5.04814	-0.05069
C	-2.11539	4.33743	1.08291
C	-1.79141	2.98138	1.24722
S	0.26926	2.08070	-2.12215
H	-2.11460	2.42803	2.13360
H	-0.59100	4.94046	-1.92951
H	-2.70288	4.84229	1.85649
H	-1.93746	6.10682	-0.16796
H	0.46942	0.82618	2.72286
H	-0.22606	-0.65860	4.63587
H	-3.41808	-2.26236	2.18506
H	-2.19256	-2.19154	4.33110
H	-3.00315	-3.46866	0.42629
H	-4.60924	-0.12504	-1.81534
H	-4.65708	-4.97462	-0.64181
H	-6.21612	-1.65445	-2.94060
H	-6.27767	-4.09104	-2.33814
C	3.29498	0.17405	0.01304
H	2.13524	1.83405	0.39808
H	-2.52146	0.01228	-0.62127
C	4.48840	0.91350	-0.15411

C	5.71735	0.26548	-0.29490
C	5.78392	-1.14363	-0.30123
C	4.60030	-1.89038	-0.14258
C	3.37210	-1.23230	0.02968
H	4.44860	2.00872	-0.17492
H	6.64357	0.83473	-0.41630
O	7.04002	-1.68122	-0.45946
H	4.62168	-2.98277	-0.12790
H	2.46284	-1.81933	0.19295
C	7.14179	-3.10437	-0.47784
H	8.20858	-3.32851	-0.62363
H	6.80301	-3.55303	0.47628
H	6.55952	-3.54685	-1.30935

53

5b

C	3.76397	-0.82947	-0.08957
C	2.54287	-1.54052	-0.12530
C	2.32510	-2.49206	-1.15635
C	3.28334	-2.67971	-2.16347
C	4.48622	-1.95205	-2.13393
C	4.72500	-1.04050	-1.09420
N	1.51516	-1.38069	0.82129
P	-0.06966	-2.17989	0.58366
S	0.83054	-3.45577	-1.01937
C	1.53636	-0.33072	1.80217
C	1.48620	1.04087	1.40773
C	1.48593	2.02992	2.41548
C	1.48505	1.67310	3.77055
C	1.51254	0.32340	4.15622
C	1.55245	-0.66862	3.16671
N	1.45040	1.34656	0.02726
C	1.14531	2.60715	-0.53471

C	0.07210	3.39655	-0.05819
C	-0.24397	4.60637	-0.69254
C	0.48093	5.04365	-1.81355
C	1.53467	4.25158	-2.29783
C	1.87197	3.04867	-1.66337
N	-0.89330	-0.83739	-0.16287
H	3.96459	-0.14276	0.73929
H	3.09247	-3.40693	-2.95893
H	5.66797	-0.48601	-1.05310
H	5.23477	-2.10954	-2.91627
H	1.59418	-1.72975	3.43178
H	1.51539	0.04500	5.21410
H	1.48629	3.08420	2.12636
H	1.47781	2.46221	4.52960
H	-0.51786	3.05168	0.79591
H	2.70399	2.44129	-2.03824
H	-1.07680	5.20587	-0.30991
H	2.10882	4.57453	-3.17261
H	0.22499	5.98688	-2.30545
H	-0.31377	-0.09593	-0.57320
C	-2.29190	-0.64802	-0.22885
H	2.11096	0.79089	-0.52755
C	-2.80714	0.63076	-0.55030
C	-4.18358	0.85418	-0.60463
C	-5.09077	-0.18987	-0.32758
C	-4.59035	-1.46690	-0.01273
C	-3.20422	-1.69288	0.01959
H	-2.11574	1.45644	-0.75432
H	-4.58173	1.84132	-0.85757
O	-6.42568	0.13996	-0.40331
H	-5.26510	-2.30252	0.18861
H	-2.83622	-2.70122	0.23651

C	-7.36621	-0.89638	-0.12969
H	-8.36047	-0.43729	-0.23230
H	-7.27671	-1.73390	-0.84902
H	-7.25316	-1.29381	0.89801

53

5c

C	1.64862	4.71439	1.46937
C	2.21876	3.77368	0.59825
C	1.43772	2.73320	0.06999
C	0.07965	2.62037	0.42917
C	-0.49005	3.55879	1.31693
C	0.29224	4.60497	1.82401
N	-0.69727	1.53459	-0.08353
P	-0.32347	-0.13449	0.39933
N	0.60283	-1.11055	-0.69014
C	-1.94820	1.72261	-0.70239
C	-2.65501	0.50147	-0.87556
C	-3.95432	0.52272	-1.40974
C	-4.51121	1.74614	-1.82295
C	-3.78708	2.94192	-1.70130
C	-2.50226	2.93760	-1.13043
N	-1.90936	-0.60894	-0.44221
C	-2.47674	-1.83147	-0.02571
C	-2.12242	-2.30298	1.27050
C	-2.65764	-3.50889	1.75303
C	-3.54330	-4.25285	0.95705
C	-3.86872	-3.81093	-0.33511
C	-3.32827	-2.61428	-0.83092
S	-0.94605	-1.32770	2.18417
H	-3.54444	-2.29275	-1.85347
H	-2.37817	-3.86365	2.74989
H	-4.53450	-4.40425	-0.96985

H	-3.96275	-5.18707	1.34356
H	-4.52897	-0.40150	-1.49749
H	-5.51985	1.75539	-2.24805
H	-1.93744	3.86627	-1.01193
H	-4.21961	3.88661	-2.04497
H	1.86943	2.00698	-0.62581
H	-1.54076	3.45499	1.60424
H	3.27426	3.85100	0.31798
H	-0.15575	5.32979	2.51175
H	2.25735	5.52963	1.87353
H	0.83806	0.32706	1.13529
C	2.02145	-1.15021	-0.73561
H	0.10572	-1.33128	-1.55358
C	2.70861	-0.90839	-1.94852
C	4.10225	-0.97069	-2.00417
C	4.85049	-1.24364	-0.83851
C	4.17755	-1.47744	0.37587
C	2.77337	-1.44835	0.41731
H	2.13891	-0.67308	-2.85467
H	4.63819	-0.79616	-2.94175
O	6.21550	-1.26420	-0.99964
H	4.72962	-1.70893	1.28998
H	2.25264	-1.68647	1.34967
C	7.00461	-1.54711	0.15579
H	8.05165	-1.51197	-0.17866
H	6.85083	-0.79354	0.95240
H	6.78657	-2.55239	0.56530

39

6TScoop

C	5.45594	-1.26478	-0.04625
C	4.88902	-0.34183	-0.94380
C	3.58223	0.12626	-0.76198

C	2.79980	-0.31365	0.33902
C	3.38627	-1.24036	1.23818
C	4.69290	-1.71137	1.04416
N	1.44925	0.04886	0.52055
C	1.00811	1.33806	0.21134
C	-0.34638	1.45078	-0.22115
C	-0.88043	2.68924	-0.61070
C	-0.10056	3.85321	-0.50857
C	1.22236	3.76455	-0.04433
C	1.77470	2.52390	0.30338
N	-1.08675	0.22643	-0.31091
P	-0.17621	-1.24378	-0.71047
N	-0.06842	-1.85235	1.18412
C	-2.45203	0.10921	0.03615
C	-3.10049	-1.10003	-0.33265
C	-4.44667	-1.32481	0.00194
C	-5.16436	-0.34418	0.70217
C	-4.52255	0.84645	1.08705
C	-3.17842	1.07802	0.76547
S	-2.09384	-2.26839	-1.22687
H	-2.68337	1.99807	1.08462
H	-4.92465	-2.26448	-0.29235
H	-5.06987	1.60515	1.65558
H	-6.21621	-0.51325	0.95197
H	-1.90085	2.73837	-1.00176
H	-0.52451	4.81759	-0.80419
H	2.80966	2.45424	0.65104
H	1.83456	4.66854	0.04280
H	3.14571	0.83106	-1.47664
H	2.81292	-1.56238	2.11567
H	5.46897	0.00993	-1.80451
H	5.12105	-2.42227	1.75976

H	6.47799	-1.62729	-0.19474
H	-0.98086	-1.81804	1.65464
H	0.35837	-2.77970	1.29267
H	0.64044	-0.97712	1.33955

39

6a

C	4.91874	-2.00818	-0.73032
C	4.70260	-0.66121	-1.06592
C	3.49940	-0.02018	-0.74124
C	2.48589	-0.72426	-0.04816
C	2.70470	-2.08233	0.28459
C	3.90555	-2.71450	-0.06102
N	1.23880	-0.14961	0.25178
C	0.96087	1.18504	0.58104
C	-0.38312	1.65998	0.47825
C	-0.68509	2.99271	0.80993
C	0.31528	3.87791	1.23404
C	1.63408	3.40930	1.35324
C	1.95209	2.08064	1.04634
N	-1.42917	0.81579	-0.02784
P	-1.92103	1.13612	-1.72069
N	-0.49870	0.58865	-2.53850
C	-1.84264	-0.35509	0.62655
C	-2.77904	-1.17598	-0.05588
C	-3.21538	-2.38275	0.50901
C	-2.75282	-2.77032	1.77936
C	-1.85802	-1.94470	2.47719
C	-1.39986	-0.74162	1.91249
S	-3.40210	-0.52050	-1.59649
H	-0.72598	-0.08360	2.47042
H	-3.92928	-3.00715	-0.03753
H	-1.51009	-2.22966	3.47525

H	-3.10339	-3.70763	2.22175
H	-1.72613	3.31910	0.72042
H	0.06567	4.91435	1.47917
H	2.97643	1.71975	1.17082
H	2.42574	4.07854	1.70628
H	3.32884	1.01649	-1.04396
H	1.92480	-2.63465	0.82180
H	5.47500	-0.09997	-1.60292
H	4.05229	-3.76647	0.20615
H	5.86045	-2.50096	-0.99034
H	-0.10265	-0.33803	-2.36559
H	-0.42005	0.88924	-3.51159
H	0.50142	-0.81378	0.49906

39

6b

C	-1.97496	-0.24149	0.59186
C	-2.73539	-1.32422	0.08311
C	-2.08782	0.10596	1.95228
C	-3.60924	-2.03805	0.91659
C	-2.95396	-0.62236	2.78319
H	-1.50147	0.94218	2.34380
C	-3.71543	-1.68718	2.27338
H	-4.19503	-2.86700	0.50674
H	-3.03739	-0.34529	3.83903
H	-4.39327	-2.24577	2.92602
C	-0.24982	1.48124	0.05999
C	-0.73634	2.79113	0.20094
C	1.12955	1.18944	0.30235
C	0.11293	3.83390	0.59818
H	-1.79258	2.97341	-0.01697
C	1.96880	2.24837	0.72819
C	1.46171	3.54576	0.86733

H	-0.27254	4.85250	0.69957
H	3.01203	2.04177	0.97690
H	2.13510	4.34244	1.20134
C	2.84502	-0.66598	0.08883
C	3.95415	0.05152	-0.41684
C	3.02799	-2.01102	0.48944
C	5.21341	-0.56141	-0.48726
H	3.82190	1.07060	-0.78829
C	4.28686	-2.61578	0.39713
H	2.17258	-2.57266	0.88192
C	5.39324	-1.89306	-0.08072
H	6.05990	0.00941	-0.88411
H	4.40450	-3.65802	0.71225
H	6.37922	-2.36371	-0.14177
S	-2.44441	-1.72979	-1.64009
N	-1.11469	0.39944	-0.32866
N	1.54797	-0.12740	0.15953
P	-1.38944	0.16633	-2.07506
N	-2.62363	1.36236	-2.36918
H	-2.74302	1.57353	-3.36316
H	-3.52726	1.25608	-1.89864
H	0.77757	-0.78934	0.05326

39

6c

C	2.48008	-0.00991	0.17100
C	2.72045	-1.19653	-0.58155
C	3.58001	0.75038	0.62065
C	4.03486	-1.56678	-0.91223
C	4.88779	0.37741	0.27473
H	3.40462	1.61862	1.26153
C	5.11584	-0.77187	-0.49910
H	4.20811	-2.47806	-1.49303

H	5.72982	0.98246	0.62561
H	6.13577	-1.06306	-0.76934
C	0.53105	1.54365	0.21623
C	1.13852	2.79205	-0.00185
C	-0.88522	1.43317	0.15904
C	0.33144	3.92492	-0.20730
H	2.22650	2.88148	-0.01735
C	-1.68519	2.56809	-0.03607
C	-1.06808	3.81941	-0.20814
H	0.80896	4.89750	-0.36284
H	-2.77411	2.47144	-0.05464
H	-1.68837	4.71016	-0.34754
C	-2.67364	-0.29166	0.05754
C	-3.37862	-1.00378	1.04835
C	-3.29479	-0.00715	-1.17753
C	-4.69315	-1.43198	0.80283
H	-2.88927	-1.19953	2.00722
C	-4.61319	-0.42305	-1.40748
H	-2.73454	0.53308	-1.94678
C	-5.31446	-1.13895	-0.42085
H	-5.23457	-1.98740	1.57564
H	-5.09062	-0.20032	-2.36738
H	-6.34192	-1.46728	-0.60810
S	1.29752	-2.17631	-1.01135
N	1.13938	0.30387	0.46224
N	-1.32352	0.10512	0.30857
P	-0.05153	-1.12030	0.43978
H	-1.03816	-2.16825	0.22632
N	0.27643	-1.59105	2.06498
H	0.19577	-2.60346	2.19588
H	1.16651	-1.23234	2.41945

39

6TSox

C	-0.423206	-1.559558	0.148327
C	0.701903	-2.043982	0.874105
C	-0.385023	-1.591086	-1.264535
C	1.786962	-2.622251	0.189355
C	0.706480	-2.162461	-1.932438
H	-1.193862	-1.135600	-1.838965
C	1.785039	-2.699333	-1.209853
H	2.636597	-2.999675	0.767044
H	0.713872	-2.174447	-3.027051
H	2.631315	-3.156343	-1.732309
C	-2.793158	-0.778187	0.428967
C	-3.428205	-1.391760	-0.668139
C	-3.537036	0.094967	1.274556
C	-4.754036	-1.051967	-0.980964
H	-2.900552	-2.139196	-1.262986
C	-4.861919	0.438543	0.942053
C	-5.459443	-0.124988	-0.194707
H	-5.235180	-1.520353	-1.845102
H	-5.412007	1.140182	1.575061
H	-6.484894	0.152853	-0.457990
C	-3.440417	0.938007	3.592013
C	-2.822904	1.944764	4.368477
C	-4.615709	0.320069	4.085479
C	-3.368854	2.327507	5.605644
H	-1.934680	2.452355	3.981104
C	-5.160971	0.718742	5.310346
H	-5.082635	-0.481983	3.506447
C	-4.540731	1.722766	6.078538
H	-2.877166	3.110521	6.192277
H	-6.068198	0.229005	5.679934
H	-4.970616	2.026080	7.038408

S	0.670827	-1.830535	2.632600
N	-1.480968	-0.990796	0.890463
N	-2.810406	0.519899	2.389115
P	-1.164380	-0.413447	2.584465
H	-0.555644	0.669468	3.632693
N	-0.102461	1.142669	2.336874
H	0.884443	1.010763	2.082028
H	-0.554304	1.958039	1.903270

43

6cto6aTS

C	3.091664	-0.185434	-2.741702
C	3.035879	-1.303628	-3.616402
C	4.348595	0.338824	-2.374252
C	4.219203	-1.850364	-4.140051
C	5.522637	-0.205742	-2.916252
H	4.402328	1.164499	-1.660101
C	5.462844	-1.291192	-3.805578
H	4.158753	-2.713604	-4.810501
H	6.490140	0.217767	-2.627674
H	6.380123	-1.712738	-4.228725
C	1.633581	1.569572	-1.677963
C	2.339542	2.731245	-2.045643
C	0.569879	1.643487	-0.733242
C	2.046878	3.955929	-1.423814
H	3.116767	2.673075	-2.812299
C	0.291067	2.886268	-0.123610
C	1.033141	4.030689	-0.454162
H	2.610485	4.850766	-1.706441
H	-0.517753	2.941323	0.612702
H	0.804813	4.984164	0.033517
C	-0.520401	0.015258	0.718161
C	0.214179	0.379159	1.879607

C	-1.601242	-0.893389	0.885333
C	-0.138205	-0.121719	3.140083
H	1.061963	1.063511	1.779365
C	-1.936511	-1.399845	2.147418
H	-2.170623	-1.206966	0.003130
C	-1.214345	-1.012980	3.289231
H	0.446175	0.181455	4.016292
H	-2.775657	-2.098507	2.238769
H	-1.483003	-1.402217	4.276155
S	1.412948	-1.957458	-3.970858
N	1.855780	0.288121	-2.246113
N	-0.204967	0.475209	-0.579104
P	0.459888	-0.824584	-2.226089
H	-2.095865	1.396684	-3.265895
N	0.518528	-2.173207	-1.152955
H	1.205512	-2.881001	-1.417462
H	0.505731	-1.952650	-0.152512
H	-1.036917	-0.191995	-2.802180
N	-2.086491	0.579826	-2.647783
H	-1.782019	0.842135	-1.674276
H	-2.999104	0.115028	-2.653648

51

7a

C	0.86172	1.95253	1.98201
C	0.99894	1.49931	0.65472
C	1.51012	2.37730	-0.33506
C	1.90629	3.67986	0.00611
C	1.76575	4.12597	1.33130
C	1.24106	3.26410	2.30884
N	0.63764	0.20580	0.21473
P	1.12989	-0.38649	-1.39819
S	1.54498	1.72614	-2.00124

C	-0.04072	-0.72656	1.07306
C	-1.46871	-0.79888	1.01126
C	-2.12896	-1.69471	1.88791
C	-1.39584	-2.50641	2.76163
C	0.00753	-2.45066	2.80244
C	0.67331	-1.55117	1.95715
N	-2.12630	0.05418	0.13371
C	-3.45911	0.04017	-0.31594
C	-4.22352	-1.14436	-0.42949
C	-5.53713	-1.08765	-0.91687
C	-6.10754	0.13098	-1.31754
C	-5.34020	1.30495	-1.23016
C	-4.03394	1.26559	-0.72885
N	2.65099	-1.17162	-1.07994
C	3.00312	-2.22842	-2.05795
C	2.91399	-3.65229	-1.48720
H	3.16481	-4.39285	-2.26715
H	0.46340	1.27517	2.74298
H	2.30837	4.34312	-0.76636
H	1.12992	3.60920	3.34187
H	2.06481	5.14521	1.59454
H	1.76413	-1.46851	1.96910
H	0.57356	-3.09203	3.48413
H	-3.22062	-1.72842	1.90031
H	-1.93609	-3.18592	3.42953
H	-3.77762	-2.10849	-0.17310
H	-3.44664	2.18701	-0.64390
H	-6.11286	-2.01579	-1.00004
H	-5.76411	2.26448	-1.54475
H	-7.13249	0.16510	-1.69926
C	3.76519	-0.49470	-0.39007
H	-1.52976	0.78561	-0.25509

H	4.01616	-2.02387	-2.45670
H	2.31445	-2.15133	-2.92109
H	3.60164	-3.80083	-0.63972
H	1.89074	-3.85756	-1.13209
C	4.65927	-1.42730	0.43960
H	4.38737	0.04875	-1.13249
H	3.32602	0.27026	0.27103
H	5.42146	-0.83109	0.96985
H	4.07263	-1.98361	1.18992
H	5.19267	-2.15895	-0.18982

51

7b

C	-2.94377	2.07237	-0.63111
C	-2.69176	0.77046	-0.13440
C	-3.65474	-0.24216	-0.36149
C	-4.84131	0.05747	-1.04517
C	-5.09048	1.35145	-1.53093
C	-4.12638	2.35280	-1.32565
N	-1.46330	0.52529	0.49968
C	-1.15396	-0.42477	1.47845
C	0.21423	-0.78137	1.69618
C	0.53860	-1.72770	2.68693
C	-0.45414	-2.33914	3.46431
C	-1.79554	-1.97140	3.26741
C	-2.14105	-1.01946	2.30122
N	1.26679	-0.24596	0.88213
P	2.01491	-1.42295	-0.27770
N	0.90226	-1.48590	-1.59962
C	0.44776	-2.77858	-2.15231
C	0.81504	-4.01910	-1.33248
H	0.36978	-4.90567	-1.81504
C	1.63046	1.10737	0.88689

C	1.02400	2.09922	1.69217
C	1.45938	3.43322	1.60674
C	2.49064	3.79736	0.72772
C	3.11612	2.81433	-0.06060
C	2.70459	1.47733	0.03237
S	3.51424	0.13154	-0.81824
H	0.24482	1.81316	2.40577
H	3.93847	3.08060	-0.73236
H	0.98652	4.18826	2.24305
H	2.82294	4.83778	0.66210
H	1.59561	-1.97346	2.83029
H	-0.18192	-3.07826	4.22345
H	-3.18429	-0.71621	2.18134
H	-2.58449	-2.41533	3.88366
H	-3.46024	-1.26329	-0.02321
H	-2.20368	2.86250	-0.45920
H	-5.57364	-0.74015	-1.21091
H	-4.29856	3.36753	-1.70011
H	-6.01908	1.57527	-2.06459
C	0.38846	-0.29868	-2.30505
H	-0.73176	1.21398	0.31704
H	-0.65691	-2.72336	-2.24470
H	0.83910	-2.90233	-3.18213
H	1.90441	-4.17641	-1.27692
H	0.42699	-3.95666	-0.30214
H	-0.71871	-0.35092	-2.32286
H	0.65481	0.58895	-1.70907
C	0.93487	-0.13159	-3.73298
H	0.52817	0.79032	-4.18301
H	2.03429	-0.05574	-3.71959
H	0.64948	-0.97156	-4.38817

51

7c

C	-3.51339	1.13570	0.41397
C	-2.47206	0.45870	-0.25703
C	-2.80608	-0.57021	-1.18318
C	-4.15047	-0.85610	-1.47123
C	-5.17493	-0.13542	-0.83714
C	-4.85312	0.84636	0.11372
N	-1.10602	0.68552	-0.03068
C	-0.48653	1.92003	0.18922
C	0.93029	1.79073	0.23290
C	1.74080	2.90877	0.48478
C	1.14158	4.16898	0.65013
C	-0.25072	4.31131	0.54063
C	-1.07113	3.19485	0.30453
N	1.35209	0.47255	-0.00003
P	0.05835	-0.71983	-0.35152
S	-1.43679	-1.46218	-1.87587
C	2.69872	0.14161	-0.33747
C	3.41417	0.88241	-1.30501
C	4.72593	0.51807	-1.63448
C	5.33054	-0.59656	-1.02563
C	4.61370	-1.34526	-0.08041
C	3.30497	-0.97694	0.27090
N	-0.07957	-1.69499	1.09635
C	-0.50634	-1.01733	2.34246
C	0.30050	-1.49388	3.55922
H	-0.04832	-0.97421	4.46832
H	-3.27080	1.86601	1.18948
H	-4.38967	-1.64771	-2.18829
H	-5.64743	1.38542	0.63997
H	-6.22061	-0.35689	-1.07259
H	-2.15023	3.31947	0.19989

H	-0.71234	5.29845	0.64294
H	2.82587	2.79149	0.54859
H	1.77059	5.03984	0.85897
H	2.93208	1.73372	-1.79466
H	2.73786	-1.53937	1.01852
H	5.27419	1.09820	-2.38417
H	5.07551	-2.21649	0.39586
H	6.35405	-0.87962	-1.29166
C	-0.58962	-3.08213	0.95487
H	1.02952	-1.55409	-1.03202
H	-0.35326	0.06571	2.21998
H	-1.59299	-1.16287	2.51704
H	0.19198	-2.57765	3.73618
H	1.37234	-1.27530	3.42375
H	-0.76374	-3.44574	1.98240
H	-1.57184	-3.09756	0.44062
C	0.37977	-4.04192	0.25381
H	-0.01970	-5.06983	0.29829
H	0.51716	-3.79024	-0.80993
H	1.36814	-4.03054	0.74238

39

SH

C	-5.51845	-1.28947	0.16447
C	-5.09361	-0.12779	0.82962
C	-3.75736	0.29083	0.75095
C	-2.83137	-0.44064	-0.02420
C	-3.26171	-1.60578	-0.69460
C	-4.59359	-2.03030	-0.58958
N	-1.46306	-0.05346	-0.11941
P	-0.19977	-1.19842	0.41441
N	-0.38072	-1.06590	2.13653
C	-1.00135	1.26772	-0.30057

C	0.41734	1.33879	-0.25446
C	1.08009	2.55976	-0.43704
C	0.31986	3.71725	-0.69157
C	-1.07911	3.64488	-0.77108
C	-1.75046	2.42183	-0.58048
N	1.00096	0.05659	-0.10970
C	2.37139	-0.12290	0.25725
C	3.22778	-0.92141	-0.54953
C	4.56654	-1.11860	-0.14989
C	5.05919	-0.52582	1.01908
C	4.22229	0.27866	1.81100
C	2.88807	0.46800	1.42962
S	2.66976	-1.68246	-2.06781
H	2.21894	1.09006	2.03283
H	5.22651	-1.73025	-0.77368
H	4.60352	0.74916	2.72263
H	6.10348	-0.68659	1.30579
H	2.17252	2.59888	-0.39878
H	0.83098	4.67384	-0.83799
H	-2.83919	2.36683	-0.65791
H	-1.66399	4.54540	-0.98339
H	-3.42168	1.17586	1.29884
H	-2.54362	-2.16579	-1.30209
H	-5.80297	0.45104	1.43077
H	-4.91229	-2.93714	-1.11417
H	-6.56107	-1.61528	0.23434
H	0.06514	-1.81065	2.67434
H	-0.35181	-0.15906	2.60866
H	1.46230	-1.04140	-2.03731

References

[1] CrysAlisPro, Agilent Technologies, Version 1.171.40.53.

[2] (a) G. M. Sheldrick in SHELXL97, *Programs for Crystal Structure Analysis (Release 97-2)*, Institut für Anorganische Chemie der Universität, Tammanstrasse 4, D-3400 Göttingen, Germany, 1998; (b) G. M. Sheldrick, *Acta Crystallogr. Sect. A* **1990**, *46*, 467–473; (c) G. M. Sheldrick, *Acta Crystallogr. Sect. A* **2008**, *64*, 112–122.

[3] a) A. J. Mountford, S. J. Lancaster, S. J. Coles, P. N. Horton, D. L. Hughes, M. B. Hursthouse, M. E. Light *Inorg Chem.* **2005**, *44*, 5921-5933; b) W. Huang, K. Besar, R. LeCover, A. M. Rule, P. N. Breysee, H. E. Katz, *J. Am. Chem. Soc* **2012**, *134*, 14650-14653.

[4] Gaussian 16, Revision C.01, Frisch, M. J.; Trucks, G. W.; Schlegel, H. B.; Scuseria, G. E.; Robb, M. A.; Cheeseman, J. R.; Scalmani, G.; Barone, V.; Petersson, G. A.; Nakatsuji, H.; Li, X.; Caricato, M.; Marenich, A. V.; Bloino, J.; Janesko, B. G.; Gomperts, R.; Mennucci, B.; Hratchian, H. P.; Ortiz, J. V.; Izmaylov, A. F.; Sonnenberg, J. L.; Williams-Young, D.; Ding, F.; Lipparini, F.; Egidi, F.; Goings, J.; Peng, B.; Petrone, A.; Henderson, T.; Ranasinghe, D.; Zakrzewski, V. G.; Gao, J.; Rega, N.; Zheng, G.; Liang, W.; Hada, M.; Ehara, M.; Toyota, K.; Fukuda, R.; Hasegawa, J.; Ishida, M.; Nakajima, T.; Honda, Y.; Kitao, O.; Nakai, H.; Vreven, T.; Throssell, K.; Montgomery, J. A., Jr.; Peralta, J. E.; Ogliaro, F.; Bearpark, M. J.; Heyd, J. J.; Brothers, E. N.; Kudin, K. N.; Staroverov, V. N.; Keith, T. A.; Kobayashi, R.; Normand, J.; Raghavachari, K.; Rendell, A. P.; Burant, J. C.; Iyengar, S. S.; Tomasi, J.; Cossi, M.; Millam, J. M.; Klene, M.; Adamo, C.; Cammi, R.; Ochterski, J. W.; Martin, R. L.; Morokuma, K.; Farkas, O.; Foresman, J. B.; Fox, D. J. Gaussian, Inc., Wallingford CT, **2016**.

[5] Becke, A. D. Density-functional exchange-energy approximation with correct asymptotic behavior. *Phys. Rev. A* **1998**, *38*, 3098−3100

[6] Perdew, J. P. Density-functional approximation for the correlation energy of the inhomogeneous electron gas. *Phys. Rev. B* **1986**, *33*, 8822−8824.

[7] Andrae, D.; Häußermann, U.; Dolg, M.; Stoll, H.; Preuß, H. Energy-adjusted ab initio pseudopotentials for the second and third row transition elements. *Theor. Chim. Acta.* **1990**, *77*, 123−141.

[8] Höllwarth, A.; Böhme, M.; Dapprich, S.; Ehlers, A. W.; Gobbi, A.; Jonas, V.; Köhler, K. F.; Stegmann, R.; Veldkamp, A.; Frenking, G. A set of d-polarization functions for pseudo-potential basis sets of the main group elements Al−Bi and f-type polarization functions for Zn, Cd, Hg. *Chem. Phys. Lett.* **1993**, *208*, 237−240.

[9] Hehre, W. J.; Ditchfield, R.; Pople, J. A. Self-Consistent Molecular Orbital Methods. XIV. An Extended Gaussian-Type Basis for Molecular Orbital Studies of Organic Molecules. Inclusion of Second Row Elements. *J. Chem. Phys.* **1972**, *56*, 2257.

[10] Hariharan, P. C.; Pople, J. A. The influence of polarization functions on molecular orbital hydrogenation energies. *Theor. Chim. Acta.* **1973**, *28*, 213−222.

[11] Weigend, F.; Ahlrichs, R. Balanced basis sets of split valence, triple zeta valence and quadruple zeta valence quality for H to Rn: Design and assessment of accuracy. *Phys. Chem. Chem. Phys.* **2005**, *7*, 3297−3305.

[12] Grimme, S.; Antony, J.; Ehrlich, S.; Krieg, H. A consistent and accurate ab initio parametrization of density functional dispersion correction (DFT-D) for the 94 elements H−Pu. *J. Chem. Phys.* **2010**, *132*, 154104.

[13] Knizia, G. Intrinsic atomic orbitals: An unbiased bridge between quantum theory and chemical concepts *J. Chem. Theory Comput.* **2013**, *9*, 4834-4843.

[14] Knizia, G.; Klein, J.E.M.N., Electron flow in reaction mechanisms --- revealed from first principles *Angew. Chem. Int. Ed.* **2015**, *54*, 5518-5522.

[15] Weigend, F., Hartree–Fock exchange fitting basis sets for H to Rn *J. Comput. Chem.* **2008**, *29*, 167-175.
